# Nonlinear L-shaped association between composite dietary antioxidant index and risk of dementia: a prospective cohort study

**DOI:** 10.3389/fpubh.2026.1839421

**Published:** 2026-07-03

**Authors:** Caimei Luo, Ruihan Wang, Feng Yang, Linyuan Qin, Yimeng Ren, Mengyao Guo, Hanlin Cai, Shiyu Feng, Nannan Li, Hui Gao, Yingying Tang, Qin Chen

**Affiliations:** Department of Neurology, West China Hospital of Sichuan University, Chengdu, China

**Keywords:** cohort study, dementia, dietary antioxidants, gray matter volume, inflammation

## Abstract

**Objective:**

Dietary intake of antioxidants may decrease risk of dementia. However, the relationship between the composite dietary antioxidant index (CDAI) and dementia, has not yet been explored. This study investigated the relationship between CDAI and risk of dementia and to explore potential mediators of the relationship.

**Methods:**

Data were extracted from the prospective UK Biobank on 157,742 dementia-free participants, who were followed up for a median of 13.39 yr. Potential associations between CDAI at enrollment and subsequent development of all-cause dementia, Alzheimer's dementia or vascular dementia were assessed using Cox proportional hazards regression. Potential non-linear associations were explored using restricted cubic spline analysis. Exploratory analyses were performed to assess whether blood inflammatory markers and cortical/subcortical gray matter volumes accounted for part of the associations, using multivariable linear regression and mediation analyses.

**Results:**

During follow-up, 791 people developed Alzheimer's dementia, 322 developed vascular dementia, and 709 developed other types of dementia. Higher CDAI was significantly associated with lower risk of all-cause dementia and Alzheimer's dementia, and the association was stronger in women than in men (p for interaction < 0.05). The association was nonlinear and L-shaped (nonlinear *p* ≤ 0.001), with inflection points at 1.579 for all-cause dementia and 0.848 for Alzheimer's dementia. At CDAIs below these inflection points, each unit increase in CDAI was associated with 6.3% lower risk of all-cause dementia (HR 0.937, 95% CI 0.907–0.968, *P* < 0.001) and 6.5% lower risk of Alzheimer's dementia (HR 0.935, 95% CI 0.897–0.974, *P* = 0.001). Exploratory pathway analyses suggested that neutrophil and lymphocyte percentages modestly accounted for the association between CDAI and risk of all-cause dementia and Alzheimer's dementia.

**Conclusions:**

CDAI may relate to risk of dementia in an L-shaped manner. Exploratory analyses suggested a possible role of systemic inflammation in this association.

## 1 Introduction

Dementia, a leading cause of disability and dependency in older adults, imposes a heavy burden on families and society as the global population ages (1). Alzheimer's dementia and vascular dementia are the most common subtypes, accounting for 60%−80% and 10%−20% of dementia cases, respectively (2). Fourteen modifiable risk factors for dementia have been identified by the Lancet Commission, including oxidative stress (3). Oxidative stress could trigger a cascade of cellular damage in the brain that underlie the dementia pathogenesis and progression (4).

Dietary modifications, particularly the intake of antioxidants, have been reported to lower oxidative stress levels and showed potential in alleviating dementia risk (5, 6). One study linked a diet rich in vitamins C or E to lower risk of Alzheimer's dementia (7). Another study linked Mediterranean diet-containing fruits rich in polyphenols, nuts rich in vitamin E and seafood rich in *n*-3 fatty acids- to lower risk of dementia (8) and slower age-related cognitive decline (9). Protection against dementia is likely to require adequate intake of various antioxidants rather than just one or two: one trial found no protective effects of dietary supplementation with vitamin E or selenium against dementia (10), whereas another study found that combined supplementation with vitamins E and C protected against Alzheimer's dementia more than supplementation with either vitamin on its own (11). These observations highlight the need to assess antioxidant consumption holistically in order to take into account the inherent complexity of diets and potential synergies among individual antioxidants (12).

The composite dietary antioxidant index (CDAI) comprehensively reflects dietary antioxidant exposure by integrating six essential nutrients, including manganese, selenium, zinc, vitamins A, C and E (13). Lower CDAI indicates smaller systemic antioxidant capacity, which has been associated with higher prevalence of diabetes (14), hypertension (15), depression (16) and coronary heart disease (17). Conversely, higher CDAI correlated positively with cognitive performance involving memory, language, attention and executive function in a sample of primarily Caucasian individuals at least 60 years old (18). Higher CDAI was linked to lower risk of cognitive impairment later in life in a study of Chinese individuals (19). However, the association between CDAI and the risk of dementia has not been validated in longitudinal adult cohort.

In this study, we aimed to use the UK Biobank cohort to prospectively investigate the relationship between the CDAI and the risk of developing all-cause dementia, Alzheimer's dementia and vascular dementia. Subgroup and interaction analyses were performed to elucidate potential variations in their relationship across different populations, such as participants with different age, sex, body mass index (BMI), and Apolipoprotein E epsilon4 allele (APOEε4) carrier status. We also explored whether any observed relationships between CDAI and dementia risk may involve modulation of inflammatory responses, given that higher CDAI has been linked to lower levels of pro-inflammatory factors in the blood (20); or may involve changes in gray matter volume in the brain, given that an antioxidant-rich diet may be connected to brain atrophy (21).

## 2 Methods

### 2.1 Study population

The data for this study came from the prospective, population-based UK Biobank, which follows up more than 500,000 participants, who were recruited between 2006 and 2010 from 22 assessment centers around the United Kingdom when the individuals were 37–73 years old (22). Details about the ethics procedures for participation in the UK Biobank, inclusion and exclusion criteria, and the assessments performed have been described in detail elsewhere (22). Informed consent was obtained from all participants prior to data collection. This research was conducted under UK Biobank application number 98,130.

**Figure 1:**
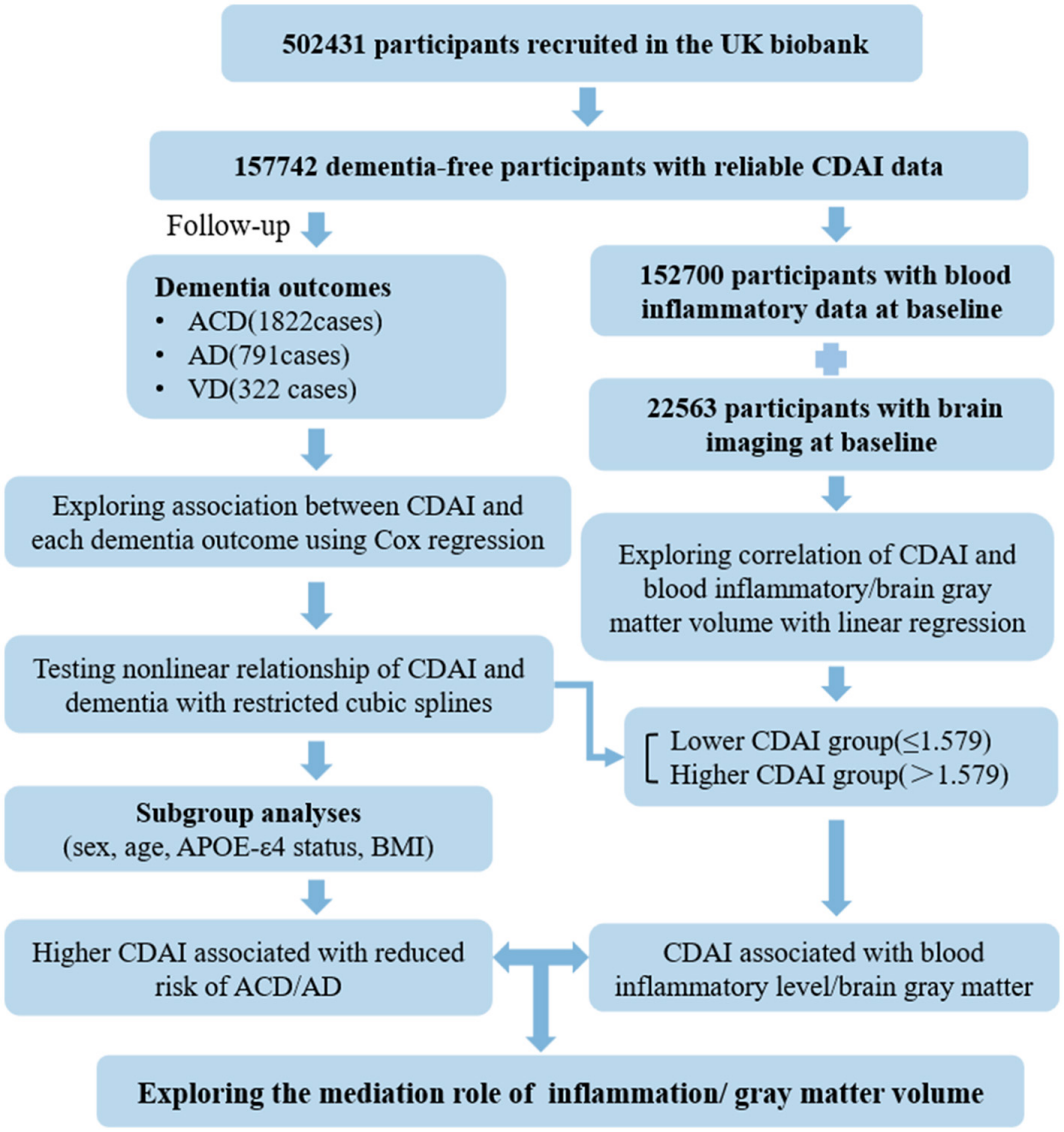
Workflow of participant enrollment and analysis. ACD, All-cause dementia; AD, Alzheimer's dementia; VD, vascular dementia; CDAI, composite dietary antioxidant index; BMI, Body mass index.

In this study, dementia-free participants with reliable dietary assessment records and complete baseline information were included. Participants were excluded if they met any of the following exclusion criteria: (1) missing 24-h dietary assessment data(*n* = 29,1420); (2) self-reported atypical dietary intake on the assessment day due to illness, fasting, or other exceptional circumstances(*n*=18,624); (3) participants diagnosed with dementia before their last valid dietary assessment or study withdrawal(*n* = 62); or (4) incomplete baseline covariate data(*n* = 34,522) (see Methods for details) (Figure 1, Supplementary Figure S1). Follow-up time was defined as the interval in years from date of enrollment until first diagnosis of dementia, death, loss to follow-up, or censoring date (December 17, 2022), whichever occurred first.

### 2.2 Dietary assessment and CDAI calculation

Dietary intake was assessed at enrollment in the UK Biobank using the Oxford Web-based 24-h Dietary Questionnaire (WebQ), which captures data on the consumption of 206 foods and 32 beverages during the previous 24 h (23). The Oxford WebQ was first administered at the assessment center between April 2009 and September 2010. In addition, participants with available email addresses were subsequently invited to complete the questionnaire online on up to four additional occasions at approximately 3–4-month intervals between February 2011 and June 2012, with the final dietary assessment cycle completed on June 15, 2012 (24). Nutrient intake was estimated as described (25). To reduce measurement error and within-person variability, daily intake of nutrients and energy were averaged across the multiple assessments when available (up to a maximum of five). The method of averaging repeated measurements has been previously validated as superior to reliance on a single dietary recall (26).

Intake of the six dietary antioxidants manganese, selenium, zinc as well as vitamins A, C and E were standardized by subtracting the global mean and dividing by the global standard deviation (13). These standardized intake values for the six antioxidants were summed to generate the CDAI, as described next:


$$\begin{array}{l} \text{CDAI}=\overset{6}{\underset{i=1}{\sum }}\frac{Xi-\mu i}{Si} \end{array}$$

(represents the individual's daily intake of the antioxidant. represented the mean daily intake of the antioxidant for the entire cohort. represented the standard deviation of antioxidant across the entire cohort),

### 2.3 Profiles of inflammatory markers in blood

Data on inflammatory markers were derived from baseline hematological assays of blood samples collected from UK Biobank participants at recruitment using standardized protocols and analyzed by methods described in detail previously (27). For the present study, data were extracted on counts or percentages of leukocytes, neutrophils, monocytes, lymphocytes and platelets; level of C-reactive protein; and ratios of neutrophils to lymphocytes, lymphocytes to monocytes, and platelets to lymphocytes (28). Counts of neutrophils and platelets were multiplied together and then divided by lymphocyte count to yield the systemic immune inflammation index (SII) (28).

### 2.4 Gray matter volume in the brain

Structural brain MRI was acquired during the UK Biobank imaging visit (cycle 2), beginning in 2014 during follow-up, using a Siemens Skyra 3 T scanner with a standard 32-channel head coil. Gray matter volumes of 62 cortical regions, defined according to the Desikan-Killiany-Tourville parcellation, and of 48 subcortical nucleus, defined according to ASEG atlases, were extracted using surface-based analysis in FreeSurfer. Details of data acquisition and processing have been described in detail elsewhere (29).

### 2.5 Diagnosis of dementia

Dementia was diagnosed based on hospital inpatient records and death registration data as described (30) according to the 10th revision of the International Classification of Diseases (ICD-10) for Alzheimer's dementia (F00 and G30), vascular dementia (F01and I67.3), and other subtypes of dementia (frontotemporal dementia, dementia with Lewy bodies, Parkinson's disease dementia, etc.) (Supplementary Table S1).

### 2.6 Assessment of covariates

Data about participant characteristics at enrollment were extracted for the following potential covariates: age, sex, education level, ethnicity (white, mixed, Asian, or other), consumption of vitamin and mineral supplements (yes or no), smoking status (never, former, or current), alcohol consumption (never, former, or current), and BMI. Socioeconomic status at enrollment was assessed using the Townsend Deprivation Index (TDI) based on the postal code where participants lived. Status as a carrier of the Alzheimer's disease risk allele APOEε4 was assessed based on genotyping of the single-nucleotide polymorphisms rs7412 and rs429358(31). Presence of the following comorbidities at enrollment was determined based on the corresponding ICD-10 codes: cardiovascular disease, I20-I25 and I50; diabetes, E10-E14; hyperlipidemia, E78; hypertension, I10 and I15; stroke, I60-I64 and I69; and cancer, C00-C97. Other definitions and procedures for covariates have been described in detail elsewhere (32).

### 2.7 Statistical analysis

Data were analyzed statistically using R software (Version 4.3.1; R Foundation for Statistical Computing, Vienna, Austria), and results associated with two-sided *p* < 0.05 were considered significant. Continuous variables were expressed as mean ± standard deviation if normally distributed, or as median (interquartile range, IQR) otherwise. Intergroup differences were assessed for significance using, respectively, Student's *t* test or the Mann-Whitney U test. Categorical variables were reported as *n* (%), and differences were assessed using the chi-squared test.

Potential associations of CDAI with risk of all-cause dementia, Alzheimer's dementia, or vascular dementia were explored using Cox proportional hazards modeling. For this modeling, CDAI was categorized into quartiles: quartile 1, CDAI < −2.545; quartile 2, −2.545 ≤ CDAI < −0.349; quartile 3, −0.349 ≤ CDAI < 2.132; and quartile 4, CDAI ≥2.132.

The proportional hazards assumption was tested using Schoenfeld residuals. CDAI satisfied this assumption whether modeled continuously or by quartiles, whereas covariates of age, cardiovascular disease, and hyperlipemia violated the assumption. Therefore, these variables were incorporated into extended Cox models with time-dependent effects by including interactions with follow-up time, using a linear time-transformation function. Model 1 was adjusted for age, sex, ethnicity, education level, APOEε4 status, use of vitamin and mineral supplements, and average daily energy intake. Model 2 included these variables as well as BMI, Townsend deprivation index, alcohol consumption, smoking status, and history of diabetes, hypertension and hyperlipidemia. Model 3 included the same variables as Model 2 in addition to history of stroke, cancer and cardiovascular disease. Risk was reported in terms of hazard ratios (HRs) and associated 95% confidence intervals (CIs).

The potential linearity trend of associations between CDAI and dementia was explored by treating the median CDAI in each CDAI quartile as a continuous variable in the regression model. Nonlinearity in the association between CDAI and dementia risk was assessed using a multivariable restricted cubic spline in the Cox model. The spline model with three knots at the 10th, 50th, and 90th CDAI percentiles was selected based on the lowest Akaike Information Criterion (33) and nonlinearity was tested using likelihood ratio tests for the nonlinear spline terms. Potential inflection points were then identified using a recursive search algorithm. Candidate CDAI values between the 5th and 95th percentiles were evaluated in two-piece Cox models, and the search range was iteratively narrowed around the value yielding the highest log-likelihood. The final inflection point was defined as the CDAI value that maximized the model log-likelihood. The two-piece Cox model was compared with the single-linearity model using a likelihood ratio test. For graphical presentation, HRs from the spline models were estimated using the identified inflection point as the reference value. Subgroup and interaction analyses were performed for sex (female or male), age (< 60 or ≥60 years), APOEε4 status (carrier or non-carrier) and BMI (< 25 or ≥25 kg/m^2^). Interactions between grouping variables and CDAI were tested using likelihood ratio tests.

The robustness of associations detected between CDAI and risk of dementia was evaluated by repeating the primary analysis under each of the following conditions: (1) adjusting risk models by physical activity in terms of “metabolic equivalent minutes per week” based on data from the International Physical Activity Questionnaire (34) (2) excluding participants with implausible daily energy intake, which was defined as < 800 or > 4,200 kcal for men or as < 600 or > 3,500 kcal for women; (3) excluding cases of dementia diagnosed within the first 5 years of follow-up in order to mitigate potential early-onset effects; (4) reassigning participants with missing APOEε4 data to a new “missing” category; and (5) applying Fine-Gray subdistribution hazard modeling with death as a competing event in order to account for mortality-related censoring. To explore possible concerns over temporality, we conducted analyses using only baseline dietary data (cycle 0) and landmark analyses with follow-up beginning on June 15, 2012, the end date of the last cycle of dietary assessment after excluding those who developed dementia or died prior to this date. We additionally adjusted for the number of completed dietary assessments (1–5) to account for potential differences in exposure measurement precision across participants. A sensitivity analysis was also performed using multiple imputation by chained equations (MICE, m = 5 imputed datasets) to address missing baseline covariates.

The ability of levels of blood inflammatory markers or gray matter volumes to help explain observed associations between CDAI and risk of dementia was assessed through mediation analyses. First, potential associations between CDAI and either levels of inflammatory markers or gray matter volumes were assessed using linear regression that adjusted for the same covariates as in Model 3 above. Associations were determined to be significant after correction for false discovery rate in the case of inflammatory markers or after Bonferroni correction in the case of gray matter volumes. Second, potential associations between dementia and either inflammatory markers or gray matter volumes were assessed using Cox proportional hazards modeling. Variables that emerged as significantly associated with both CDAI and dementia were entered into the “mediation” package in R for 1,000 bootstrap iterations with the same covariates as in Model 3 above. Mediation analyses were repeated for subgroups whose CDAIs were “low” or “high” based on the inflection point in the restricted cubic spline model linking CDAI to all-cause dementia.

## 3 Results

### 3.1 Participant characteristics

The final analysis included 157,742 participants (55.36% women, 91.11% of white ethnicity), who were a median of 57 (IQR 50-63) years old at enrollment. Just over one quarter of participants (28.16%) carried the APOEε4 allele (Table 1). As to dietary assessment, 72,183 (45.76%) and 85,559 (54.24%) participants completed one and two or more 24-h recalls, respectively. Specifically, 41,500 (26.31%), 28,675 (18.18%), 13,479 (8.54%) and 1,905 (1.21%) participants completed two, three, four and five assessments, respectively. The frequency distribution of assessments was different among CDAI quartiles (*P* < 0.001). The percentages of patients who completed a single recall were 55.47%, 40.91%, 39.13%, and 47.53% in Quartiles 1–4, respectively (Supplementary Table S2). Participants excluded due to missing baseline covariate data were generally similar to those included in the final analysis with respect to age and education, although they had slightly higher BMI, TDI, and prevalence of several cardiometabolic conditions (Supplementary Table S3).

**Table 1:** Clinicodemographic characteristics of participants at baseline (enrollment in UK Biobank)^1^.

Characteristic	All *N* = 157,742	Developed dementia during follow-up		*P*
		No *n* = 155,920	Yes *n* = 1,822	
**Age, year**	57.00 (50.00–63.00)	57.00 (50.00–62.00)	65.00 (62.00–67.00)	< 0.001
**Female**	87,319 (55.36)	86,458 (55.45)	861 (47.26)	< 0.001
**Ethnicity**				0.022
White	143,722 (91.11)	142,035 (91.09)	1,687 (92.59)	
Mixed	4,965 (3.15)	4,905 (3.15)	60 (3.29)	
Asian	6,592 (4.18)	6,541 (4.20)	51 (2.80)	
Other	2,463 (1.56)	2,439 (1.56)	24 (1.32)	
**Education, yr**	15.00 (10.00–20.00)	15.00 (10.00–20.00)	13.00 (10.00–20.00)	< 0.001
**TDI**	−2.36 (−3.75 to 0.02)	−2.36 (−3.75 to 0.02)	−2.37 (−3.76 to 0.07)	0.637
**BMI, kg/m** ^**2**^	26.15 (23.67–29.19)	26.15 (23.66–29.19)	26.49 (23.98–29.51)	0.001
**APOEε4 carrier**				< 0.001
No	113,320 (71.84)	112,487 (72.14)	833 (45.72)	
Yes	444,422 (28.16)	43,433 (27.86)	989 (54.28)	
**Smoking history**				< 0.001
Never	89,953 (57.03)	89,082 (57.13)	871 (47.80)	
Previous	56,608 (35.89)	55,772 (35.77)	836 (45.88)	
Current	11,181 (7.09)	11,066 (7.10)	115 (6.31)	
**Alcohol consumption**				< 0.001
Never	5,111 (3.24)	5,025 (3.22)	86 (4.72)	
Previous	4,722 (2.99)	4,632 (2.97)	90 (4.94)	
Current	147,909 (93.77)	146,263 (93.81)	1,646 (90.34)	
**Diabetes**	3,444 (2.18)	3,331 (2.14)	113 (6.20)	< 0.001
**Hypertension**	28,023 (17.77)	27,376 (17.56)	647 (35.51)	< 0.001
**Hyperlipidemia**	4,238 (2.69)	4,106 (2.63)	132 (7.24)	< 0.001
**Cardiovascular disease**	5,174 (3.28)	4,970 (3.19)	204 (11.20)	< 0.001
**Stroke**	533 (0.34)	517 (0.33)	16 (0.88)	< 0.001
**Cancer**	11,833 (7.50)	11,642 (7.47)	191 (10.48)	< 0.001
**Vitamin and mineral supplements**	51,585 (32.70)	50,911 (32.65)	674 (36.99)	< 0.001
**Daily energy intake, kcal**	1,981.11 (1,652.24–2,358.77)	1,980.86 (1,652.13–2,358.27)	2,000.05 (1,658.59–2,406.25)	0.105
**CDAI scores**	−0.35 (−2.54 to 2.13)	−0.35 (−2.54 to 2.13)	−0.27 (−2.56 to 2.47)	0.192
**Follow-up, yr**	13.39 (12.80–14.19)	13.40 (12.82–14.19)	10.70 (8.42–12.20)	< 0.001

CDAI, composite dietary antioxidant index; TDI, Townsend deprivation index; BMI, body mass index. ^1^Values are *n* (%), mean ± SD or median (interquartile range), unless otherwise noted.

During follow-up lasting a median of 13.39 (IQR 12.80-14.19) years, 1,822 participants (1.16%) developed all-cause dementia, comprising 791 who developed dementia related to Alzheimer's dementia, 322 who developed vascular dementia, and 709 who developed other types of dementia. Compared to those who did not develop dementia during follow-up, participants with dementia were older and less educated; had a higher BMI; were more likely to be an APOEε4 carrier; and were more likely to have a history of smoking, alcohol consumption, diabetes, hypertension, hyperlipidemia, cardiovascular disease, stroke and cancer (Table 1). Participants in higher CDAI quartiles tended to be older and more educated, were less likely to have a smoking history, and had lower BMI, lower frequencies of cardiometabolic comorbidities and cancer, and higher rates of supplement use. (Supplementary Table S4).

### 3.2 Relationships between CDAI and risk of dementia

**Figure 2:**
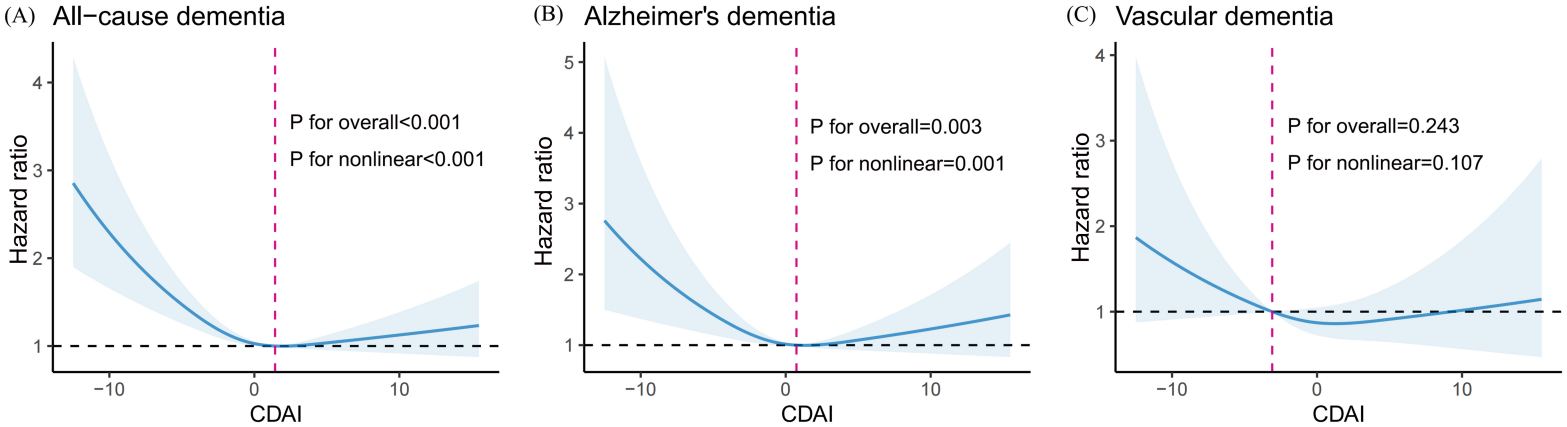
Restricted cubic spline analysis of relationships between composite dietary antioxidant index (CDAI) treated as a continuous variable and risk of **(A)** all-cause dementia, **(B)** Alzheimer's dementia or **(C)** vascular dementia. Solid lines show the results of Cox regression, while blue shading shows the corresponding 95% confidence intervals. The red vertical dashed line indicates the inflection point. The regression adjusted for the same variables as Model 3 in Table 2.

The numbers of dementia events across CDAI quartiles are presented in Table 2. The inverse associations with all-cause dementia and Alzheimer's dementia were most apparent in the middle CDAI quartiles and appeared to plateau in the highest quartile. In Model 3, the risk of all-cause dementia was significantly lower in the second CDAI quartile (HR 0.83, 95% CI 0.72–0.95, *P* = 0.008), third quartile (HR 0.78, 95% CI 0.67–0.90, *P* = 0.001) and fourth quartile (HR 0.82, 95% CI 0.69–0.97, *P* = 0.019), when compared to the lowest CDAI quartile. As CDAI increased, risk of all-cause dementia decreased significantly (P for trend = 0.016); and after adjusting for all confounders, each 1-unit increase in CDAI was associated with a 2% reduction in risk of all-cause dementia (HR 0.98, 95% CI: 0.96–0.99, *P* = 0.020) (Table 2). A L-shaped nonlinear association between CDAI and risk of all-cause dementia was revealed by restricted cubic spline analysis of Model 3 (*P* for nonlinearity < 0.001; Figure 2A). Two-piecewise Cox regression identified an inflection point at 1.579 (*P* for likelihood ratio test < 0.001; Table 3), below which each 1-unit increase in CDAI was associated with a 6.3% reduction in risk (HR 0.937, 95% CI 0.907–0.968, *P* < 0.001) and above which CDAI was not associated with risk (HR 1.023, 95% CI 0.991–1.057, P = 0.160). Model 3 also showed risk of Alzheimer's dementia to be significantly lower in the second CDAI quartile (HR 0.80, 95% CI 0.65–0.98, *P* = 0.031) and third quartile (HR 0.71, 95% CI 0.57–0.89, *P* = 0.003), when compared to the lowest CDAI quartile. A similar L-shaped nonlinear association between CDAI and risk of Alzheimer's dementia was observed (*P* for nonlinearity = 0.001; Figure 2B). Two-piecewise Cox regression identified an inflection point at 0.848 (P for likelihood ratio test < 0.001; Table 3), below which each 1-unit increase in CDAI was associated with a 6.5% reduction in risk (HR 0.935, 95% CI 0.897–0.974, *P* = 0.001) and above which CDAI was not associated with risk (HR 1.027, 95% CI 0.991–1.065, *P* = 0.148). None of the Cox regression models indicated a significant association between CDAI quartile and risk of vascular dementia (Table 2). A similar lack of association was observed in the restricted cubic spline analysis (Figure 2C).

**Table 2:** Cox regression to detect associations between CDAI and dementia in the entire sample of participants.

Type of dementia	CDAI level ^1^	N_total_ (N_dementia_)^2^	Model 1^3^		Model 2^4^		Model 3^5^	
			HR (95%CI)	*P*	HR (95%CI)	*P*	HR (95%CI)	*P*
All-cause dementia	Per 1-unit increase	157,742 (1,822)	0.98 (0.96–0.99)	**0.021**	0.98 (0.96–0.99)	**0.020**	0.98 (0.96–0.99)	**0.020**
	Quartile 1	39,436 (458)	Reference		Reference		Reference	
	Quartile 2	39,435 (434)	0.81 (0.71–0.93)	**0.002**	0.83 (0.72–0.95)	**0.006**	0.83 (0.72–0.95)	**0.008**
	Quartile 3	39,435 (427)	0.75 (0.65–0.87)	**< 0.001**	0.77 (0.67–0.90)	**0.001**	0.78 (0.67–0.90)	**0.001**
	Quartile 4	39,436 (503)	0.81 (0.68–0.96)	**0.015**	0.81 (0.69–0.96)	**0.018**	0.82 (0.69–0.97)	**0.019**
	P for trend	157,742 (1,822)		**0.012**		**0.014**		**0.016**
Alzheimer's dementia	Per 1–unit increase	157,742 (791)	0.99 (0.96–1.02)	0.365	0.99 (0.96–1.01)	0.309	0.99 (0.96–1.01)	0.285
	Quartile 1	39,436 (210)	Reference		Reference		Reference	
	Quartile 2	39,435 (189)	0.79 (0.65–0.97)	**0.027**	0.80 (0.65–0.98)	**0.032**	0.80 (0.65–0.98)	**0.031**
	Quartile 3	39,435 (173)	0.71 (0.56–0.88)	**0.002**	0.71 (0.57–0.89)	**0.003**	0.71 (0.57–0.89)	**0.003**
	Quartile 4	39,436 (219)	0.85 (0.66–1.10)	0.207	0.84 (0.65–1.08)	0.174	0.83 (0.64–1.08)	0.163
	P for trend	157,742 (791)		0.138		0.117		0.110
Vascular dementia	Per 1–unit increase	157,742 (322)	0.99 (0.95–1.03)	0.688	0.99 (0.95–1.03)	0.683	0.99 (0.95–1.03)	0.658
	Quartile 1	39,436 (73)	Reference		Reference		Reference	
	Quartile 2	39,435 (83)	1.00 (0.72–1.39)	0.977	1.07 (0.77–1.48)	0.692	1.07 (0.77–1.49)	0.673
	Quartile 3	39,435 (73)	0.84 (0.59–1.21)	0.354	0.90 (0.63–1.29)	0.570	0.90 (0.63–1.30)	0.580
	Quartile 4	39,436 (93)	0.99 (0.66–1.49)	0.959	1.02 (0.68–1.53)	0.938	1.01 (0.67–1.53)	0.949
	P for trend	157,742 (322)		0.732		0.821		0.808

^1^Quartiles were defined as follows: quartile 1, CDAI < −2.545; quartile 2, −2.545 ≤ CDAI < −0.349; quartile 3, −0.349 ≤ CDAI < 2.132; and quartile 4, CDAI ≥2.132. ^2^N_total_ (N_dementia_) indicates the total number of participants and the number of incident dementia cases. ^3^Model 1 was adjusted for age, sex, ethnicity, education level, APOEε4 status, daily energy intake, and vitamin and mineral supplements. ^4^Model 2 adjusted for the same variables as well as diabetes, hypertension, hyperlipidemia, body mass index, Townsend deprivation index, alcohol consumption, and smoking status. ^5^Model 3 adjusted for the same variables as Model 2 in addition to cardiovascular disease, stroke and cancer. Bold values indicate statistical significance (*P* < 0.05). CDAI, composite dietary antioxidant index; CI, confidence interval; HR, hazard ratio.

**Table 3:** Analysis of threshold effects in associations between CDAI and dementia detected through Cox regression in the entire sample of participants^1^.

Analysis	All-cause dementia		Alzheimer's dementia	
	HR (95% CI)	*P*	HR (95% CI)	*P*
Standard Cox regression	0.977 (0.959–0.996)	0.019	0.985 (0.958–1.012)	0.271
Two-piecewise Cox regression				
Inflection point	1.579		0.848	
Participants whose CDAI ≤ inflection point	0.937 (0.907–0.968)	**< 0.001**	0.935 (0.897–0.974)	**0.001**
Participants whose CDAI > inflection point	1.023 (0.991–1.057)	0.160	1.027 (0.991–1.065)	0.148
P for likelihood ratio test		**< 0.001**		**0.001**

^1^Regression was adjusted for age, sex, ethnicity, education level, APOEε4 status, daily energy intake, and vitamin and mineral supplements, diabetes, hypertension, hyperlipidemia, body mass index, Townsend deprivation index, alcohol consumption, smoking status, and history of cardiovascular disease, stroke and cancer. Bold values indicate statistical significance (*P* < 0.05). CDAI, composite dietary antioxidant index; CI, confidence interval; HR, hazard ratio.

The observed associations between higher CDAI and lower risks of all-cause dementia and Alzheimer's dementia persisted in the sensitivity analyses that we conducted, whether we excluded participants with missing physical activity data and adjusted data for the remaining participants according to physical activity, whether we excluded participants with implausible daily energy intake, whether we excluded cases of dementia diagnosed within the first 5 years of follow-up, whether we included participants with missing APOE genotype data, or whether we treated mortality as an event competing with dementia (Supplementary Tables S5–S9). The associations between CDAI and dementia risk remained materially unchanged in baseline-only analyses, landmark analyses, and analyses additionally adjusting for the number of completed dietary assessments (Supplementary Tables S10–S12). Main results from multiple imputation analyses were largely consistent with those from the complete-case analysis (Supplementary Tables S13).

### 3.3 Relationships between CDAI and risk of dementia in subgroups

Subgroup analyses showed that higher CDAI was associated with lower risk of all-cause dementia and Alzheimer's dementia in women but not in men (Supplementary Tables S14, S15), and sex-specific effects were observed for both all-cause dementia (P for interaction = 0.017) and Alzheimer's dementia (*P* for interaction = 0.011; Supplementary Table S22). Risk of either outcome did not significantly interact with age, BMI, or APOEε4 carrier status (all P for interaction > 0.05, Supplementary Table S22). However, significant associations with CDAI were still observed within some specific subgroups. In the subgroup of individuals older than 60 years, risk of Alzheimer's dementia was significantly lower for those whose CDAI was in the second quartile (HR 0.79, 95% CI 0.64–0.99, *P* = 0.040) or third quartile (HR 0.76, 95% CI 0.60–0.97, *P* = 0.026) than for those whose CDAI was in the first quartile (Supplementary Tables S16, S17). In the subgroup of individuals with BMI ≥ 25 kg/m^2^, risk of Alzheimer's dementia was significantly lower for those whose CDAI was in the third quartile than for those in the first quartile (HR 0.67, 95% CI 0.50–0.89, *P* = 0.006) (Supplementary Tables S20, S21).

### 3.4 Exploratory pathway analysis of blood inflammatory markers

**Figure 3:**
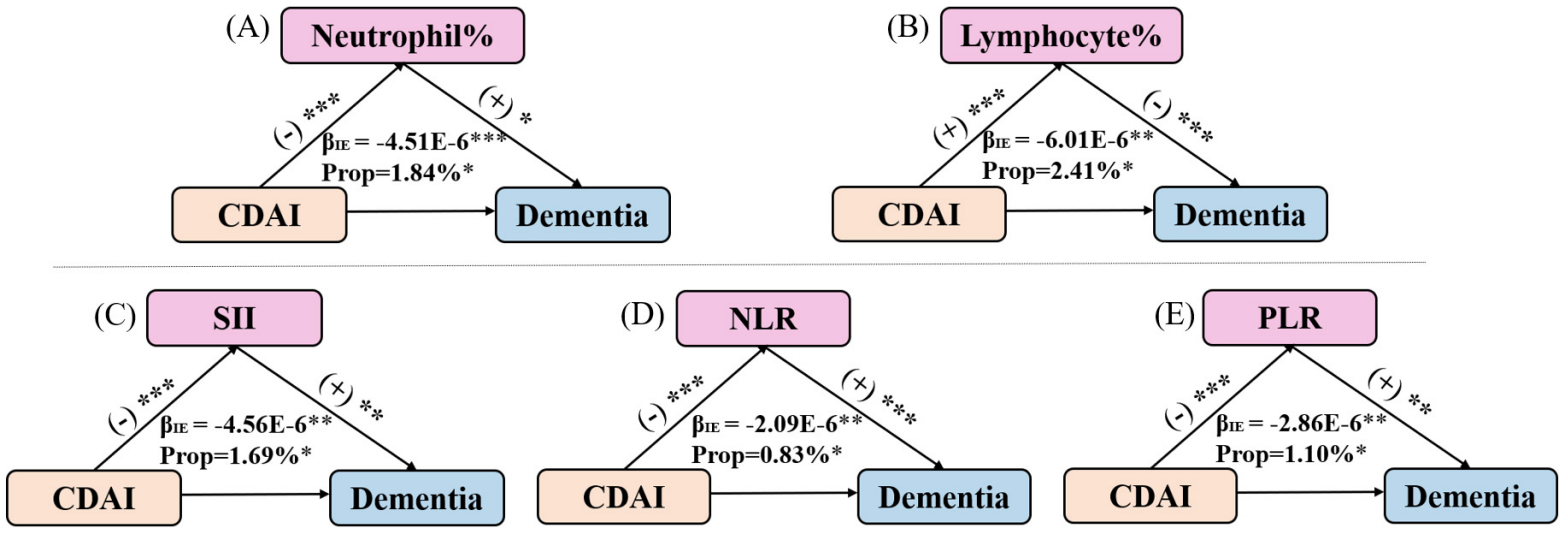
Potential that levels of blood inflammatory markers mediate relationships between composite dietary antioxidant index (CDAI) and risk of all-cause dementia in the entire sample of all participants using the Model 3 described in Table 2. Mediation effects were quantified in terms of indirect effects (βIE) and “proportion of observed effects mediated” (Prop) by the following inflammatory markers: **(A)** neutrophil percentage, **(B)** lymphocyte percentage, **(C)** systemic immune inflammation index (SII), **(D)** ratio of neutrophils to lymphocytes (NLR), and **(E)** ratio of platelets to lymphocytes (PLR). (+), positive association, (–) negative association, *P < 0.05, **P < 0.01, ***P < 0.001.

**Figure 4:**
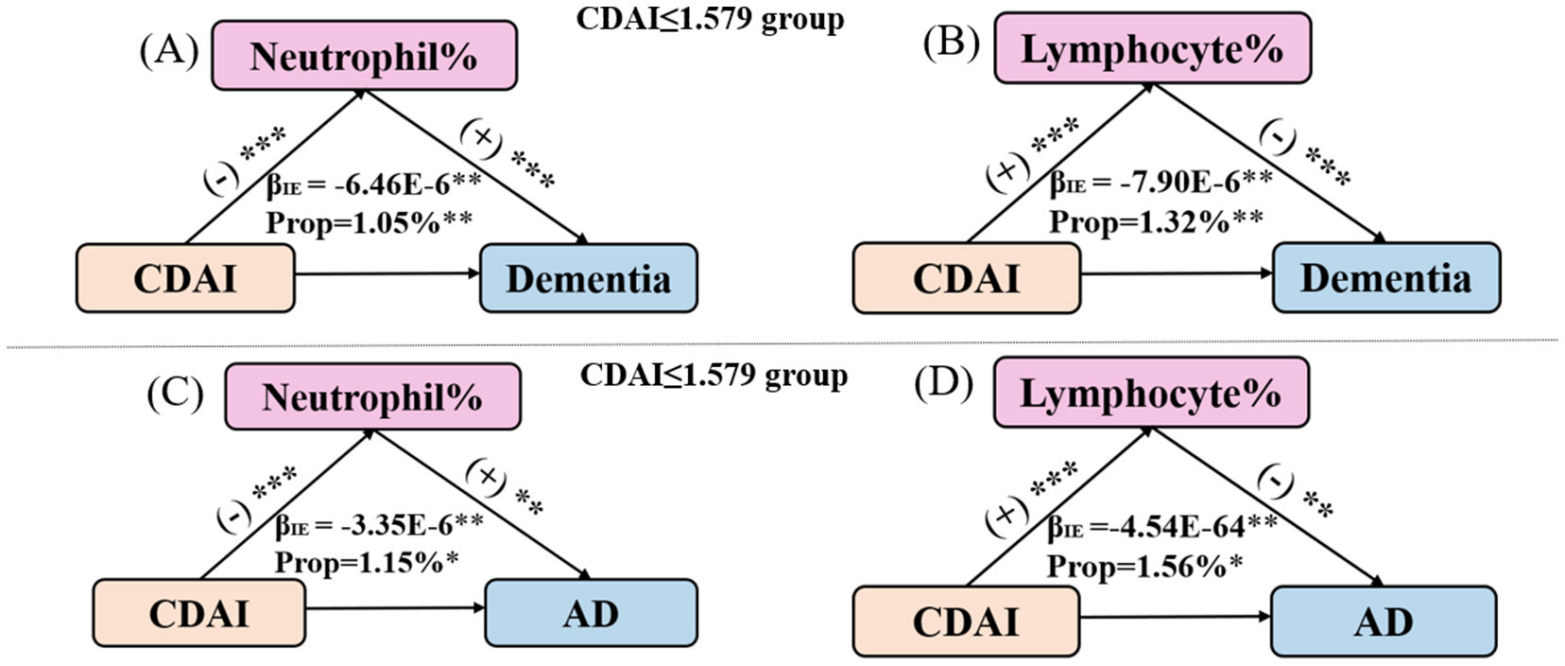
Potential that neutrophil or lymphocyte percentages in the blood mediate relationships between composite dietary antioxidant index (CDAI) and risk of **(A, B)** all-cause dementia or **(C, D)** Alzheimer's dementia (AD) in the subgroup of participants with CDAI below 1.579, using the Model 3 described in Table 2. Other symbols and abbreviations are defined in Figure 3.

Blood inflammatory markers data were available in 152,700 (96.80%) participants. Levels of blood inflammatory markers were significantly higher among those who developed all-cause dementia during follow-up than among those who did not (Supplementary Table S23). CDAI was significantly associated with levels of 11 inflammatory markers (Supplementary Table S25). Among these, levels of 6 markers were associated with risk of all-cause dementia and levels of 3 markers were associated with risk of Alzheimer's dementia (Supplementary Table S26). In exploratory pathway analyses, neutrophil and lymphocyte percentages, neutrophil-to-lymphocyte and platelet-to-lymphocyte ratios, and the SII statistically accounted for small proportions of the association between CDAI and all-cause dementia, with estimated proportions ranging from 0.83% to 2.41% (Supplementary Table S27, Figure 3). Among participants with CDAI below the inflection point of 1.579, the neutrophil percentage accounted for 1.05% of the association between CDAI and all-cause dementia (*P* = 0.002) and 1.15% of the association between CDAI and Alzheimer's dementia (*P* = 0.018). The lymphocyte percentage accounted for 1.32% of the association between CDAI and all-cause dementia (*P* = 0.004) and 1.56% of the association with Alzheimer's dementia (*P* = 0.020) (Supplementary Tables S28–S30, Figure 4). None of the inflammatory markers statistically accounted for the association between CDAI and vascular dementia risk.

### 3.5 Exploratory pathway analysis of gray matter volumes

**Figure 5:**
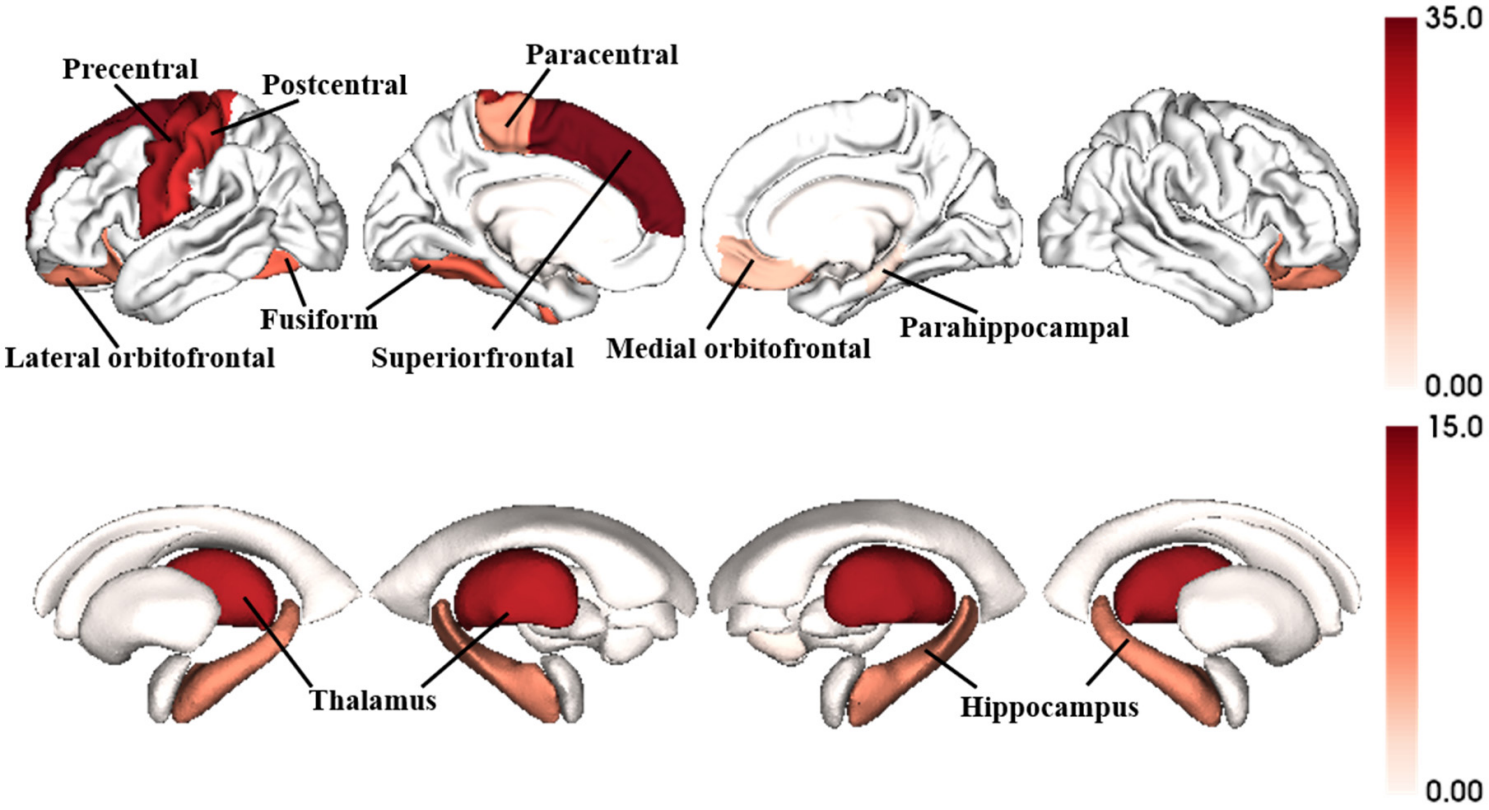
Associations between composite dietary antioxidant index (CDAI) and cortical/subcortical gray matter volumes at enrollment of participants with CDAI below 1.579. Associations were quantified using a multilinear regression model that adjusted for the same variables as Model 3 in Table 2, and strengths of association after Bonferroni correction are shown on a colorscale.

Brain imaging data were available in 22,563 (14.30%) participants (Supplementary Table S33). Across all these participants, CDAI was not significantly associated with gray matter volumes in cortical or subcortical regions (Supplementary Tables S36, S37). When sub-grouped to those with CDAI lower than the inflection point 1.579, multivariate linear regression revealed a positive association between CDAI and gray matter volume in the limbic and frontoparietal regions, including the left fusiform gyrus, paracentral gyrus, postcentral gyrus, precentral gyrus, and superior frontal gyrus; the right medial orbitofrontal gyrus, parahippocampal gyrus, and nucleus accumbens; and the bilateral orbitofrontal gyrus, hippocampus, and thalamus (Supplementary Tables S38, 39, Figure 5). There were no statistically significant indirect associations of CDAI with dementia outcomes via gray matter volumes in the imaging subset (Supplementary Table S43).

## 4 Discussion

In this large-scale prospective cohort study of 157,742 dementia-free participants from the UK Biobank, we systematically investigated the association between the CDAI and dementia incidence. CDAI exhibited a significant inverse association with risks of all-cause dementia and Alzheimer's dementia, particularly in females. Their relationships were characterized by an L-shaped nonlinear dose-response curve, with inflection points identified at a CDAI value of 1.579 for all-cause dementia and 0.848 for Alzheimer's dementia. In addition, these relationships may be partly accounted for by blood inflammatory markers. Our findings indicate that the CDAI may be a useful measure for evaluating the association between dietary antioxidant intake and the risk of dementia and suggest that inflammatory processes may play a role in this association.

CDAI has been reported to be inversely associated with cognitive performance (18), risk of cognitive impairment later in life (19), and biological aging (35), a process closely tied to neurodegeneration. Dietary antioxidant capacity has also been associated with other age-related degenerative conditions, further supporting its relevance to age-related health (36, 37). Our study extends these findings by establishing a longitudinal relationship between higher CDAI and reduced risk of all-cause dementia and Alzheimer's dementia. Higher CDAI indicates greater dietary intake of antioxidants, which may be related to brain health through reduced oxidative stress (7, 38). On the other hand, higher CDAI has also been linked to lower risk of hypertension (15), diabetes (14) and stroke (39), all of which are modifiable risk factors for dementia. We propose that the antioxidant intake captured by CDAI may be associated with dementia risk directly by mitigating oxidative stress-driven neuronal damage and indirectly by reducing the burden of metabolic and cerebrovascular comorbidities that accelerate cognitive decline. Further studies are needed to validate these two pathways and to determine whether antioxidant-rich dietary patterns are associated with subsequent dementia risk in different populations.

Such work should be sure to quantify antioxidant intake comprehensively, as studies on the relationship between dementia risk and the intake of single antioxidants, such as vitamin E (10, 40) and vitamin C (7, 41), have yielded contradictory results. CDAI integrates data on six dietary antioxidants and was calculated based on self-reported usual dietary intake in this study, which may better reflect their long-term synergistic interactions and complex relationship with cognitive health. Another useful comprehensive index may be the “total antioxidant capacity”, which integrates intake of eight antioxidant vitamins and has shown predictive value for predicting cognitive impairment (42). In contrast, the “ferric-reducing antioxidant power” score has failed to show a robust association with risk of dementia or cognitive decline (43, 44), perhaps because it may better reflect only short-term, not long-term, dietary patterns (45).

Of note, the relationship between CDAI and risk of dementia showed an L-shape pattern in our study: risk decreased significantly with increasing CDAI until an inflection point, beyond which risk did not decrease. Similar relationships have been reported between CDAI and risk of coronary heart disease (17), between CDAI and cognitive function related to memory and language (18), and between dietary intake of vitamin E and risk of age-related cognitive impairment (46). In line with this pattern, the potential ability of vitamins C and E or selenium to reduce the risk of Alzheimer's dementia appears to have a limit (10, 47). Research on antioxidant-related dietary strategies and dementia risk should consider this “saturation effect”, where antioxidant intake beyond a certain level does not reduce risk of dementia or cognitive decline, arguing for “precision redox” interventions (5, 48). In fact, excessive intake of antioxidants may bring health risks (49, 50). Future studies of dietary strategies may need to focus on defining the beneficial intake range, and our results about inflection points may be an important step in that direction. Aside from a possible saturation effect, the plateau observed at higher CDAI levels should be approached with caution, as the utilization of vitamin and mineral supplements was more prevalent among participants in the highest CDAI quartiles in this study.

Sex-specific associations were observed in our study, with that higher CDAI significantly associated with lower risk of all-cause dementia and Alzheimer's dementia in women but not in men. Consistently, the association between Mediterranean diet adherence and lower dementia risk has been reported to be stronger in women (8). These findings may reflect sex differences in antioxidant metabolism and responses to oxidative stress (51). For example, the decline in estrogen levels in women following menopause renders them more susceptible than men to oxidative stress (52), which might partly explain the stronger association of dietary antioxidant intake with dementia risk in women.

In our cohort, the association of CDAI with dementia incidence did not vary significantly with age, BMI or APOEε4 status. Nevertheless, the inverse association between CDAI and risk of Alzheimer's dementia appeared stronger among individuals aged at least 60 years and individuals with BMI at least 25 kg/m^2^. These exploratory subgroup patterns match those in which CDAI showed a particularly strong inverse association with risk of depression in a previous study (16), and they are consistent with the generally higher risk of dementia among overweight or obese individuals in midlife (53). The higher risk in these subgroups may reflect the associations of aging and obesity with reduced efficiency of endogenous antioxidant systems, which may translate to greater susceptibility to oxidative damage (54).

Our exploratory pathway analyses suggest that the association between greater antioxidant intake, as captured by higher CDAI, and lower dementia risk may partly involve systemic inflammation, consistent with other large studies (55). Specifically, higher neutrophil abundance and lower lymphocyte abundance statistically accounted for small proportions of the associations between CDAI and all-cause dementia as well as Alzheimer's dementia. These findings are consistent with the idea that neutrophils can induce pathogenesis linked to dementia (56, 57), while lymphocytes are needed to maintain cytokine production and phagocytosis within healthy limits (55, 58). Oxidative stress may exacerbate the risk of injurious inflammatory responses that contribute to dementia and cognitive decline (59). Dietary antioxidants may be associated with lower risk of such decline by simultaneously neutralizing free radicals and suppressing pro-inflammatory signaling (60).

In the imaging subset, we did not find significant indirect associations of CDAI and dementia risk via cortical or subcortical gray matter volumes, consistent with a report that gray matter volumes did not mediate the association between a Mediterranean diet and cognitive performance (61). However, this null finding should be interpreted cautiously, given the limited number of dementia cases in the imaging subset and potential selection into the UK Biobank imaging cohort. In contrast, another study implicated gray matter volume in the parahippocampal gyrus in the association between levels of the antioxidant lutein and crystallized intelligence (62). The discrepancy across these studies may reflect differences in the antioxidant variables and outcomes that they examined. Additionally, we did observe direct correlations between CDAI and gray matter volume in the limbic system and frontoparietal network. The same two brain regions, in a previous study, showed larger gray matter volumes among those consuming a Mediterranean diet rich in antioxidants (21, 63). Conversely, gray matter volume in the frontal cortex was smaller among those consuming a diet higher in cholesterol and lower in antioxidants (64). Future studies incorporating larger cohorts and neuroimaging quite sensitive to early neurodegeneration, such as functional MRI, should continue to explore potential pathways and mechanisms linking dietary antioxidants to brain health and dementia risk.

Our findings may have practical relevance to the public health understanding of dietary antioxidant exposure in relation to dementia risk. A higher CDAI is unlikely to be achieved through isolated intake of a single antioxidant nutrient; instead, it may reflect greater adherence to antioxidant-rich dietary patterns, such as Mediterranean or MIND-like diets (65, 66). These diets are characterized by higher consumption of natural whole foods, including leafy vegetables, berries, nuts, whole grains and olive oil, which contain antioxidant micronutrients such as vitamins A, C and E, selenium and zinc in a complex food matrix. Previous studies have linked Mediterranean dietary patterns to broader health benefits in older adults, including lower risks of dementia (8) and better functional outcomes in several non-neurological conditions (67–69). Importantly, our findings should not suggest that high-dose isolated antioxidants are effective in mitigating dementia risk, as single-nutrient supplements may not emulate the possible advantages associated with antioxidant-rich dietary patterns derived from natural foods (10). Therefore, our findings support further investigation of diverse, plant-rich, whole-food dietary patterns as a potentially modifiable dietary factor related to cognitive resilience in older adults.

### 4.1 Strengths and limitations of the study

The key strengths of our study are the large, community-based sample; prospective cohort design; long follow-up; and comprehensive data on potential confounders. As a result, our findings provide evidence that CDAI may be useful for assessing dietary antioxidant exposure in relation to dementia risk and for informing future studies of antioxidant-rich dietary patterns. Despite the strengths of this study, several limitations should be considered when interpreting the findings. First, the 24-h dietary assessments may not fully reflect long-term habitual dietary intake. To minimize measurement error, we excluded assessments reported to be atypical of usual intake and averaged repeated measurements when available, but residual within-person variability may remain. In addition, because dietary intake was assessed during the baseline assessment period, we were unable to determine whether changes in diet during follow-up influenced dementia risk. Second, dementia ascertainment was based on hospital inpatient records and death registries and did not include primary care or specialist diagnoses (30, 70); some incident dementia cases may have been missed. Such underascertainment could attenuate the observed associations if non-differential with respect to CDAI, whereas differential diagnosis or recording across participant groups could bias the results in either direction. Residual outcome misclassification cannot be completely ruled out, despite adjustment for a range of sociodemographic, lifestyle, and clinical covariates. Third, caution should be taken in interpreting the exploratory pathway analyses. The estimated proportions were small, inflammatory biomarker measures were assessed at one time point, and the temporal sequence between CDAI, potential mediators, and dementia onset could not be fully established. Thus, these results should be considered as hypothesis generating rather than evidence of definitive biological mediation or causal mechanisms. The relatively small imaging subset limited the statistical power of the brain imaging pathway analyses, and future studies with larger imaging cohorts are warranted to further evaluate these findings. Fourth, the exploratory subgroup and interaction findings, especially the stronger association among women, should be interpreted cautiously because no formal correction for multiple testing was applied. Future studies are needed to confirm these subgroup findings in independent populations. Fifth, data were not collected on the dosage and frequency of vitamin or mineral supplementation, so we had to treat such supplementation as a binary variable. This may have led to residual confounding related to supplement use. Finally, our results from the predominantly Caucasian population in the UK Biobank may not necessarily be extrapolated to other ethnicities in other geographic areas with different dietary habits.

## 5 Conclusions

This study provides the first evidence from a large prospective cohort that CDAI is non-linearly associated with risk of all-cause dementia and Alzheimer's dementia in the general population, with evidence of effect modification by sex. The L-shaped association may highlight the importance of moderate and adequate intake of dietary antioxidants. Moderate consumption of antioxidants was linked to a reduced risk of dementia, with preliminary research indicating inflammation could be involved, but further study is needed.

## Data Availability

The data used in this study are available from the UK Biobank (https://www.ukbiobank.ac.uk/), a large-scale biomedical database resource with controlled access. Accessing confidential data requires approval from the UK Biobank Institutional Data Access Committee. Data are not publicly available and can be accessed upon approved application. This study was conducted under UK Biobank application number 98130.
