# Supplementary material for: Nonlinear L-shaped association between composite dietary antioxidant index and risk of dementia: a prospective cohort study
Source: Front Public Health. 2026 Jul 3;14:1839421. doi: 10.3389/fpubh.2026.1839421 (PMC13378127; doi:10.3389/fpubh.2026.1839421)
Supplement: Supplementary file 1 [file Table_1.docx]

**Nonlinear L-shaped association between composite dietary antioxidant index and risk of dementia: A prospective cohort study**

Caimei Luo ^1, #^, Ruihan Wang ^1, #^, Feng Yang ^1^, Linyuan Qin ^1^, Yimeng Ren ^1^, Mengyao Guo^1^, Hanlin Cai ^1^, Shiyu Feng ^1^, Nannan Li ^1^, Hui Gao, Yingying Tang ^1,^ *, Qin Chen ^1,^ *

^#^These authors contributed equally.

^1^ Department of Neurology, West China Hospital of Sichuan University, Chengdu 610041, China

**Supplementary materials**

[Figure S1. Flow chart of study participants 4](#_Toc233317472)

[Table S1: International Classification of Disease codes used to ascertain dementia 5](#_Toc233317473)

[Table S2 Distribution of the number of completed 24-h dietary assessments across CDAI quartiles. 5](#_Toc233317474)

[Table S3 Comparison of baseline characteristics between participants included in the final analysis and those excluded due to missing baseline covariate data 6](#_Toc233317475)

[Table S4 demographic and clinical characteristics of participants based on CDAI quartiles 7](#_Toc233317476)

[Table S5 The association between CDAI and dementia outcomes in physical activity restricted models ^1^ 8](#_Toc233317477)

[Table S6 The associations between CDAI and dementia outcomes in energy intake restricted models ^1^ 9](#_Toc233317478)

[Table S7 The associations between CDAI and dementia outcomes after excluding participates with dementia diagnose in the first five years of follow-up^1^ 10](#_Toc233317479)

[Table S8 The associations between CDAI and dementia outcomes when reincluding participants with missing data on APOEε4 ^1^ 11](#_Toc233317480)

[Table S9 The associations between CDAI and dementia outcomes in the competing risk model with death as the endpoint 12](#_Toc233317481)

[Table S10 The association between CDAI and dementia outcomes using dietary data collected at the baseline assessment visit (cycle 0)^1^ 13](#_Toc233317482)

[Table S11 The association between CDAI and dementia outcomes using a landmark approach with follow-up starting on June 15, 2012^1^ 14](#_Toc233317483)

[Table S12 Association between CDAI and dementia risk after additional adjustment for the number of completed dietary assessments^1^ 15](#_Toc233317484)

[Table S13 The associations between CDAI and dementia outcomes using multiple imputation(N=192264)^1^ 16](#_Toc233317485)

[Table S14 The associations between CDAI and dementia outcomes in sex restricted models (Female) ^1^ 17](#_Toc233317486)

[Table S15 The associations between CDAI and dementia outcomes in sex restricted models (Male) ^1^ 18](#_Toc233317487)

[Table S16 The associations between CDAI and dementia outcomes in age restricted models (＜60 years) ^1^ 19](#_Toc233317488)

[Table S17 The associations between CDAI and dementia outcomes in age restricted models (≥60 years) ^1^ 20](#_Toc233317489)

[Table S18 The associations between CDAI and dementia outcomes in APOEε4 restricted models (APOEε4 carrier) ^1^ 21](#_Toc233317490)

[Table S19 The associations between CDAI and dementia outcomes in APOEε4 restricted models (APOEε4 non-carrier) ^1^ 22](#_Toc233317491)

[Table S20 The associations between CDAI and dementia outcomes in BMI restricted models ((BMI＜25kg/m^2^) ^1^ 23](#_Toc233317492)

[Table S21The associations between CDAI and dementia outcomes in BMI restricted models ((BMI≥25kg/m^2^) ^1^ 24](#_Toc233317493)

[Table S22 Modification for the association between CDAI and risk of incident dementia. 25](#_Toc233317494)

[Table S23 Comparison of blood inflammatory profiles between dementia cases and non-dementia cases. 26](#_Toc233317495)

[Table S24 Baseline demographic and clinical characteristics of 152700 participants in the inflammatory profiles analyses 27](#_Toc233317496)

[Table S25 Multivariate linear regression of CDAI and blood inflammatory profiles in the overall participants (n=152700) 28](#_Toc233317497)

[Table S26 Longitudinal associations between the risk of incident dementia with blood inflammatory profiles in the overall participants (n=152700) 29](#_Toc233317498)

[Table S27 The mediation effects of blood inflammatory profiles in the association between CDAI and incident dementia risk in the overall participants (n=152700) 30](#_Toc233317499)

[Table S28 Multivariate linear regression of CDAI and blood inflammatory profiles in the lower CDAI participants (CDAI<=1.579, n=107460) 31](#_Toc233317500)

[Table S29 Associations between dementia with blood inflammatory profiles in the lower CDAI participants (CDAI<=1.579, n=107460) 32](#_Toc233317501)

[Table S30 The mediation effects of blood inflammatory profiles in the association between CDAI and incident dementia risk in the lower CDAI participants (CDAI<=1.579, n=107460) 33](#_Toc233317502)

[Table S31 Multivariate linear regression of CDAI and blood inflammatory profiles in the higher CDAI participants (CDAI>1.579, n=45240) 34](#_Toc233317503)

[Table S32 Associations between blood inflammatory profiles and dementia in the higher CDAI participants (CDAI>1.579, n=45240) ^1^ 35](#_Toc233317504)

[Table S33 Comparison of cortical gray matter volumes between dementia cases and non-dementia cases. 36](#_Toc233317505)

[Table S34 Comparison of subcortical gray matter volumes between dementia cases and non-dementia cases. 38](#_Toc233317506)

[Table S35 Baseline demographic and clinical of 22563 participants in brain structure analyses 40](#_Toc233317507)

[Table S36 Correlation between CDAI and volume of cortical regions in the overall participants (n=22563) 41](#_Toc233317508)

[Table S37 Correlation between CDAI and volume of subcortical regions in the overall participants(n=22563) 43](#_Toc233317509)

[Table S38 Correlation between CDAI and volume of cortical regions in the lower CDAI group (CDAI≤1.579, n=15687) 45](#_Toc233317510)

[Table S39 Correlation between CDAI and volume of subcortical regions in the lower group (CDAI≤1.579, n=15687) 47](#_Toc233317511)

[Table S40 Correlation between CDAI and volume of cortical regions in the higher group (CDAI＞1.579, n=6876) 49](#_Toc233317512)

[Table S41 Correlation between CDAI and volume of subcortical regions in the higher group (CDAI＞1.579, n=6876) 51](#_Toc233317513)

[Table S42 Longitudinal associations between gray matter volume and the risk of dementia in the lower group (CDAI≤1.579, n=15687) 53](#_Toc233317514)

[Table S43The mediation effects of gray matter volume in the association between CDAI and incident dementia risk in the lower group (CDAI<=1.579, n=15687) 54](#_Toc233317515)

**
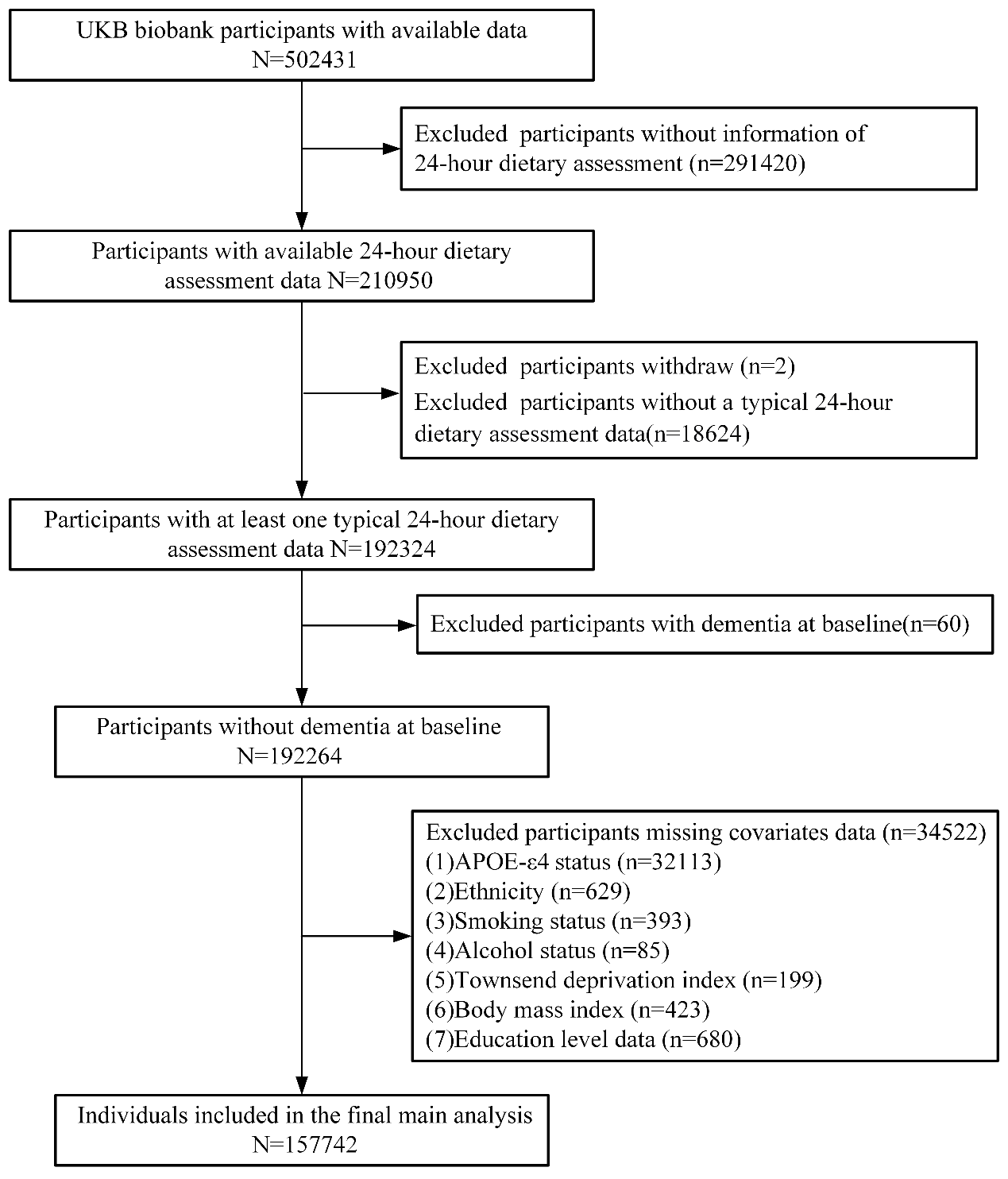
**

**Figure S1. Flow chart of study participants**

**Table S1: International Classification of Disease** **codes used to ascertain dementia**

| Diagnosis | ICD-10 codes |
| --- | --- |
| All-cause dementia | A81.0, F00, F00.0, F00.1, F00.2, F00.9, F01, F01.0, F01.1, F01.2, F01.3, F01.8, F01.9, F02, F02.0, F02.1, F02.2, F02.3, F02.4, F02.8, F03, F05.1, F10.6, G30, G30.0, G30.1, G30.8, G30.9, G31.0, G31.1, G31.8, I67.3. |
| Alzheimer's dementia | F00, F00.0, F00.1, F00.2, F00.9, G30, G30.0, G30.1, G30.8, G30.9. |
| Vascular dementia | F01, F01.0, F01.1, F01.2, F01.3, F01.8, F01.9, I67.3. |

ICD, International Classification of Disease

**Table S2 Distribution of the number of completed 24-h dietary assessments across CDAI quartiles.**

|  | **Overall** | **Quartile 1** | **Quartile 2** | **Quartile 3** | **Quartile 4** | **p value** |
| --- | --- | --- | --- | --- | --- | --- |
| **Number of dietary assessments** |  |  |  |  |  | <0.001 |
| 1 | 72183 (45.76%) | 21876 (55.47%) | 16134 (40.91%) | 15430 (39.13%) | 18743 (47.53%) |  |
| 2 | 41500 (26.31%) | 9362 (23.74%) | 10993 (27.88%) | 10960 (27.79%) | 10185 (25.83%) |  |
| 3 | 28675 (18.18%) | 5555 (14.09%) | 7950 (20.16%) | 8268 (20.97%) | 6902 (17.50%) |  |
| 4 | 13479 (8.54%) | 2346 (5.95%) | 3809 (9.66%) | 4205 (10.66%) | 3119 (7.91%) |  |
| 5 | 1905 (1.21%) | 297 (0.75%) | 549 (1.39%) | 572 (1.45%) | 487 (1.23%) |  |

**Table S3 Comparison of baseline characteristics between participants included in the final analysis and those excluded due to missing baseline covariate data**

| **Characteristic** | **Overall** | **Included participants** | **Excluded participants** | **statistical value** | **p value** |
| --- | --- | --- | --- | --- | --- |
| N | 210950 | 157742 | 53218 |  |  |
| **Age, years** | 57.00 (50.00-63.00) | 57.00 (50.00-63.00) | 57.00 (49.00-62.00) | 34.335 | <0.001 |
| **^1^Education, years** | 15.00 (10.00-20.00) | 15.00 (10.00-20.00) | 15.00 (10.00-20.00) | 254.354 | <0.001 |
| **^2^TDI** | -2.31 (-3.72-0.08) | -2.36 (-3.75--0.02) | -2.16 (-3.66-0.37) | 188.919 | <0.001 |
| **^3^BMI, kgm^−2^** | 26.26 (23.76-29.34) | 26.15 (23.67-29.19) | 26.61 (24.03-29.78) | 444.475 | <0.001 |
| **Female, no. (%)** | 116204 (55.09) | 87,319 (55.36) | 28887 (54.29) | 18.429 | <0.001 |
| **Race, no. (%)** |  |  |  | 2314.954 | <0.001 |
| White | 191701 (90.87) | 143,722 (91.11) | 47988 (90.17) |  |  |
| Mixed | 6695 (3.17) | 4,965 (3.15) | 1730 (3.25) |  |  |
| Asian | 8770 (4.16) | 6,592 (4.18) | 2179 (4.09) |  |  |
| Other | 3155 (1.50) | 2,463 (1.56) | 692 (1.30) |  |  |
| Missing | 629 (0.37) | 0 (0.00) | 629 (1.18) |  |  |
| **Cancer, no. (%)** | 15965 (7.57) | 11,833 (7.50) | 4132 (7.77) | 3.943 | 0.047 |
| **APOE-ε4 carrier, no. (%)** |  |  |  | 125047.799 | <0.001 |
| Non-carrier | 129317 (61.30) | 113,320 (71.84) | 16004 (30.07) |  |  |
| Carrier | 49520 (23.47) | 444,422 (28.16) | 5101 (9.59) |  |  |
| Missing | 32113 (16.67) | 0 (0.00) | 32113 (60.34) |  |  |
| **Smoking, no. (%)** |  |  |  | 2203.722 | <0.001 |
| Never | 118982 (56.40) | 89,953 (57.03) | 29035 (54.55) |  |  |
| Previous | 74856 (35.49) | 56,608 (35.89) | 18252 (34.30) |  |  |
| Current | 16546 (7.84) | 11,181 (7.09) | 5365 (10.08) |  |  |
| Missing | 566 (0.27) | 0 (0.00) | 566 (1.06) |  |  |
| **Alcohol, no. (%)** |  |  |  | 628.712 | <0.001 |
| Never | 6870 (3.26) | 5,111 (3.24) | 1759 (3.31) |  |  |
| Previous | 6458 (3.06) | 4,722 (2.99) | 1736 (3.26) |  |  |
| Current | 197414 (93.58) | 147,909 (93.77) | 49515 (93.04) |  |  |
| Missing | 208 (0.10) | 0 (0.00) | 208 (0.39) |  |  |
| **Diabetes, no. (%)** | 4886 (2.32) | 3,444 (2.18) | 1442 (2.71) | 48.747 | <0.001 |
| **Hypertension, no. (%)** | 38141 (18.08) | 28,023 (17.77) | 10119 (19.02) | 42.043 | <0.001 |
| **Hyperlipemia, no. (%)** | 5927 (2.81) | 4,238 (2.69) | 1689 (3.17) | 34.599 | <0.001 |
| **CVD, no. (%)** | 7148 (3.39) | 5,174 (3.28) | 1975 (3.71) | 22.682 | <0.001 |
| **Stroke, no. (%)** | 704 (0.33) | 533 (0.34) | 171 (0.32) | 0.328 | 0.567 |
| **Vitamin and mineral**  **supplements, no. (%)** | 68807 (32.62) | 51,585 (32.70) | 17224 (32.37) | 2.018 | 0.155 |

Sample sizes varied across variables because of missing data: ¹education, n = 210,103; ²TDI, n = 210,711; and ³BMI, n = 210,417.

**Table S4 demographic and clinical characteristics of participants based on CDAI quartiles**

| **Characteristic** | **Q1(<-2.545)** | **Q2(≥ -2.545, < -0.349)** | **Q3(≥ - 0.349, < 2.132)** | **Q4(≥ 2.132)** | **p value** |
| --- | --- | --- | --- | --- | --- |
| **N** | **39436** | **39435** | **39435** | **39436** |  |
| **Age, years** | 56.00 (49.0-62.0) | 57.00 (50.0-63.0) | 58.00 (50.0-63.0) | 58.00 (50.0-63.0) | <0.001 |
| **Female, no. (%)** | 23256 (58.97%) | 23120 (58.63%) | 21760 (55.18%) | 19183 (48.64%) | <0.001 |
| **Race, no. (%)** |  |  |  |  | <0.001 |
| White | 35648 (90.39%) | 36159 (91.69%) | 36279 (92.00%) | 35636 (90.36%) |  |
| Mixed | 1429 (3.62%) | 1157 (2.93%) | 1127 (2.86%) | 1252 (3.17%) |  |
| Asian | 1600 (4.06%) | 1605 (4.07%) | 1498 (3.80%) | 1889 (4.79%) |  |
| Other | 759 (1.92%) | 514 (1.30%) | 531 (1.35%) | 659 (1.67%) |  |
| **Education, years** | 13.00 (10.0-20.0) | 15.00 (10.0-20.0) | 19.00 (10.0-20.00) | 19.00 (10.0-20.0) | <0.001 |
| **TDI** | -2.16 (-3.66-0.40) | -2.42 (-3.79--0.17) | -2.47 (-3.81--0.28) | -2.35 (-3.74-0.01) | <0.001 |
| **BMI, kgm^−2^** | 26.54 (23.93-29.67) | 26.10 (23.67-29.10) | 25.92 (23.53-28.85) | 26.04 (23.56-29.10) | <0.001 |
| **APOE-ε4 carrier, no. (%)** |  |  |  |  | 0.071 |
| Non-carrier | 28446 (72.13%) | 28386 (71.98%) | 28405 (72.03%) | 28083 (71.21%) |  |
| Carrier | 10990 (27.87%) | 11049 (28.02%) | 11030(27.97%) | 11353 (28.79%) |  |
| **Smoking, no. (%)** |  |  |  |  | <0.001 |
| Never | 21870 (55.46%) | 22657 (57.45%) | 22864 (57.98%) | 22562 (57.21%) |  |
| Previous | 13955 (35.39%) | 14076 (35.69%) | 14235 (36.10%) | 14342 (36.37%) |  |
| Current | 3611 (9.16%) | 2702 (6.85%) | 2336 (5.92%) | 2532 (6.42%) |  |
| **Alcohol, no. (%)** |  |  |  |  | <0.001 |
| Never | 1609 (4.08%) | 1182 (3.00%) | 1081 (2.74%) | 1239 (3.14%) |  |
| Previous | 1321 (3.35%) | 1086 (2.75%) | 1042 (2.64%) | 1273 (3.23%) |  |
| Current | 36506 (92.57%) | 37167 (94.25%) | 37312 (94.62%) | 36924 (93.63%) |  |
| **Diabetes, no. (%)** | 977 (2.48%) | 803 (2.04%) | 766 (1.94%) | 898 (2.28%) | <0.001 |
| **Hypertension, no. (%)** | 7211 (18.29%) | 6786 (17.21%) | 6921 (17.55%) | 7105 (18.02%) | <0.001 |
| **Hyperlipidemia, no. (%)** | 1195 (3.03%) | 987 (2.50%) | 987 (2.50%) | 1069 (2.71%) | <0.001 |
| **CVD, no. (%)** | 1374 (3.48%) | 1247 (3.16%) | 1223 (3.10%) | 1330 (3.37%) | 0.008 |
| **Stroke, no (%)** | 163 (0.41%) | 122 (0.31%) | 113 (0.29%) | 135 (0.34%) | 0.013 |
| **Cancer, no. (%)** | 2881 (7.31%) | 2895 (7.34%) | 2995 (7.59%) | 3062 (7.76%) | 0.045 |
| **Vitamin and mineral**  **supplements, no. (%)** | 12216 (30.98%) | 12585 (31.91%) | 12832 (32.54%) | 13952 (35.38%) | <0.001 |
| **Energy, kcal** | 1533.60 (1296.76-1778.12) | 1866.82 (1653.06-2105.78) | 2112.81 (1873.17-2380.62) | 2504.81 (2184.07-2894.64) | <0.001 |

Continuous variables were presented as mean ± standard deviation or median (IQR), categorical variables were presented as number (percentage). Abbreviations: CDAI, Composite Dietary Antioxidant Index; CVD, cardiovascular disease; TDI, Townsend Deprivation Index; BMI, Body mass index.

**Table S5 The association between CDAI and dementia outcomes in physical activity restricted models ^1^**

|  | **CDAI ^2^** | **No. of participants** | **No. dementia cases** | **Model 1 ^3^** | | **Model 2 ^4^** | | **Model 3 ^5^** | |
| --- | --- | --- | --- | --- | --- | --- | --- | --- | --- |
|  |  |  |  | **HR (95%CI)** | ***P*** | **HR (95%CI)** | ***P*** | **HR (95%CI)** | ***P*** |
|  | Per 1-unit increase | 134112 | 1482 | 0.98(0.96-0.99) | **0.039** | 0.98(0.96-0.99) | **0.038** | 0.98(0.96-0.99) | **0.033** |
|  | Quartile 1 | 33528 | 366 | Ref. |  | Ref. |  | Ref. |  |
| ACD | Quartile 2 | 33528 | 354 | 0.83(0.71-0.96) | **0.015** | 0.84(0.72-0.98) | **0.028** | 0.85(0.73-0.99) | **0.032** |
|  | Quartile 3 | 33528 | 364 | 0.81(0.69-0.95) | **0.010** | 0.83(0.70-0.97) | **0.021** | 0.83(0.71-0.98) | **0.026** |
|  | Quartile 4 | 33528 | 398 | 0.81(0.67-0.98) | **0.032** | 0.82(0.68-0.98) | **0.037** | 0.82(0.67-0.99) | **0.037** |
|  | P for trend | 134112 | 1482 |  | **0.038** |  | **0.047** |  | **0.047** |
|  | Per 1-unit increase | 134112 | 626 | 0.99(0.96-1.03) | 0.836 | 0.99(0.96-1.03) | 0.783 | 0.99(0.96-1.03) | 0.712 |
|  | Quartile 1 | 33528 | 159 | Ref. |  | Ref. |  | Ref. |  |
| AD | Quartile 2 | 33528 | 156 | 0.87(0.69-1.10) | 0.238 | 0.87(0.69-1.10) | 0.247 | 0.87(0.69-1.10) | 0.248 |
|  | Quartile 3 | 33528 | 140 | 0.76(0.59-0.98) | **0.038** | 0.76(0.60-0.98) | **0.041** | 0.77(0.59-0.99) | **0.040** |
|  | Quartile 4 | 33528 | 171 | 0.91(0.68-1.21) | 0.531 | 0.90(0.67-1.21) | 0.490 | 0.89(0.67-1.20) | 0.448 |
|  | P for trend | 134112 | 626 |  | 0.366 |  | 0.340 |  | 0.307 |
|  | Per 1-unit increase | 134112 | 266 | 1.004(0.96-1.05) | 0.862 | 1.003(0.96-1.05) | 0.888 | 1.003(0.96-1.05) | 0.895 |
|  | Quartile 1 | 33528 | 57 | Ref. |  | Ref. |  | Ref. |  |
| VD | Quartile 2 | 33528 | 67 | 1.06(0.73-1.53) | 0.760 | 1.12(0.77-1.62) | 0.549 | 1.12(0.77-1.63) | 0.528 |
|  | Quartile 3 | 33528 | 64 | 0.98(0.66-1.46) | 0.939 | 1.04(0.70-1.55) | 0.828 | 1.05(0.71-1.57) | 0.795 |
|  | Quartile 4 | 33528 | 78 | 1.15(0.73-1.80) | 0.538 | 1.18(0.75-1.86) | 0.469 | 1.18(0.75-1.86) | 0.460 |
|  | P for trend | 134112 | 266 |  | 0.647 |  | 0.581 |  | 0.569 |

**^1^**Estimated effects were calculated using Cox regression model.

^2^ Quartiles were defined as follows: quartile 1, CDAI < -2.545; quartile 2, -2.545 ≤ CDAI < -0.349; quartile 3, -0.349 ≤ CDAI < 2.132; and quartile 4, CDAI ≥ 2.132.

^3^ Model 1 was adjusted for age, sex, ethnicity, education level, APOEε4 status, daily energy intake, and vitamin and mineral supplements.

^4^ Model 2 adjusted for the same variables as well as diabetes, hypertension, hyperlipidemia, body mass index, Townsend deprivation index, alcohol consumption, and smoking status.

^5^ Model 3 adjusted for the same variables as Model 2 in addition to cardiovascular disease, stroke, cancer and physical activity.

CDAI, composite dietary antioxidant index; ACD, all-cause dementia; AD, Alzheimer’s dementia, VD, vascular dementia. HR, hazard ratio; CI, confidence interval.

**Table S6 The associations between CDAI and dementia outcomes in energy intake restricted models ^1^**

|  | **CDAI^2^** | **No. of participants** | **No. dementia cases** | **Model 1^3^** | | **Model 2^4^** | | **Model 3^5^** | |
| --- | --- | --- | --- | --- | --- | --- | --- | --- | --- |
|  |  |  |  | **HR (95%CI)** | ***P*** | **HR (95%CI)** | ***P*** | **HR (95%CI)** | ***P*** |
|  | Per 1-unit increase | 156349 | 1805 | 0.98(0.96-0.99) | **0.034** | 0.98(0.96-0.99) | **0.034** | 0.98(0.96-0.99) | **0.029** |
|  | Quartile 1 | 39088 | 458 | Ref. |  | Ref. |  | Ref. |  |
| ACD | Quartile 2 | 39087 | 428 | 0.81(0.70-0.93) | **0.002** | 0.82(0.72-0.94) | **0.006** | 0.830.72-0.95 | **0.007** |
|  | Quartile 3 | 39087 | 428 | 0.77(0.67-0.90) | **0.001** | 0.79(0.68-0.92) | **0.002** | 0.80(0.69-0.92) | **0.003** |
|  | Quartile 4 | 39087 | 491 | 0.82(0.69-0.97) | **0.021** | 0.82(0.69-0.97) | **0.023** | 0.82(0.69-0.97) | **0.024** |
|  | P for trend | 156349 | 1805 |  | **0.023** |  | **0.026** |  | **0.028** |
|  | Per 1-unit increase | 156349 | 786 | 0.99(0.96-1.01) | 0.356 | 0.98(0.96-1.01) | 0.274 | 0.98(0.96-1.01) | 0.267 |
|  | Quartile 1 | 39088 | 210 | Ref. |  | Ref. |  | Ref. |  |
| AD | Quartile 2 | 39087 | 188 | 0.78(0.64-0.96) | **0.021** | 0.79(0.64-0.97) | **0.024** | 0.79(0.64-0.97) | **0.023** |
|  | Quartile 3 | 39087 | 173 | 0.70(0.56-0.88) | **0.002** | 0.70(0.56-0.88) | **0.003** | 0.70(0.56-0.88) | **0.002** |
|  | Quartile 4 | 39087 | 215 | 0.82(0.63-1.06) | 0.124 | 0.80(0.62-1.04) | 0.099 | 0.80(0.62-1.04) | 0.090 |
|  | P for trend | 156349 | 786 |  | 0.089 |  | 0.072 |  | 0.066 |
|  | Per 1-unit increase | 156349 | 317 | 0.99(0.95-1.04) | 0.805 | 0.99(0.95-1.04) | 0.809 | 0.99(0.95-1.04) | 0.777 |
|  | Quartile 1 | 39088 | 73 | Ref. |  | Ref. |  | Ref. |  |
| VD | Quartile 2 | 39087 | 81 | 1.02(0.73-1.42) | 0.917 | 1.07(0.77-1.50) | 0.662 | 1.08(0.78-1.50) | 0.643 |
|  | Quartile 3 | 39087 | 75 | 0.93(0.65-1.33) | 0.686 | 0.98(0.68-1.41) | 0.934 | 0.98(0.69-1.42) | 0.942 |
|  | Quartile 4 | 39087 | 88 | 1.04(0.69-1.58) | 0.837 | 1.07(0.70-1.62) | 0.753 | 1.07(0.70-1.62) | 0.763 |
|  | P for trend | 156349 | 317 |  | 0.971 |  | 0.894 |  | 0.907 |

**^1^**Estimated effects were calculated using Cox regression model.

^2^ Quartiles were defined as follows: quartile 1, CDAI < -2.545; quartile 2, -2.545 ≤ CDAI < -0.349; quartile 3, -0.349 ≤ CDAI < 2.132; and quartile 4, CDAI ≥ 2.132.

^3^ Model 1 was adjusted for age, sex, ethnicity, education level, APOEε4 status, daily energy intake, and vitamin and mineral supplements.

^4^ Model 2 adjusted for the same variables as well as diabetes, hypertension, hyperlipidemia, body mass index, Townsend deprivation index, alcohol consumption, and smoking status.

^5^ Model 3 adjusted for the same variables as Model 2 in addition to cardiovascular disease, stroke and cancer.

CDAI, composite dietary antioxidant index; ACD, all-cause dementia; AD, Alzheimer’s dementia, VD, vascular dementia. HR, hazard ratio; CI, confidence interval.

Table S7 The associations between CDAI and dementia outcomes after excluding participates with dementia diagnose in the first five years of follow-up^1^

|  | **CDAI** | **No. of participants** | **No. dementia cases** | **Model 1** | | **Model 2** | | **Model 3** | |
| --- | --- | --- | --- | --- | --- | --- | --- | --- | --- |
|  |  |  |  | **HR (95%CI)** | ***P*** | **HR (95%CI)** | ***P*** | **HR (95%CI)** | ***P*** |
|  | Per 1-unit increase | 156312 | 1722 | 0.98(0.96-0.99) | **0.042** | 0.98(0.96-0.99) | **0.045** | 0.98(0.96-0.99) | **0.039** |
|  | Quartile 1 | 39078 | 434 | Ref. |  | Ref. |  | Ref. |  |
| ACD | Quartile 2 | 39078 | 414 | 0.81(0.70-0.93) | **0.003** | 0.83(0.72-0.95) | **0.008** | 0.83(0.72-0.96) | **0.010** |
|  | Quartile 3 | 39078 | 408 | 0.77(0.66-0.90) | **0.001** | 0.79(0.68-0.92) | **0.002** | 0.80(0.68-0.93) | **0.003** |
|  | Quartile 4 | 39078 | 466 | 0.82(0.69-0.98) | 0.025 | 0.82(0.69-0.98) | **0.029** | 0.83(0.70-0.98) | **0.032** |
|  | P for trend | 156312 | 1722 |  | 0.025 |  | **0.029** |  | **0.034** |
|  | Per 1-unit increase | 156312 | 752 | 0.99(0.97-1.02) | 0.651 | 0.99(0.96-1.02) | 0.551 | 0.99(0.96-1.02) | 0.541 |
|  | Quartile 1 | 39078 | 198 | Ref. |  | Ref. |  | Ref. |  |
| AD | Quartile 2 | 39078 | 185 | 0.80(0.65-0.99) | **0.040** | 0.80(0.65-0.99) | **0.047** | 0.81(0.65-0.99) | **0.046** |
|  | Quartile 3 | 39078 | 163 | 0.72(0.57-0.91) | **0.005** | 0.72(0.57-0.91) | **0.006** | 0.72(0.57-0.91) | **0.006** |
|  | Quartile 4 | 39078 | 206 | 0.86(0.66-1.12) | 0.277 | 0.85(0.66-1.11) | 0.239 | 0.85(0.65-1.11) | 0.227 |
|  | P for trend | 156312 | 752 |  | 0.196 |  | 0.169 |  | 0.162 |
|  | Per 1-unit increase | 156312 | 296 | 0.99(0.94-1.04) | 0.670 | 0.99(0.95-1.04) | 0.708 | 0.99(0.95-1.04) | 0.671 |
|  | Quartile 1 | 39078 | 68 | Ref. |  | Ref. |  | Ref. |  |
| VD | Quartile 2 | 39078 | 75 | 0.99(0.70-1.40) | 0.968 | 1.06(0.75-1.49) | 0.747 | 1.06(0.75-1.50) | 0.734 |
|  | Quartile 3 | 39078 | 68 | 0.88(0.60-1.27) | 0.491 | 0.93(0.64-1.36) | 0.738 | 0.94(0.64-1.37) | 0.740 |
|  | Quartile 4 | 39078 | 85 | 1.00(0.65-1.53) | 0.994 | 1.03(0.67-1.58) | 0.888 | 1.03(0.67-1.58) | 0.900 |
|  | P for trend | 156312 | 296 |  | 0.847 |  | 0.941 |  | 0.926 |

**^1^**Estimated effects were calculated using Cox regression model. Models were adjusted, and CDAI quartiles were defined, as described in Table S5.

CDAI, composite dietary antioxidant index; ACD, all-cause dementia; AD, Alzheimer’s dementia, VD, vascular dementia. HR, hazard ratio; CI, confidence interval.

|  | **CDAI** | **No. of participants** | **No. dementia cases** | **Model 1** | | **Model 2** | | **Model 3** | |
| --- | --- | --- | --- | --- | --- | --- | --- | --- | --- |
|  |  |  |  | **HR (95%CI)** | ***P*** | **HR (95%CI)** | ***P*** | **HR (95%CI)** | ***P*** |
|  | Per 1-unit increase | 189371 | 2210 | 0.98(0.97-0.99) | **0.028** | 0.98(0.97-0.99) | **0.028** | 0.98(0.97-0.99) | **0.032** |
|  | Quartile 1 | 47343 | 549 | Ref. |  | Ref. |  | Ref. |  |
| ACD | Quartile 2 | 47343 | 539 | 0.83(0.73-0.94) | **0.003** | 0.85(0.75-0.96) | **0.011** | 0.86(0.76-0.97) | **0.015** |
|  | Quartile 3 | 47343 | 517 | 0.78(0.68-0.89) | **<0.001** | 0.80(0.70-0.91) | **0.001** | 0.81(0.71-0.92) | **0.002** |
|  | Quartile 4 | 47343 | 605 | 0.82(0.71-0.96) | **0.014** | 0.83(0.71-0.97) | **0.020** | 0.84(0.72-0.98) | **0.026** |
|  | P for trend | 189371 | 2210 |  | **0.011** |  | **0.016** |  | **0.021** |
|  | Per 1-unit increase | 189371 | 965 | 0.99(0.97-1.02) | 0.517 | 0.99(0.97-1.02) | 0.436 | 0.99(0.97-1.01) | 0.413 |
|  | Quartile 1 | 47343 | 253 | Ref. |  | Ref. |  | Ref. |  |
| AD | Quartile 2 | 47343 | 234 | 0.79(0.66-0.96) | **0.015** | 0.80(0.67-0.97) | **0.021** | 0.80(0.67-0.97) | **0.022** |
|  | Quartile 3 | 47343 | 213 | 0.73(0.60-0.90) | **0.003** | 0.74(0.60-0.90) | **0.003** | 0.74(0.60-0.90) | **0.004** |
|  | Quartile 4 | 47343 | 265 | 0.87(0.69-1.09) | 0.227 | 0.86(0.68-1.08) | 0.197 | 0.86(0.68-1.08) | 0.190 |
|  | P for trend | 189371 | 965 |  | 0.169 |  | 0.147 |  | 0.143 |
|  | Per 1-unit increase | 189371 | 399 | 0.99(0.95-1.03) | 0.681 | 0.99(0.96-1.03) | 0.682 | 0.99(0.96-1.03) | 0.673 |
|  | Quartile 1 | 47343 | 94 | Ref. |  | Ref. |  | Ref. |  |
| VD | Quartile 2 | 47343 | 100 | 0.93(0.69-1.24) | 0.615 | 0.98(0.73-1.31) | 0.895 | 0.98(0.73-1.32) | 0.93 |
|  | Quartile 3 | 47343 | 92 | 0.87(0.64-1.21) | 0.422 | 0.93(0.68-1.28) | 0.671 | 0.94(0.68-1.29) | 0.70 |
|  | Quartile 4 | 47343 | 113 | 0.97(0.68-1.40) | 0.875 | 0.99(0.69-1.43) | 0.981 | 0.99(0.69-1.43) | 0.985 |
|  | P for trend | 189371 | 399 |  | 0.809 |  | 0.911 |  | 0.915 |

**Table S8 The associations between CDAI and dementia outcomes when reincluding participants with missing data on APOEε4 ^1^**

**^1^**Estimated effects were calculated using Cox regression model. Models were adjusted, and CDAI quartiles were defined, as described in Table S6.

CDAI, composite dietary antioxidant index; ACD, all-cause dementia; AD, Alzheimer’s dementia, VD, vascular dementia. HR, hazard ratio; CI, confidence interval.

**Table S9 The associations between CDAI and dementia outcomes in the competing risk model with death as the endpoint**

|  | **CDAI** | **No. of participants** | **ACD** | | **AD** | | **VD** | |
| --- | --- | --- | --- | --- | --- | --- | --- | --- |
|  |  |  | **HR (95%CI)** | ***P*** | **HR (95%CI)** | ***P*** | **HR (95%CI)** | ***P*** |
|  | Per 1-unit increase | 157742 | 0.980(0.962-0.999) | **0.047** | 0.986(0.957-1.016) | 0.370 | 0.993(0.949-1.038) | 0.750 |
|  | Quartile 1 | 39078 | Ref. |  | Ref. |  | Ref. |  |
| CDAI | Quartile 2 | 39078 | 0.847(0.738-0.973) | **0.019** | 0.835(0.679-1.026) | 0.086 | 1.074(0.776-1.487) | 0.670 |
|  | Quartile 3 | 39078 | 0.784(0.676-0.910) | **0.001** | 0.708(0.565-0.887) | **0.003** | 0.897(0.624-1.291) | 0.560 |
|  | Quartile 4 | 39078 | 0.819(0.688-0.973) | **0.023** | 0.847(0.652-1.100) | 0.210 | 1.032(0.680-1.569) | 0.880 |
|  | P for trend | 156312 |  | 0.017 |  | 0.120 |  | 0.870 |

Model was adjusted for the variables in Model 3, as described in Table S6. CDAI quartiles were defined as described in Table S6.

CDAI, composite dietary antioxidant index; ACD, all-cause dementia; AD, Alzheimer’s dementia, VD, vascular dementia. HR, hazard ratio; CI, confidence interval.

**Table S10 The association between CDAI and dementia outcomes using dietary data collected at the baseline assessment visit (****cycle 0)^1^**

|  | **CDAI** | **No. of participants** | **No. dementia cases** | **Model 1** | | **Model 2** | | **Model 3** | |
| --- | --- | --- | --- | --- | --- | --- | --- | --- | --- |
|  |  |  |  | **HR (95%CI)** | ***P*** | **HR (95%CI)** | ***P*** | **HR (95%CI)** | ***P*** |
|  | Per 1-unit increase | 46762 | 564 | 0.96(0.93-0.99) | **0.021** | 0.96(0.93-0.99) | **0.022** | 0.96(0.93-0.99) | **0.024** |
|  | Quartile 1 | 11691 | 133 | Ref. |  | Ref. |  | Ref. |  |
| ACD | Quartile 2 | 11690 | 128 | 0.86(0.68-1.09) | 0.223 | 0.90(0.71-1.41) | 0.385 | 0.91(0.71-1.15) | 0.413 |
|  | Quartile 3 | 11690 | 148 | 0.72(0.55-0.94) | **0.015** | 0.75(0.57-0.98) | **0.034** | 0.76(0.58-0.99) | **0.042** |
|  | Quartile 4 | 11691 | 155 | 0.70(0.51-0.96) | **0.025** | 0.70(0.51-0.96) | **0.027** | 0.71(0.52-0.97) | **0.031** |
|  | P for trend | 46762 | 564 |  | **0.012** |  | **0.014** |  | **0.016** |
|  | Per 1-unit increase | 46762 | 234 | 0.94(0.89-0.99) | **0.023** | 0.94(0.89-0.99) | **0.020** | 0.94(0.89-0.99) | **0.018** |
|  | Quartile 1 | 11691 | 46 | Ref. |  | Ref. |  | Ref. |  |
| AD | Quartile 2 | 11690 | 46 | 0.77(0.54-1.10) | 0.143 | 0.78(0.55-1.11) | 0.167 | 0.78(0.54-1.11) | 0.164 |
|  | Quartile 3 | 11690 | 63 | 0.58(0.38-0.87) | **0.010** | 0.58(0.38-0.88) | **0.011** | 0.58(0.38-0.87) | **0.010** |
|  | Quartile 4 | 11691 | 79 | 0.57(0.35-0.94) | **0.026** | 0.56(0.34-0.91) | **0.019** | 0.56(0.34-0.91) | **0.019** |
|  | P for trend | 46762 | 234 |  | **0.011** |  | **0.008** |  | **0.008** |
|  | Per 1-unit increase | 46762 | 104 | 0.98(0.92-1.06) | 0.722 | 0.99(0.92-1.06) | 0.744 | 0.99(0.92-1.06) | 0.696 |
|  | Quartile 1 | 11691 | 28 | Ref. |  | Ref. |  | Ref. |  |
| VD | Quartile 2 | 11690 | 19 | 0.85(0.49-1.48) | 0.571 | 0.95(0.54-1.64) | 0.841 | 0.94(0.54-1.63) | 0.824 |
|  | Quartile 3 | 11690 | 27 | 0.59(0.31-1.13) | 0.111 | 0.65(0.34-1.24) | 0.192 | 0.94(0.34-1.23) | 0.183 |
|  | Quartile 4 | 11691 | 30 | 0.81(0.40-1.64) | 0.558 | 0.83(0.41-1.69) | 0.615 | 1.03(0.40-1.66) | 0.570 |
|  | P for trend | 46762 | 104 |  | 0.374 |  | 0.418 |  | 0.390 |

**^1^**Estimated effects were calculated using Cox regression model. Models were adjusted, and CDAI quartiles were defined, as described in Table S6.

CDAI, composite dietary antioxidant index; ACD, all-cause dementia; AD, Alzheimer’s dementia, VD, vascular dementia. HR, hazard ratio; CI, confidence interval.

**Table S11 The association between CDAI and dementia outcomes using a landmark approach with follow-up starting on June 15, 2012^1^**

|  | **CDAI** | **No. of participants** | **No. dementia cases** | **Model 1** | | **Model 2** | | **Model 3** | |
| --- | --- | --- | --- | --- | --- | --- | --- | --- | --- |
|  |  |  |  | **HR (95%CI)** | ***P*** | **HR (95%CI)** | ***P*** | **HR (95%CI)** | ***P*** |
|  | Per 1-unit increase | 157278 | 1785 | 0.98(0.96-0.99) | **0.031** | 0.98(0.96-0.99) | **0.027** | 0.98(0.96-0.99) | **0.026** |
|  | Quartile 1 | 39320 | 493 | Ref. |  | Ref. |  | Ref. |  |
| ACD | Quartile 2 | 39319 | 419 | 0.82(0.71-0.94) | 0.004 | 0.83(0.73-0.96) | **0.010** | 0.84(0.73-0.96) | **0.012** |
|  | Quartile 3 | 39319 | 425 | 0.76(0.66-0.89) | **<0.001** | 0.78(0.67-0.90) | **0.001** | 0.78(0.68-0.91) | **0.002** |
|  | Quartile 4 | 39320 | 448 | 0.81(0.69-0.97) | **0.020** | 0.82(0.69-0.97) | **0.021** | 0.82(0.69-0.97) | **0.024** |
|  | P for trend | 157278 | 1785 |  | **0.015** |  | **0.017** |  | **0.020** |
|  | Per 1-unit increase | 157278 | 782 | 0.96(0.96-1.02) | 0.424 | 0.99(0.96-1.01) | 0.350 | 0.98(0.95-1.01) | 0.325 |
|  | Quartile 1 | 39320 | 218 | Ref. |  | Ref. |  | Ref. |  |
| AD | Quartile 2 | 39319 | 172 | 0.79(0.64-0.97) | **0.028** | 0.80(0.65-0.98) | **0.031** | 0.80(0.65-0.98) | **0.031** |
|  | Quartile 3 | 39319 | 185 | 0.71(0.57-0.90) | **0.003** | 0.71(0.57-0.90) | **0.004** | 0.71(0.57-0.90) | **0.004** |
|  | Quartile 4 | 39320 | 207 | 0.85(0.66-1.10) | 0.221 | 0.84(0.65-1.08) | 0.178 | 0.83(0.64-1.08) | 0.170 |
|  | P for trend | 157278 | 782 |  | 0.158 |  | 0.128 |  | 0.123 |
|  | Per 1-unit increase | 157278 | 308 | 0.99(0.95-1.03) | 0.818 | 0.99(0.95-1.03) | 0.795 | 0.99(0.95-1.04) | 0.756 |
|  | Quartile 1 | 39320 | 88 | Ref. |  | Ref. |  | Ref. |  |
| VD | Quartile 2 | 39319 | 71 | 1.02(0.73-1.42) | 0.926 | 1.08(0.77-1.51) | 0.651 | 1.08(0.77-1.52) | 0.644 |
|  | Quartile 3 | 39319 | 79 | 0.87(0.61-1.23) | 0.483 | 0.93(0.65-1.35) | 0.719 | 0.94(0.64-1.35) | 0.717 |
|  | Quartile 4 | 39320 | 70 | 1.01(0.66-1.53) | 0.958 | 1.04(0.68-1.57) | 0.866 | 1.03(0.68-1.56) | 0.893 |
|  | P for trend | 157278 | 308 |  | 0.843 |  | 0.924 |  | 0.894 |

**^1^**Estimated effects were calculated using Cox regression model. Models were adjusted, and CDAI quartiles were defined, as described in Table S6.

CDAI, composite dietary antioxidant index; ACD, all-cause dementia; AD, Alzheimer’s dementia, VD, vascular dementia. HR, hazard ratio; CI, confidence interval.

**Table S12 Association between CDAI and dementia risk after additional adjustment for the number of completed dietary assessments^1^**

|  | **CDAI** | **No. of participants** | **No. dementia cases** | **Model 1** | | **Model 2** | | **Model 3** | |
| --- | --- | --- | --- | --- | --- | --- | --- | --- | --- |
|  |  |  |  | **HR (95%CI)** | ***P*** | **HR (95%CI)** | ***P*** | **HR (95%CI)** | ***P*** |
|  | Per 1-unit increase | 157742 | 1822 | 0.98(0.96-0.99) | **0.031** | 0.98(0.96-0.99) | **0.027** | 0.98(0.96-0.99) | **0.026** |
|  | Quartile 1 | 39436 | 458 | Ref. |  | Ref. |  | Ref. |  |
| ACD | Quartile 2 | 39435 | 434 | 0.82(0.71-0.94) | 0.004 | 0.83(0.73-0.96) | **0.010** | 0.84(0.73-0.96) | **0.012** |
|  | Quartile 3 | 39435 | 427 | 0.76(0.66-0.89) | **<0.001** | 0.78(0.67-0.90) | **0.001** | 0.78(0.68-0.91) | **0.002** |
|  | Quartile 4 | 39436 | 503 | 0.81(0.69-0.97) | **0.020** | 0.82(0.69-0.97) | **0.021** | 0.82(0.69-0.97) | **0.024** |
|  | P for trend | 157742 | 1822 |  | **0.015** |  | **0.017** |  | **0.020** |
|  | Per 1-unit increase | 157742 | 791 | 0.96(0.96-1.02) | 0.424 | 0.99(0.96-1.01) | 0.350 | 0.98(0.95-1.01) | 0.325 |
|  | Quartile 1 | 39436 | 210 | Ref. |  | Ref. |  | Ref. |  |
| AD | Quartile 2 | 39435 | 189 | 0.79(0.64-0.97) | **0.028** | 0.80(0.65-0.98) | **0.031** | 0.80(0.65-0.98) | **0.031** |
|  | Quartile 3 | 39435 | 173 | 0.71(0.57-0.90) | **0.003** | 0.71(0.57-0.90) | **0.004** | 0.71(0.57-0.90) | **0.004** |
|  | Quartile 4 | 39436 | 219 | 0.85(0.66-1.10) | 0.221 | 0.84(0.65-1.08) | 0.178 | 0.83(0.64-1.08) | 0.170 |
|  | P for trend | 157742 | 791 |  | 0.158 |  | 0.128 |  | 0.123 |
|  | Per 1-unit increase | 157742 | 322 | 0.99(0.95-1.03) | 0.818 | 0.99(0.95-1.03) | 0.795 | 0.99(0.95-1.04) | 0.756 |
|  | Quartile 1 | 39436 | 73 | Ref. |  | Ref. |  | Ref. |  |
| VD | Quartile 2 | 39435 | 83 | 1.02(0.73-1.42) | 0.926 | 1.08(0.77-1.51) | 0.651 | 1.08(0.77-1.52) | 0.644 |
|  | Quartile 3 | 39435 | 73 | 0.87(0.61-1.23) | 0.483 | 0.93(0.65-1.35) | 0.719 | 0.94(0.64-1.35) | 0.717 |
|  | Quartile 4 | 39436 | 93 | 1.01(0.66-1.53) | 0.958 | 1.04(0.68-1.57) | 0.866 | 1.03(0.68-1.56) | 0.893 |
|  | P for trend | 157742 | 322 |  | 0.843 |  | 0.924 |  | 0.894 |

^1^Estimated effects were calculated using Cox regression model. Models were adjusted, and CDAI quartiles were defined, as described in Table S6.

CDAI, composite dietary antioxidant index; ACD, all-cause dementia; AD, Alzheimer’s dementia, VD, vascular dementia. HR, hazard ratio; CI, confidence interval.

**Table S13 The associations between CDAI and dementia outcomes using multiple imputation(N=192264)^1^**

|  | **CDAI** | **No. of participants** |  | **ACD** | |  |  | **AD** | |  |  | **VD** | |
| --- | --- | --- | --- | --- | --- | --- | --- | --- | --- | --- | --- | --- | --- |
|  |  |  | **No. dementia ACD cases** | **HR (95%CI)** | ***P*** |  | **No. dementia AD cases** | **HR (95%CI)** | ***P*** |  | **No. dementia VD cases** | **HR (95%CI)** | ***P*** |
|  | Per 1-unit increase | 192264 | 2264 | 0.98 (0.97-0.99) | **0.042** |  | 987 | 0.99(0.97-1.02) | 0.792 |  | 411 | 0.99(0.95-1.04) | 0.865 |
|  | Quartile 1 | 48066 | 563 | Ref. |  |  | 259 | Ref. |  |  | 97 | Ref. |  |
| CDAI | Quartile 2 | 48066 | 549 | 0.87(0.78-0.98) | **0.026** |  | 236 | 0.82(0.69-0.99) | **0.040** |  | 102 | 1.02(0.76-1.36) | 0.915 |
|  | Quartile 3 | 48066 | 530 | 0.80(0.70-0.92) | **0.001** |  | 218 | 0.74(0.61-0.91) | **0.004** |  | 96 | 0.93(0.68-1.28) | 0.659 |
|  | Quartile 4 | 48066 | 622 | 0.84(0.72-0.98) | **0.025** |  | 274 | 0.86(0.69-1.09) | 0.210 |  | 11 | 1.01(0.70-1.59) | 0.971 |
|  | P for trend |  |  |  | **0.016** |  |  |  | 0.139 |  |  |  | 0.682 |

Model was adjusted for the variables in Model 3, as described in Table S6. CDAI quartiles were defined as described in Table S6.

CDAI, composite dietary antioxidant index; ACD, all-cause dementia; AD, Alzheimer’s dementia, VD, vascular dementia. HR, hazard ratio; CI, confidence interval.

**Table S14 The associations between CDAI and dementia outcomes in sex restricted models (Female) ^1^**

|  | **CDAI** | **No. of participants** | **No. dementia cases** | **Model 1** | | **Model 2** | | **Model 3** | |
| --- | --- | --- | --- | --- | --- | --- | --- | --- | --- |
|  |  |  |  | **HR (95%CI)** | ***P*** | **HR (95%CI)** | ***P*** | **HR (95%CI)** | ***P*** |
|  | Per 1-unit increase | 87319 | 861 | 0.95 (0.92-0.98) | **<0.001** | 0.95 (0.93-0.98) | **0.001** | 0.95 (0.93-0.98) | **0.002** |
|  | Quartile 1 | 23256 | 243 | Ref. |  | Ref. |  | Ref. |  |
| ACD | Quartile 2 | 23120 | 228 | 0.78(0.64-0.94) | **0.009** | 0.80(0.66-0.97) | **0.022** | 0.81(0.67-0.98) | **0.027** |
|  | Quartile 3 | 21760 | 191 | 0.64(0.52-0.79) | **<0.001** | 0.66(0.53-0.81) | **<0.001** | 0.67(0.54-0.83) | **<0.001** |
|  | Quartile 4 | 19183 | 199 | 0.63(0.49-0.81) | **<0.001** | 0.64(0.50-0.83) | **0.001** | 0.65(0.50-0.84) | **0.001** |
|  | P for trend | 87319 | 861 |  | **<0.001** |  | **<0.001** |  | **<0.001** |
|  | Per 1-unit increase | 87319 | 394 | 0.94(0.90-0.98) | **0.010** | 0.95(0.90-0.99) | **0.011** | 0.95(0.91-0.99) | **0.014** |
|  | Quartile 1 | 23256 | 125 | Ref. |  | Ref. |  | Ref. |  |
| AD | Quartile 2 | 23120 | 101 | 0.69(0.52-0.90) | **0.008** | 0.70(0.53-0.93) | **0.014** | 0.71(0.54-0.93) | **0.015** |
|  | Quartile 3 | 21760 | 81 | 0.55(0.40-0.75) | **<0.001** | 0.56(0.41-0.77) | **<0.001** | 0.57(0.41-0.77) | **<0.001** |
|  | Quartile 4 | 19183 | 87 | 0.57(0.41-0.83) | **0.003** | 0.57(0.41-0.83) | **0.004** | 0.58(0.40-0.84) | **0.004** |
|  | P for trend | 87319 | 394 |  | **0.001** |  | **0.002** |  | **0.002** |
|  | Per 1-unit increase | 87319 | 122 | 0.98(0.91-1.06) | 0.573 | 0.99(0.91-1.06) | 0.706 | 0.99(0.92-1.07) | 0.725 |
|  | Quartile 1 | 23256 | 32 | Ref. |  | Ref. |  | Ref. |  |
| VD | Quartile 2 | 23120 | 37 | 1.03(0.62-1.69) | 0.913 | 1.10(0.67-1.82) | 0.705 | 1.11(0.67-1.84) | 0.679 |
|  | Quartile 3 | 21760 | 25 | 0.70(0.39-1.26) | 0.238 | 0.75(0.41-1.35) | 0.336 | 0.75(0.43-1.36) | 0.346 |
|  | Quartile 4 | 19183 | 28 | 0.78(0.39-1.55) | 0.482 | 0.84(0.42-1.67) | 0.617 | 0.85(0.43-1.70) | 0.641 |
|  | P for trend | 87319 | 122 |  | 0.280 |  | 0.371 |  | 0.386 |

^1^Estimated effects were calculated using Cox regression model. Models were adjusted, and CDAI quartiles were defined, as described in Table S6.

CDAI, composite dietary antioxidant index; ACD, all-cause dementia; AD, Alzheimer’s dementia, VD, vascular dementia. HR, hazard ratio; CI, confidence interval.

|  | **CDAI** | **No. of participants** | **No. dementia cases** | **Model 1** | | **Model 2** | | **Model 3** | |
| --- | --- | --- | --- | --- | --- | --- | --- | --- | --- |
|  |  |  |  | **HR (95%CI)** | ***P*** | **HR (95%CI)** | ***P*** | **HR (95%CI)** | ***P*** |
|  | Per 1-unit increase | 70423 | 961 | 0.99 (0.98, 1.02) | 0.926 | 0.99 (0.98, 1.02) | 0.774 | 0.99 (0.98, 1.02) | 0.749 |
|  | Quartile 1 | 16180 | 215 | Ref. |  | Ref. |  | Ref. |  |
| ACD | Quartile 2 | 16315 | 206 | 0.83(0.68-1.02) | 0.071 | 0.85(0.69-1.03) | 0.103 | 0.85(0.70-1.04) | 0.115 |
|  | Quartile 3 | 17675 | 236 | 0.85(0.70-1.06) | 0.163 | 0.88(0.72-1.08) | 0.236 | 0.89(0.72-1.09) | 0.267 |
|  | Quartile 4 | 20254 | 304 | 0.98(0.78-1.23) | 0.877 | 0.97(0.78-1.23) | 0.850 | 0.98(0.77-1.23) | 0.860 |
|  | P for trend | 70423 | 961 |  | 0.943 |  | 0.965 |  | 0.955 |
|  | Per 1-unit increase | 70423 | 397 | 1.02(0.98-1.06) | 0.233 | 1.02(0.98-1.06) | 0.316 | 1.02(0.98-1.06) | 0.365 |
|  | Quartile 1 | 16180 | 85 | Ref. |  | Ref. |  | Ref. |  |
| AD | Quartile 2 | 16315 | 88 | 0.94(0.69-1.27) | 0.668 | 0.93(0.68-1.27) | 0.635 | 0.93(0.68-1.26) | 0.644 |
|  | Quartile 3 | 17675 | 92 | 0.91(0.66-1.26) | 0.583 | 0.90(0.65-1.25) | 0.529 | 0.90(0.65-1.24) | 0.515 |
|  | Quartile 4 | 20254 | 132 | 1.20(0.84-1.73) | 0.299 | 1.17(0.83-1.69) | 0.374 | 1.17(0.81-1.68) | 0.402 |
|  | P for trend | 70423 | 397 |  | 0.306 |  | 0.379 |  | 0.412 |
|  | Per 1-unit increase | 70423 | 200 | 0.99(0.94-1.05) | 0.916 | 0.99(0.94-1.05) | 0.787 | 0.99(0.94-1.05) | 0.746 |
|  | Quartile 1 | 16180 | 41 | Ref. |  | Ref. |  | Ref. |  |
| VD | Quartile 2 | 16315 | 46 | 0.99(0.64-1.53) | 0.963 | 1.04(0.67-1.62) | 0.853 | 1.04(0.67-1.62) | 0.841 |
|  | Quartile 3 | 17675 | 48 | 0.94(0.60-1.49) | 0.804 | 1.00(0.63-1.58) | 0.999 | 1.00(0.63-1.60) | 0.985 |
|  | Quartile 4 | 20254 | 65 | 1.13(0.68-1.89) | 0.638 | 1.13(0.68-1.90) | 0.631 | 1.12(0.67-1.89) | 0.648 |
|  | P for trend | 70423 | 200 |  | 0.963 |  | 0.853 |  | 0.841 |

**Table S15 The associations between CDAI and dementia outcomes in sex restricted models (Male) ^1^**

^1^Estimated effects were calculated using Cox regression model. Models were adjusted, and CDAI quartiles were defined, as described in Table S6.

CDAI, composite dietary antioxidant index; ACD, all-cause dementia; AD, Alzheimer’s dementia, VD, vascular dementia. HR, hazard ratio; CI, confidence interval.

|  | **CDAI** | **No. of participants** | **No. dementia cases** | **Model 1** | | **Model 2** | | **Model 3** | |
| --- | --- | --- | --- | --- | --- | --- | --- | --- | --- |
|  |  |  |  | **HR (95%CI)** | ***P*** | **HR (95%CI)** | ***P*** | **HR (95%CI)** | ***P*** |
|  | Per 1-unit increase | 93234 | 250 | 0.96(0.92-1.10) | 0.214 | 0.97(0.93-1.02) | 0.255 | 0.97(0.92-1.02) | 0.226 |
|  | Quartile 1 | 24774 | 74 | Ref. |  | Ref. |  | Ref. |  |
| ACD | Quartile 2 | 23086 | 60 | 0.80(0.56-1.14) | 0.210 | 0.83(0.59-1.19) | 0.320 | 0.84(0.59-1.20) | 0.345 |
|  | Quartile 3 | 22769 | 42 | 0.52(0.34_0.78) | **0.002** | 0.55(0.36-0.83) | **0.004** | 0.55(0.36-0.82) | **0.005** |
|  | Quartile 4 | 22605 | 74 | 0.82(0.53-1.27) | 0.382 | 0.85(0.55-1.31) | 0.459 | 0.84(0.54-1.31) | 0.444 |
|  | P for trend | 93234 | 250 |  | 0.152 |  | 0.194 |  | 0.182 |
|  | Per 1-unit increase | 93234 | 88 | 0.94(0.86-1.02) | 0.133 | 0.94(0.86-1.02) | 0.135 | 0.94(0.86-1.02) | 0.137 |
|  | Quartile 1 | 24774 | 25 | Ref. |  | Ref. |  | Ref. |  |
| AD | Quartile 2 | 23086 | 25 | 0.99(0.55-1.76) | 0.962 | 1.00(0.56-1.81) | 0.975 | 1.02(0.57-1.82) | 0.952 |
|  | Quartile 3 | 22769 | 13 | 0.48(0.23-0.99) | 0.047 | 0.49(0.24-1.02) | 0.057 | 0.49(0.24-1.03) | 0.059 |
|  | Quartile 4 | 22605 | 25 | 0.83(0.40-1.75) | 0.627 | 0.83(0.39-1.75) | 0.626 | 0.84(0.40-1.77) | 0.639 |
|  | P for trend | 93234 | 88 |  | 0.293 |  | 0.298 |  | 0.304 |
|  | Per 1-unit increase | 93234 | 29 | 1.00(0.87-1.15) | 0.992 | 0.99(0.86-1.14) | 0.933 | 0.98(0.85-1.13) | 0.822 |
|  | Quartile 1 | 24774 | 9 | Ref. |  | Ref. |  | Ref. |  |
| VD | Quartile 2 | 23086 | 4 | 0.43(0.13-1.45) | 0.175 | 0.47(0.14-1.57) | 0.218 | 0.46(0.14-1.55) | 0.213 |
|  | Quartile 3 | 22769 | 6 | 0.59(0.19-1.85) | 0.369 | 0.64(0.21-2.00) | 0.445 | 0.61(0.20-1.89) | 0.388 |
|  | Quartile 4 | 22605 | 10 | 0.83(0.24-2.88) | 0.778 | 0.82(0.24-2.88) | 0.761 | 0.77(0.23-2.70) | 0.685 |
|  | P for trend | 93234 | 29 |  | 0.866 |  | 0.850 |  | 0.762 |

**Table S16 The associations between CDAI and dementia outcomes in age restricted models (＜60 years) ^1^**

^1^Estimated effects were calculated using Cox regression model. Models were adjusted, and CDAI quartiles were defined, as described in Table S6.

CDAI, composite dietary antioxidant index; ACD, all-cause dementia; AD, Alzheimer’s dementia, VD, vascular dementia. HR, hazard ratio; CI, confidence interval.

**Table S17 The associations between CDAI and dementia outcomes in age restricted models (≥60 years) ^1^**

|  | **CDAI** | **No. of participants** | **No. dementia cases** | **Model 1** | | **Model 2** | | **Model 3** | |
| --- | --- | --- | --- | --- | --- | --- | --- | --- | --- |
|  |  |  |  | **HR (95%CI)** | ***P*** | **HR (95%CI)** | ***P*** | **HR (95%CI)** | ***P*** |
|  | Per 1-unit increase | 64508 | 1572 | 0.98(0.97-1.01) | 0.286 | 0.98(0.97-1.01) | 0.210 | 0.98(0.97-1.01) | 0.193 |
|  | Quartile 1 | 14662 | 384 | Ref. |  | Ref. |  | Ref. |  |
| ACD | Quartile 2 | 16349 | 374 | 0.84(0.73-0.98) | **0.022** | 0.85(0.74-0.99) | **0.037** | 0.86(0.74-0.99) | **0.045** |
|  | Quartile 3 | 16666 | 385 | 0.84(0.71-0.98) | **0.026** | 0.85(0.72-0.99) | **0.042** | 0.85(0.73-0.99) | **0.049** |
|  | Quartile 4 | 16831 | 429 | 0.87(0.73-1.05) | 0.158 | 0.87(0.72-1.04) | 0.130 | 0.87(0.73-1.04) | 0.132 |
|  | P for trend | 64508 | 1572 |  | 0.188 |  | 0.160 |  | 0.160 |
|  | Per 1-unit increase | 64508 | 703 | 1.002(0.97-1.03) | 0.850 | 0.99(0.91-1.03) | 0.996 | 0.99(0.97-1.03) | 0.914 |
|  | Quartile 1 | 14662 | 185 | Ref. |  | Ref. |  | Ref. |  |
| AD | Quartile 2 | 16349 | 164 | 0.79(0.64-0.99) | **0.038** | 0.79(0.64-0.99) | **0.039** | 0.79(0.64-0.99) | **0.040** |
|  | Quartile 3 | 16666 | 160 | 0.77(0.61-0.98) | **0.031** | 0.77(0.60-0.97) | **0.028** | 0.76(0.60-0.97) | **0.026** |
|  | Quartile 4 | 16831 | 194 | 0.92(0.70-1.20) | 0.532 | 0.90(0.68-1.18) | 0.425 | 0.89(0.678-1.17) | 0.388 |
|  | P for trend | 64508 | 703 |  | 0.501 |  | 0.404 |  | 0.364 |
|  | Per 1-unit increase | 64508 | 293 | 0.99(0.95-1.05) | 0.931 | 0.99(0.95-1.04) | 0.881 | 0.99(0.95-1.04) | 0.851 |
|  | Quartile 1 | 14662 | 64 | Ref. |  | Ref. |  | Ref. |  |
| VD | Quartile 2 | 16349 | 79 | 1.11(0.79-1.57) | 0.535 | 1.18(0.84-1.67) | 0.345 | 1.18(0.84-1.67) | 0.328 |
|  | Quartile 3 | 16666 | 67 | 0.92(0.63-1.35) | 0.690 | 0.98(0.67-1.44) | 0.930 | 0.98(0.67-1.44) | 0.938 |
|  | Quartile 4 | 16831 | 83 | 1.09(0.71-1.68) | 0.678 | 1.12(0.72-1.70) | 0.644 | 1.10(0.72-1.70) | 0.654 |
|  | P for trend | 64508 | 293 |  | 0.949 |  | 0.916 |  | 0.932 |

^1^Estimated effects were calculated using Cox regression model. Models were adjusted, and CDAI quartiles were defined, as described in Table S6.

CDAI, composite dietary antioxidant index; ACD, all-cause dementia; AD, Alzheimer’s dementia, VD, vascular dementia. HR, hazard ratio; CI, confidence interval.

|  | **CDAI** | **No. of participants** | **No. dementia cases** | **Model 1** | | **Model 2** | | **Model 3** | |
| --- | --- | --- | --- | --- | --- | --- | --- | --- | --- |
|  |  |  |  | **HR (95%CI)†** | ***P*** | **HR (95%CI)†** | ***P*** | **HR (95%CI)†** | ***P*** |
|  | Per 1-unit increase | 44422 | 989 | 0.97(0.95-0.99) | **0.019** | 0.97(0.94-0.99) | **0.015** | 0.98(0.94-0.99) | **0.014** |
|  | Quartile 1 | 10990 | 239 | Ref. |  | Ref. |  | Ref. |  |
| ACD | Quartile 2 | 11049 | 255 | 0.90(0.75-1.08) | 0.257 | 0.91(0.76-1.10) | 0.334 | 0.91(0.76-1.10) | 0.342 |
|  | Quartile 3 | 11030 | 235 | 0.80(0.66-0.98) | **0.034** | 0.81(0.66-0.99) | **0.042** | 0.81(0.66-0.99) | **0.044** |
|  | Quartile 4 | 11353 | 260 | 0.79(0.63-1.00) | 0.054 | 0.79(0.62-1.00) | **0.050** | 0.79(0.62-0.99) | **0.048** |
|  | P for trend | 44422 | 989 |  | 0.035 |  | **0.030** |  | **0.029** |
|  | Per 1-unit increase | 44422 | 517 | 0.97(0.93-1.00) | 0.086 | 0.97(0.93-1.00) | 0.060 | 0.97(0.93-1.00) | 0.056 |
|  | Quartile 1 | 10990 | 136 | Ref. |  | Ref. |  | Ref. |  |
| AD | Quartile 2 | 11049 | 132 | 0.84(0.65-1.07) | 0.162 | 0.84(0.65-1.08) | 0.179 | 0.84(0.65-1.08) | 0.178 |
|  | Quartile 3 | 11030 | 119 | 0.74(0.56-0.98) | **0.036** | 0.74(0.56-0.97) | **0.030** | 0.73(0.56-0.97) | **0.029** |
|  | Quartile 4 | 11353 | 130 | 0.74(0.54-1.03) | 0.073 | 0.73(0.52-1.00) | **0.052** | 0.72(0.52-0.99) | **0.049** |
|  | P for trend | 44422 | 517 |  | 0.054 |  | 0.036 |  | 0.034 |
|  | Per 1-unit increase | 44422 | 157 | 0.97(0.91-1.03) | 0.311 | 0.97(0.91-1.03) | 0.310 | 0.97(0.91-1.03) | 0.302 |
|  | Quartile 1 | 10990 | 34 | Ref. |  | Ref. |  | Ref. |  |
| VD | Quartile 2 | 11049 | 45 | 1.22(0.77-1.95) | 0.402 | 1.33(0.84-2.14) | 0.224 | 1.34(0.84-2.15) | 0.218 |
|  | Quartile 3 | 11030 | 40 | 1.09(0.65-1.81) | 0.750 | 1.18(0.70-1.98) | 0.529 | 1.18(0.70-1.97) | 0.532 |
|  | Quartile 4 | 11353 | 38 | 0.96(0.52-1.76) | 0.900 | 1.00(0.55-1.86) | 0.978 | 1.00(0.54-1.86) | 0.980 |
|  | P for trend | 44422 | 157 |  | 0.778 |  | 0.871 |  | 0.865 |

**Table S18 The associations between CDAI and dementia outcomes in APOEε4 restricted models (APOEε4 carrier) ^1^**

^1^Estimated effects were calculated using Cox regression model. Models were adjusted, and CDAI quartiles were defined, as described in Table S6.

CDAI, composite dietary antioxidant index; ACD, all-cause dementia; AD, Alzheimer’s dementia, VD, vascular dementia. HR, hazard ratio; CI, confidence interval.

**Table S19 The associations between CDAI and dementia outcomes in APOEε4 restricted models (APOEε4 non-carrier) ^1^**

|  | **CDAI** | **No. of participants** | **No. dementia cases** | **Model 1** | | **Model 2** | | **Model 3** | |
| --- | --- | --- | --- | --- | --- | --- | --- | --- | --- |
|  |  |  |  | **HR (95%CI)†** | ***P*** | **HR (95%CI)†** | ***P*** | **HR (95%CI)†** | ***P*** |
|  | Per 1-unit increase | 113320 | 833 | 0.98(0.96-1.01) | 0.345 | 0.98(0.96-1.01) | 0.332 | 0.98(0.96-1.01) | 0.370 |
|  | Quartile 1 | 28446 | 219 | Ref. |  | Ref. |  | Ref. |  |
| ACD | Quartile 2 | 28386 | 179 | 0.70(0.57-0.87) | **<0.001** | 0.72(0.58-0.88) | **0.001** | 0.72(0.59-0.89) | **0.002** |
|  | Quartile 3 | 28405 | 192 | 0.70(0.56-0.87) | **0.001** | 0.71(0.58-0.89) | **0.002** | 0.73(0.58-0.90) | **0.004** |
|  | Quartile 4 | 28083 | 243 | 0.81(0.63-1.04) | 0.092 | 0.81(0.63-1.04) | 0.092 | 0.82(0.64-1.05) | 0.110 |
|  | P for trend | 113320 | 833 |  | 0.112 |  | 0.116 |  | 0.142 |
|  | Per 1-unit increase | 113320 | 274 | 1.02(0.97-1.07) | 0.457 | 1.02(0.97-1.07) | 0.476 | 1.02(0.97-1.07) | 0.499 |
|  | Quartile 1 | 28446 | 74 | Ref. |  | Ref. |  | Ref. |  |
| AD | Quartile 2 | 28386 | 57 | 0.70(0.49-0.99) | **0.049** | 0.70(0.49-0.99) | **0.050** | 0.70(0.49-0.99) | **0.049** |
|  | Quartile 3 | 28405 | 54 | 0.63(0.43-0.93) | **0.021** | 0.63(0.43-0.93) | **0.021** | 0.63(0.43-0.93) | **0.021** |
|  | Quartile 4 | 28083 | 89 | 1.01(0.66-1.54) | 0.966 | 1.00(0.66-1.53) | 0.994 | 0.99(0.65-1.52) | 0.985 |
|  | P for trend | 113320 | 274 |  | 0.945 |  | 0.926 |  | 0.913 |
|  | Per 1-unit increase | 113320 | 165 | 1.01(0.95-1.08) | 0.716 | 1.01(0.95-1.07) | 0.754 | 1.01(0.95-1.07) | 0.773 |
|  | Quartile 1 | 28446 | 39 | Ref. |  | Ref. |  | Ref. |  |
| VD | Quartile 2 | 28386 | 38 | 0.83(0.52-1.32) | 0.432 | 0.86(0.54-1.37) | 0.526 | 0.86(0.54-1.36) | 0.517 |
|  | Quartile 3 | 28405 | 33 | 0.66(0.40-1.09) | 0.107 | 0.69(0.41-1.14) | 0.146 | 0.69(0.41-1.14) | 0.147 |
|  | Quartile 4 | 28083 | 55 | 0.98(0.57-1.72) | 0.970 | 0.99(0.57-1.73) | 0.978 | 0.98(0.57-1.71) | 0.956 |
|  | P for trend | 113320 | 165 |  | 0.817 |  | 0.821 |  | 0.807 |

^1^Estimated effects were calculated using Cox regression model. Models were adjusted, and CDAI quartiles were defined, as described in Table S6.

CDAI, composite dietary antioxidant index; ACD, all-cause dementia; AD, Alzheimer’s dementia, VD, vascular dementia. HR, hazard ratio; CI, confidence interval.

|  | **CDAI** | **No. of participants** | **No. dementia cases** | **Model 1** | | **Model 2** | | **Model 3** | |
| --- | --- | --- | --- | --- | --- | --- | --- | --- | --- |
|  |  |  |  | **HR (95%CI)†** | ***P*** | **HR (95%CI)†** | ***P*** | **HR (95%CI)†** | ***P*** |
|  | Per 1-unit increase | 60398 | 647 | 0.96(0.93-0.99) | **0.004** | 0.96(0.93-0.99) | **0.008** | 0.96(0.93-0.99) | **0.010** |
|  | Quartile 1 | 13735 | 138 | Ref. |  | Ref. |  | Ref. |  |
| ACD | Quartile 2 | 15209 | 153 | 0.78(0.62-0.99) | **0.045** | 0.80(0.63-1.01) | 0.062 | 0.80(0.63-1.02) | 0.075 |
|  | Quartile 3 | 15888 | 169 | 0.76(0.59-0.97) | **0.028** | 0.78(0.60-0.99) | **0.047** | 0.79(0.61-1.01) | 0.064 |
|  | Quartile 4 | 15566 | 187 | 0.72(0.54-0.95) | **0.023** | 0.73(0.54-0.97) | **0.031** | 0.74(0.55-0.98) | **0.038** |
|  | P for trend | 60398 | 647 |  | 0.035 |  | 0.048 |  | 0.058 |
|  | Per 1-unit increase | 60398 | 303 | 0.98(0.93-1.02) | 0.293 | 0.98(0.94-1.02) | 0.334 | 0.98(0.94-1.03) | 0.381 |
|  | Quartile 1 | 13735 | 67 | Ref. |  | Ref. |  | Ref. |  |
| AD | Quartile 2 | 15209 | 65 | 0.70(0.49-0.99) | 0.047 | 0.71(0.50-1.01) | 0.055 | 0.71(0.50-1.01) | 0.058 |
|  | Quartile 3 | 15888 | 76 | 0.73(0.51-1.05) | 0.088 | 0.74(0.52-1.07) | 0.111 | 0.75(0.52-1.09) | 0.131 |
|  | Quartile 4 | 15566 | 95 | 0.80(0.53-1.21) | 0.294 | 0.82(0.54-1.23) | 0.333 | 0.82(0.54-1.25) | 0.362 |
|  | P for trend | 60398 | 303 |  | 0.443 |  | 0.496 |  | 0.541 |
|  | Per 1-unit increase | 60398 | 88 | 0.94(0.87-1.02) | 0.159 | 0.95(0.87-1.03) | 0.203 | 0.94(0.87-1.02) | 0.151 |
|  | Quartile 1 | 13735 | 19 | Ref. |  | Ref. |  | Ref. |  |
| VD | Quartile 2 | 15209 | 20 | 0.71(0.37-1.36) | 0.308 | 0.72(0.38-1.39) | 0.332 | 0.70(0.37-1.35) | 0.290 |
|  | Quartile 3 | 15888 | 21 | 0.63(0.32-1.25) | 0.184 | 0.66(0.33-1.31) | 0.231 | 0.63(0.32-1.26) | 0.194 |
|  | Quartile 4 | 15566 | 28 | 0.68(0.32-1.48) | 0.334 | 0.68(0.32-1.48) | 0.334 | 0.64(0.29-1.40) | 0.264 |
|  | P for trend | 60398 | 88 |  | 0.352 |  | 0.365 |  | 0.293 |

**Table S20 The associations between CDAI and dementia outcomes in BMI restricted models ((BMI＜25kg/m^2^) ^1^**

^1^Estimated effects were calculated using Cox regression model. Models were adjusted, and CDAI quartiles were defined, as described in Table S6.

CDAI, composite dietary antioxidant index; ACD, all-cause dementia; AD, Alzheimer’s dementia, VD, vascular dementia. HR, hazard ratio; CI, confidence interval.

|  | **CDAI** | **No. of participants** | **No. dementia cases** | **Model 1** | | **Model 2** | | **Model 3** | |
| --- | --- | --- | --- | --- | --- | --- | --- | --- | --- |
|  |  |  |  | **HR (95%CI)** | ***P*** | **HR (95%CI)** | ***P*** | **HR (95%CI)** | ***P*** |
|  | Per 1-unit increase | 97344 | 1175 | 0.98(0.96-1.00) | 0.070 | 0.98(0.96-1.00) | 0.098 | 0.98(0.96-1.00) | 0.102 |
|  | Quartile 1 | 25701 | 320 | Ref. |  | Ref. |  | Ref. |  |
| ACD | Quartile 2 | 24226 | 281 | 0.79(0.67-0.94) | **0.006** | 0.82(0.70-0.97) | **0.021** | 0.80(0.63-1.02) | **0.024** |
|  | Quartile 3 | 23547 | 258 | 0.71(0.59-0.85) | **<0.001** | 0.74(0.62-0.87) | **0.001** | 0.79(0.61-1.01) | **0.002** |
|  | Quartile 4 | 23870 | 316 | 0.79(0.64-0.98) | **0.029** | 0.81(0.66-1.00) | 0.052 | 0.74(0.55-0.98) | 0.058 |
|  | P for trend | 97344 | 1175 |  | **0.015** |  | **0.028** |  | **0.033** |
|  | Per 1-unit increase | 97344 | 488 | 0.98(0.95-1.02) | 0.357 | 0.99(0.95-1.02) | 0.399 | 0.98(0.95-1.02) | 0.387 |
|  | Quartile 1 | 25701 | 143 | Ref. |  | Ref. |  | Ref. |  |
| AD | Quartile 2 | 24226 | 124 | 0.82(0.64-1.06) | 0.128 | 0.84(0.65-1.08) | 0.183 | 0.84(0.65-1.08) | 0.183 |
|  | Quartile 3 | 23547 | 97 | 0.65(0.49-0.86) | **0.003** | 0.67(0.50-0.89) | **0.006** | 0.67(0.50-0.89) | **0.006** |
|  | Quartile 4 | 23870 | 124 | 0.80(0.58-1.11) | 0.180 | 0.81(0.59-1.13) | 0.214 | 0.81(0.59-1.13) | 0.214 |
|  | P for trend | 97344 | 488 |  | 0.068 |  | 0.088 |  | 0.089 |
|  | Per 1-unit increase | 97344 | 234 | 0.99(0.94-1.04) | 0.618 | 0.99(0.94-1.04) | 0.703 | 0.99(0.95-1.04) | 0.771 |
|  | Quartile 1 | 25701 | 54 | Ref. |  | Ref. |  | Ref. |  |
| VD | Quartile 2 | 24226 | 63 | 1.06(0.73-1.54) | 0.77 | 1.14(0.78-1.67) | 0.494 | 1.15(0.79-1.69) | 0.453 |
|  | Quartile 3 | 23547 | 52 | 0.85(0.56-1.29) | 0.45 | 0.92(0.60-1.40) | 0.691 | 0.94(0.62-1.43) | 0.768 |
|  | Quartile 4 | 23870 | 65 | 0.96(0.60-1.54) | 0.86 | 1.01(0.63-1.63) | 0.972 | 1.03(0.64-1.67) | 0.896 |
|  | P for trend | 97344 | 234 |  | 0.621 |  | 0.764 |  | 0.842 |

**Table S21The associations between CDAI and dementia outcomes in BMI restricted models ((BMI≥25kg/m^2^) ^1^**

^1^Estimated effects were calculated using Cox regression model. Models were adjusted, and CDAI quartiles were defined, as described in Table S6.

CDAI, composite dietary antioxidant index; ACD, all-cause dementia; AD, Alzheimer’s dementia, VD, vascular dementia. HR, hazard ratio; CI, confidence interval.

| **Interaction terms** |  | All-cause dementia | Alzheimer’s dementia | vascular dementia |
| --- | --- | --- | --- | --- |
| **Variables × CDAI** |  | **P for Interaction** | **P for Interaction** | **P for Interaction** |
| **Sex (female vs. male)** | Continuous CDAI | **0.017** | **0.011** | 0.889 |
|  | Quartiled CDAI | 0.062 | 0.060 | 0.662 |
| **Age stage (≥60 years vs. ＜60 years)** | Continuous CDAI | 0.508 | 0.267 | 0.943 |
|  | Quartiled CDAI | 0.141 | 0.335 | 0.463 |
| **APOE-ε4 carriers vs. non-carriers** | Continuous CDAI | 0.391 | 0.089 | 0.425 |
|  | Quartiled CDAI | 0.162 | 0.126 | 0.213 |
| **BMI (＜25kg/m^2^ vs. ≥25kg/m^2^)** | Continuous CDAI | 0.366 | 0.833 | 0.322 |
|  | Quartiled CDAI | 0.722 | 0.602 | 0.600 |

**Table S22 Modification for the association between CDAI and risk of incident dementia.**

Model was adjusted for the variables in Model 3, as described in Table S6. CDAI quartiles were defined as described in Table S6.

The P value was not adjusted for multiple comparison correction.

CDAI, composite dietary antioxidant index.

**Table S23 Comparison of blood inflammatory profiles between dementia cases and non-dementia cases.**

| inflammatory markers | Overall | Non-dementia | Dementia | p value |
| --- | --- | --- | --- | --- |
| N | 152700 | 150927 | 1773 |  |
| Leukocyte count, 10^9 cells/L | 6.72±1.95 | 6.72±1.95 | 6.88±1.74 | **<0.001** |
| Neutrophil count，10^9 cells/L | 4.11±1.35 | 4.11±1.35 | 4.29±1.32 | **<0.001** |
| Neutrophil percentage | 60.70±8.45 | 60.68±8.45 | 61.93±8.50 | **<0.001** |
| Monocyte count，10^9 cells/L | 0.47±0.21 | 0.47±0.21 | 0.49±0.18 | **<0.001** |
| Monocyte percentage | 7.11±2.75 | 7.11±2.76 | 7.17±2.20 | 0.425 |
| Lymphocyte count，10^9 cells/L | 1.93±1.10 | 1.93±1.10 | 1.90±0.75 | 0.224 |
| Lymphocyte percentage | 29.06±7.40 | 29.07±7.39 | 27.78±7.68 | **<0.001** |
| Platelet count，10^9 cells/L | 249.02±58.07 | 249.09±58.04 | 242.83±60.17 | **<0.001** |
| C reactive protein, mg/L | 2.27±3.87 | 2.27±3.86 | 2.39±4.37 | 0.194 |
| SII，10^9 cells/L | 582.72±349.65 | 582.35±349.20 | 614.37±385.16 | **<0.001** |
| NLR | 2.33±1.20 | 2.33±1.20 | 2.53±1.33 | **<0.001** |
| LMR | 4.58±3.74 | 4.59±3.75 | 4.31±2.24 | **0.002** |
| PLR | 141.76±61.57 | 141.74±61.54 | 143.20±63.91 | 0.322 |

Abbreviations: SII, systemic immune inflammation index, NLR, neutrophil-to-lymphocyte ratio; LMR, lymphocyte-to-monocyte ratio; PLR, platelet-to-lymphocyte ratio.

**Table S24 Baseline** **demographic and clinical characteristics of 152700 participants in the inflammatory profiles analyses**

| **Characteristic** | **Overall** | **lower group**  **(CDAI≤1.579)** | **higher group**  **(CDAI＞1.579)** | **p value** |
| --- | --- | --- | --- | --- |
| **N** | 152700 | 107460 | 45240 |  |
| **Age, years, M(IQR)** | 57.00 (50.00-63.00) | 57.00 (50.00-62.00) | 58.00 (50.00-63.00) | <0.001 |
| **Sex, n (%)** |  |  |  | <0.001 |
| Female | 84377 (55.26%) | 62089 (57.78%) | 22288 (49.27%) |  |
| Male | 68323 (44.74%) | 45371 (42.22%) | 22952 (50.73%) |  |
| **Race, n(%)** |  |  |  | <0.001 |
| White | 139129 (91.11%) | 98138 (91.33%) | 40991 (90.61%) |  |
| Mixed | 4820 (3.16%) | 3393 (3.16%) | 1427 (3.15%) |  |
| Asian | 6366 (4.17%) | 4273 (3.98%) | 2093 (4.63%) |  |
| Other | 2385 (1.56%) | 1656 (1.54%) | 729 (1.61%) |  |
| **Education, M(IQR)** | 15.00 (10.00-20.00) | 15.00 (10.00-20.00) | 19.00 (10.00-20.00) | <0.001 |
| **TDI, M(IQR)** | -2.36 (-3.75--0.03) | -2.36 (-3.75--0.02) | -2.37 (-3.76--0.04) | 0.271 |
| **BMI, kgm^−2^,** **M(IQR)** | 26.14 (23.67-29.18) | 26.19 (23.72-29.24) | 26.02 (23.55-29.04) | <0.001 |
| **APOE-ε4 carrier, n (%)** |  |  |  | <0.001 |
| Non-carrier | 109692 (71.83%) | 77521 (72.14%) | 32171 (71.11%) |  |
| Carrier | 43008(28.16%) | 29939(27.86%) | 13069 (28.89%) |  |
| **Smoking, n(%)** |  |  |  | <0.001 |
| Never | 87033 (57.00%) | 61086 (56.85%) | 25947 (57.35%) |  |
| Previous | 54843 (35.92%) | 38432 (35.76%) | 16411 (36.28%) |  |
| Current | 10824 (7.09%) | 7942 (7.39%) | 2882 (6.37%) |  |
| **Alcohol, n(%)** |  |  |  | 0.016 |
| Never | 4909 (3.21%) | 3503 (3.26%) | 1406 (3.11%) |  |
| Previous | 4557 (2.98%) | 3132 (2.91%) | 1425 (3.15%) |  |
| Current | 143234 (93.80%) | 100825 (93.83%) | 42409 (93.74%) |  |
| **Diabetes, n(%)** |  |  |  | 0.143 |
| No | 149379 (97.83%) | 105161 (97.86%) | 44218 (97.74%) |  |
| Yes | 3321 (2.17%) | 2299 (2.14%) | 1022 (2.26%) |  |
| **Hypertension, n(%)** |  |  |  | 0.177 |
| No | 125559 (82.23%) | 88452 (82.31%) | 37107 (82.02%) |  |
| Yes | 27141 (17.77%) | 19008 (17.69%) | 8133 (17.98%) |  |
| **Hyperlipemia, n(%)** |  |  |  | 0.839 |
| No | 148612 (97.32%) | 104589 (97.33%) | 44023 (97.31%) |  |
| Yes | 4088 (2.68%) | 2871 (2.67%) | 1217 (2.69%) |  |
| **CVD, n(%)** |  |  |  | 0.244 |
| No | 147681 (96.71%) | 103965 (96.75%) | 43716 (96.63%) |  |
| Yes | 5019 (3.29%) | 3495 (3.25%) | 1524 (3.37%) |  |
| **Stroke, n(%)** |  |  |  | 0.756 |
| No | 152191 (99.67%) | 107105 (99.67%) | 45086 (99.66%) |  |
| Yes | 509 (0.33%) | 355 (0.33%) | 154 (0.34%) |  |
| **Cancer, n(%)** |  |  |  | 0.015 |
| No | 141304 (92.54%) | 99554 (92.64%) | 41750 (92.29%) |  |
| Yes | 11396 (7.46%) | 7906 (7.36%) | 3490 (7.71%) |  |
| **supplements, n(%)** |  |  |  | <0.001 |
| No | 102718 (67.27%) | 73336 (68.24%) | 29382 (64.95%) |  |
| Yes | 49982 (32.73%) | 34124 (31.76%) | 15858 (35.05%) |  |
| **Energy(kcal**), **M(IQR)** | 1981.82 (1652.49-2359.50) | 1821.34 (1541.93-2109.69) | 2455.07 (2140.55-2836.77) | <0.001 |

Abbreviations: CDAI, composite dietary antioxidant index; M(IQR), median (interquartile range); CVD, cardiovascular disease; TDI, Townsend Deprivation Index; BMI, Body mass index.

**Table S25 Multivariate linear regression of CDAI and blood inflammatory profiles in the overall participants (n=152700)**

|  | Model1 | | | | Model2 | | | |
| --- | --- | --- | --- | --- | --- | --- | --- | --- |
|  | β | 95%CI | P-value | **P value FDR** | β | 95%CI | P-value | **P value FDR** |
| Leukocyte count, 10^9 cells/L | -0.015 | -0.017, -0.012 | <2E-16 | <2.6E-15 | -2.39E-2 | -2.78E-2, -2.00E-2 | <2E-16 | **<2.6E-15** |
| Neutrophil count, 10^9 cells/L | -0.010 | -0.012, -0.008 | <2E-16 | <2.6E-15 | -1.68E-2 | -1.95E-2, -1.41E-2 | < 2E-16 | **<2.6E-15** |
| Neutrophil percentage, % | -0.009 | -0.020, 0.003 | 0.149 | 0.161 | -3.79E-2 | -5.79E-2, -0.023 | 4.05E-6 | **5.27E-6** |
| Monocyte count, 10^9 cells/L | 0.0004 | 0.0001, 0.0007 | 0.003 | 0.0039 | -1.58E-3 | -1.99E-3, -1.17E-3 | 3.99E-14 | **8.65E-14** |
| Monocyte percentage, % | 0.022 | 0.018, 0.025 | <2E-16 | <2.6E-15 | -6.45E-4 | -6.16E-3, 4.87E-3 | 0.818 | 0.818 |
| Lymphocyte count, 10^9 cells/L | -0.005 | -0.007, -0.004 | 6.31E-12 | 1.17E-11 | -4.17E-3 | -6.40E-3, -1.93E-3 | 2.58E-4 | **3.04 E-4** |
| Lymphocyte percentage, % | -0.018 | -0.028, -0.008 | 6.13E-4 | 8.85E-4 | 4.81E-2 | 0.036,0.064 | 1.85E-11 | **3.44E-11** |
| C reactive protein, mg/L | -0.034 | -0.040, -0.029 | <2E-16 | <2.6E-15 | -3.28E-2 | -4.07E-2, -2.48E-2 | 8.42E-16 | **2.19E-15** |
| Platelet count, 10^9 cells/L | -0.815 | -0.895, -0.735 | <2E-16 | <2.6E-15 | -0.932 | -1.047, -0.817 | < 2E-16 | **<2.6E-15** |
| SII, 10^9 cells/L | -1.574 | -2.057, -1.091 | 1.65E-10 | 2.68E-10 | -3.61 | -4.56, -3.13 | < 2E-16 | **<2.6E-15** |
| NLR | 0.0015 | -0.0002,0.003 | 0.085 | 0.100 | -8.23E-3 | -8.76E-3, -3.88E-3 | 3.65E-07 | **5.27E-7** |
| PLR | -0.050 | -0.129,0.037 | 0.25 | 0.250 | -0.360 | -0.510, -0.260 | 1.59E-09 | **2.58E-9** |
| LMR | -0.024 | -0.029, -0.019 | <2E-16 | <2.6E-15 | -1.18E-3 | -8.76E-3,6.41E-3 | 0.761 | 0.824 |

Model 1: unadjusted

Molde 2 was adjusted for the variables in Model 3, as described in Table S6.

CDAI, composite dietary antioxidant index; SII, systemic immune inflammation index, NLR, neutrophil-to-lymphocyte ratio; LMR, lymphocyte-to-monocyte ratio; PLR, platelet-to-lymphocyte ratio.

**Table S26 Longitudinal associations between the risk of incident dementia with blood inflammatory profiles in the overall participants (n=152700)**

|  | All cause dementia | |  | Alzheimer’s dementia | |  | Vascular dementia | |
| --- | --- | --- | --- | --- | --- | --- | --- | --- |
|  | HR (95%CI) | P-value |  | HR (95%CI) | P-value |  | HR (95%CI) | P-value |
| Leukocyte count, 10^9 cells/L | 1.010(0.988 - 1.034) | 0.367 |  | 1.021(0.993-1.052) | 0.151 |  | 0.982(0.921-1.047 | 0.580 |
| Neutrophil count, 10^9 cells/L | 1.043(1.007-1.079) | **1.71E-02** |  | 1.062(1.008-1.118) | **2.35E-02** |  | 0.979(0.899-1.066) | 0.627 |
| Neutrophil percentage, % | 1.012(1.006-1.018) | **3.99E-05** |  | 1.012(1.004-1.021) | **5.21E-03** |  | 1.002(0.988-1.015) | 0.820 |
| Monocyte count, 10^9 cells/L | 0.864(0.677-1.10) | 0.241 |  | 0.978(0.715-1.337) | 0.484 |  | 0.942(0.579-1.532) | 0.810 |
| Lymphocyte count, 10^9 cells/L | 0.956(0.894-1.024) | 0.202 |  | 0.974(0.882-1.076) | 0.591 |  | 0.972(0.839-1.125) | 0.705 |
| Lymphocyte percentage, % | 0.987(0.981-0.994) | **1.96E-04** |  | 0.987(0.977-0.997) | **9.21E-03** |  | 0.997(0.982-1.013) | 0.740 |
| C reactive protein, mg/L | 1.005(0.996-1.018) | 0.338 |  | 1.006(0.989-1.023) | 0.641 |  | 1.001(0.974-1.029) | 0.948 |
| Platelet count, 10^9 cells/L | 0.999(0.999-1.001) | 0.829 |  | 0.999(0.998-1.001) | 0.644 |  | 1.001(0.999-1.003) | 0.261 |
| SII, 10^9 cells/L | 1.0001(1.0001-1.0002) | **1.11E-03** |  | 1.001(0.999-1.0003) | 0.327 |  | 1.0001(0.999-1.0003) | 0.303 |
| NLR | 1.039(1.019-1.058) | **3.96E-05** |  | 1.028(0.989-1.069) | 0.157 |  | 1.029(0.969-1.093) | 0.351 |
| PLR | 1.001(1.0001-1.001) | **7.73E-03** |  | 1.001(0.999-1.001) | 0.930 |  | 1.0007(0.999-1.002) | 0.119 |

Model was adjusted for the variables in Model 3, as described in Table S6.

SII, systemic immune inflammation index, NLR, neutrophil-to-lymphocyte ratio; PLR, platelet-to-lymphocyte ratio.

**Table S27 The mediation effects of blood inflammatory profiles in the association between CDAI and incident dementia risk in the overall participants (n=152700)**

|  | **All cause dementia** | | | | | | | |
| --- | --- | --- | --- | --- | --- | --- | --- | --- |
|  | **Direct effect** | | **Indirect effect** | | **Total effect** | | **Proportion of mediator** | ***P* value** |
|  | **Beta(95%CI)** | ***P* value** | **Beta(95%CI)** | ***P* value** | **Beta(95%CI)** | ***P* value** |  |  |
| Neutrophil% | -2.42E-4(-4.56E-4, -3.56E-5) | 0.016 | -4.51E-6(-7.77E-6, -1.92E-6) | <2E-16 | -2.46E-4(-4.60E-4, -4.00E-5) | 0.014 | 1.84% | **0.014** |
| Neutrophil count | -2.51E-4(-4.54E-4, -4.28E-5) | 0.024 | -4.14E-6(-9.65E-6,1.64E-6) | 0.152 | -2.64E-4(-4.60E-4, -4.52E-5) | 0.02 | 1.61% | 0.172 |
| Lymphocyte% | -2.40E-4(-4.51E-4, -3.59E-5) | 0.022 | -6.01E-6(-1.01E-6, -2.67E-6) | 0.002 | -2.46E-4(-4.60E-4, -4.19E-5) | 0.018 | 2.41% | **0.020** |
| SII | -2.50E-4(-4.50E-4, -3.98E-5) | 0.022 | -4.56E-6(-8.46E-6, -8.81E-7) | 0.008 | -2.55E-4(-4.55E-4, -4.52E-5) | 0.02 | 1.69% | **0.028** |
| NLR | -2.37E-4(-4.40E-4, -3.12E-5) | 0.030 | -2.09E-6(-3.86E-6, -5.77E-7) | 0.006 | -2.39E-4(-4.42E-4, -3.23E-5) | 0.028 | 0.83% | **0.034** |
| PLR | -2.51E-4(-4.50E-4, -3.22E-5) | 0.026 | -2.86E-6(-5.38E-6, -7.54E-7) | 0.004 | -2.54E-4(-4.54E-4, -3.48E-5) | 0.022 | 1.10% | **0.026** |
|  | **Alzheimer’s dementia** | | | | | | | |
|  | **Direct effect** | | **Indirect effect** | | **Total effect** | | **Proportion of mediator** | ***P* value** |
|  | **Beta(95%CI)** | ***P* value** | **Beta(95%CI)** | ***P* value** | **Beta(95%CI)** | ***P* value** |  |  |
| Neutrophil count | -6.61E-5(-2.07E-4,7.42E-5) | 0.344 | -3.15E-6(-7.00E-6,4.29E-7) | 0.084 | -6.92E-5(-2.1E-4,7.02E-5) | 0.332 | 2.87% | 0.376 |
| Neutrophil% | -6.46E-5(-2.12E-4,6.66E-5) | 0.366 | -2.03E-6(-4.05E-6, -4.17E-7) | 0.052 | -6.66E-5(-2.14E-4,6.39E-5) | 0.348 | 1.94% | 0.356 |
| Lymphocyte% | -6.34E-5(-1.92E-4,6.82E-5) | 0.356 | -2.78E-6(-5.45E-6, -4.93E-7) | 0.054 | -6.62E-5(-1.94E-4,6.65E-5) | 0.332 | 2.72% | 0.342 |

Model was adjusted for the variables in Model 3, as described in Table S6.

CDAI, composite dietary antioxidant index; SII, systemic immune inflammation index, NLR, neutrophil-to-lymphocyte ratio; PLR, platelet-to-lymphocyte ratio.

**Table S28 Multivariate linear regression of CDAI and blood inflammatory profiles in the lower CDAI participants (CDAI<=1.579, n=107460)**

|  | Model 1 | | | | Model 2 | | | |
| --- | --- | --- | --- | --- | --- | --- | --- | --- |
|  | β | 95%CI | P-value | P value FDR | β | 95%CI |  | P value FDR |
| Leukocyte count, 10^9 cells/L | -0.032 | -0.037, -0.027 | <2E-16 | 2.6E-15 | -0.029 | -0.035, -0.023 | <2E-16 | **2.6E-15** |
| Neutrophil count, 10^9 cells/L | -0.020 | -0.023, -0.016 | <2E-16 | 2.6E-15 | -0.021 | -0.026, -0.017 | <2E-16 | **2.6E-15** |
| Neutrophil percentage, % | -0.013 | -0.035,0.009 | 0.247 | 0.3211 | -0.062 | -0.091, -0.033 | 2.95E-5 | **4.26E-05** |
| Monocyte count, 10^9 cells/L | -6.46E-5 | -0.0006, 0.0005 | 0.816 | 0.884 | -1.41E-3 | -2.10E-3, -7.24E-3 | 5.84E-5 | **7.59E-5** |
| Monocyte percentage, % | 0.028 | 0.021, 0.035 | 5.17E-14 | 1.12E-13 | 5.87E-3 | -6.16E-3, 4.87E-3 | 0.213 | 0.213 |
| Lymphocyte count, 10^9 cells/L | -0.011 | -0.013, -0.008 | 1.46E-15 | 3.80E-15 | -4.79E-3 | -8.20E-3, -1.37E-3 | 0.006 | **0.007** |
| Lymphocyte percentage, % | -0.016 | -0.036, -0.003 | 0.108 | 0.156 | 0.069 | 0.044,0.094 | 8.17E-8 | **1.52E-07** |
| C reactive protein, mg/L | -0.068 | -0.078, -0.057 | <2E-16 | 2.6E-15 | -0.050 | -0.624, -0.037 | 5.08E-14 | **1.32E-13** |
| Platelet count, 10^9 cells/L | -0.938 | -1.092, -0.784 | <2E-16 | 2.6E-15 | -1.287 | -1.481, -1.093 | <2E-16 | **2.6E-15** |
| SII, 10^9 cells/L | -2.12 | -3.052, -1.196 | 7.26E-06 | 1.18E-05 | -5.18 | -6.38, -3.97 | <2E-16 | **2.6E-15** |
| NLR | -9.72E-5 | -0.003,0.003 | 0.954 | 0.954 | -9.25E-3 | -0.013, -0.005 | 6.73E-6 | **1.09E-05** |
| PLR | 0.078 | -0.082,0.238 | 0.338 | 0.399 | -0.619 | -0.826, -0.413 | 3.95E-9 | **8.56E-09** |
| LMR | -0.039 | -0.049, -0.028 | 9.61E-14 | 1.78E-13 | -9.44E-3 | -0.023, -0.004 | 0.158 | 0.171 |

Model 1: unadjusted

Model 2 was adjusted for the variables in Model 3, as described in Table S6.

CDAI, composite dietary antioxidant index; SII, systemic immune inflammation index; NLR, neutrophil-to-lymphocyte ratio; LMR, lymphocyte-to-monocyte ratio; PLR, platelet-to-lymphocyte ratio.

**Table S29 Associations between dementia with blood inflammatory profiles in the lower CDAI participants (CDAI<=1.579, n=107460)**

|  | All cause dementia | |  | Alzheimer’s dementia | |  | Vascular dementia | |
| --- | --- | --- | --- | --- | --- | --- | --- | --- |
|  | HR (95%CI) | P-value |  | HR (95%CI) | P-value |  | HR (95%CI) | P-value |
| Leukocyte count, 10^9 cells/L | 1.013(0.985-1.041) | 0.371 |  | 1.029(0.998-1.061) | 0.069 |  | 0.935(0.859-1.018) | 0.123 |
| Neutrophil count, 10^9 cells/L | 1.046(1.003-1.091) | **3.53E-02** |  | 1.08(1.015-1.149) | **1.46E-02** |  | 0.925(0.830-1.03) | 0.155 |
| Neutrophil percentage, % | 1.012(1.005-1.019) | **4.77E-04** |  | 1.015(1.004-1.026) | **5.76E-03** |  | 0.998(0.982-1.014) | 0.813 |
| Monocyte count, 10^9 cells/L | 0.892(0.671-1.186) | 0.433 |  | 0.969(0.663-1.415) | 0.868 |  | 0.745(0.348-1.596) | 0.449 |
| Lymphocyte count, 10^9 cells/L | 0.958(0.884-1.039) | 0.306 |  | 0.975(0.870-1.093) | 0.667 |  | 0.944(0.771-1.544) | 0.573 |
| Lymphocyte percentage, % | 0.987(0.979-0.995) | **9.64E-04** |  | 0.983(0.971-0.995) | **5.59E-03** |  | 1.004(0.986-1.023) | 0.670 |
| C reactive protein, mg/L | 1.008(0.995-1.020) | 0.246 |  | 1.006(0.986-1.026) | 0.558 |  | 0.994(0.958-1.031) | 0.736 |
| Platelet count, 10^9 cells/L | 0.999(0.998-1.00) | 0.805 |  | 0.999(0.998-1.001) | 0.807 |  | 1.001(0.998-1.003) | 0.609 |
| SII, 10^9 cells/L | 1(1.000-1.0003) | 0.059 |  | 1(0.999-1.00) | 0.210 |  | 1.00(0.999-1.00) | 0.816 |
| NLR | 1.049(1.0141-1.086) | **5.67E-03** |  | 1.052(0.998-1.109) | 0.059 |  | 0.993(0.888-1.11) | 0.898 |
| PLR | 1.001(0.999-1.001) | 0.104 |  | 1.00(0.998-1.002) | 0.759 |  | 1.001(0.999-1.003) | 0.357 |

Model was adjusted for the variables in Model 3, as described in Table S6.

CDAI, composite dietary antioxidant index; SII, systemic immune inflammation index; NLR, neutrophil-to-lymphocyte ratio; PLR, platelet-to-lymphocyte ratio.

**Table S30 The mediation effects of blood inflammatory profiles in the association between CDAI and incident dementia risk in the lower CDAI participants (CDAI<=1.579, n=107460)**

|  | **All cause dementia** | | | | | | | |
| --- | --- | --- | --- | --- | --- | --- | --- | --- |
|  | **Direct effect** | | **Indirect effect** | | **Total effect** | | **Proportion of mediator** | ***P* value** |
|  | **Beta(95%CI)** | ***P* value** | **Beta(95%CI)** | ***P* value** | **Beta(95%CI)** | ***P* value** |  |  |
| Neutrophil% | -5.80E-4(-8.45E-4, -3.03E-4) | 2E-16 | -6.46E-6(-1.22E-5, -2.02E-6) | **0.002** | -5.87E-4(-8.52E-4, -3.10E-4) | 2E-16 | 1.05% | **0.002** |
| Neutrophil count | -5.84E-4(-8.50E-4, -3.03E-4) | 2E-16 | -4.76E-6(-1.21E-5,2.07E-6) | 0.206 | -5.89E-4(-8.52E-4, -3.09E-4) | 2E-16 | 0.76% | 0.206 |
| Lymphocyte% | -5.77E-4(-8.42E-4, -2.77E-4) | 2E-16 | -7.90E-6(-1.47E-5, -2.35E-6) | **0.004** | -5.85E-4(-8.49E-4, -2.84E-4) | 2E-16 | 1.32% | **0.004** |
| NLR | -5.91E-4(-8.51E-4, -2.94E-4) | 2E-16 | -2.62E-6(-6.25E-6,4.62E-7) | 0.094 | -5.94E-4(-8.50E-4, -2.96E-4) | 2E-16 | 0.42% | 0.094 |
|  | **Alzheimer’s dementia** | | | | | | | |
|  | **Direct effect** | | **Indirect effect** | | **Total effect** | | **Proportion of mediator** | ***P* value** |
|  | **Beta(95%CI)** | ***P* value** | **Beta(95%CI)** | ***P* value** | **Beta(95%CI)** | ***P* value** |  |  |
| Neutrophil count | -2.78E-4(-4.51E-4, -7.32E-5) | 0.012 | -4.64E-6(-9.84E-4, -1.68E-7) | 0.046 | -2.83E-4(-4.55E-4, -7.73E-5) | 0.012 | 1.56% | 0.058 |
| Neutrophil% | -2.75E-4(-4.28E-4, -7.98E-5) | 0.010 | -3.35E-6(-7.27E-6, -7.41E-7) | **0.008** | -2.78E-4(-4.41E-4, -8.74E-5) | 0.01 | 1.15% | **0.018** |
| Lymphocyte% | -2.77E-4(-4.45E-4, -6.89E-5) | 0.008 | -4.54E-4(-8.74E-6, -9.90E-7) | **0.014** | -2.81E-4(-4.47E-4, -7.45E-5) | 0.006 | 1.56% | **0.02** |

Model was adjusted for the variables in Model 3, as described in Table S6. CDAI, composite dietary antioxidant index; NLR, neutrophil-to-lymphocyte ratio; PLR.

**Table S31 Multivariate linear regression of CDAI and blood inflammatory profiles in the higher CDAI participants (CDAI>1.579, n=45240)**

|  | Model1 | | | | Model2 | | | |
| --- | --- | --- | --- | --- | --- | --- | --- | --- |
|  | β | 95%CI | P-value | P value FDR | β | 95%CI |  | P value FDR |
| Leukocyte count, 10^9 cells/L | 3.24E-3 | -0.0037, -0.0102 | 0.36 | 0.936 | -0.016 | -2.38E-2, -8.24E-3 | 5.64E-5 | 7.33E-04 |
| Neutrophil count, 10^9 cells/L | 1.59E-3 | -0.0029, 0.0061 | 0.488 | 0.906 | -9.55E-3 | -1.46E-2, -4.50E-3 | 2.12E-4 | 1.38E-03 |
| Neutrophil percentage, % | -0.015 | -0.044,0.0135 | 0.301 | 0.978 | -0.021 | -0.0538, -0.0115 | 0.204 | 2.41E-01 |
| Monocyte count, 10^9 cells/L | 6.78E-4 | -9.74E-6, 1.37E-3 | 0.053 | 0.345 | -1.43E-3 | -2.20E-3, -6.67E-4 | 2.48E-4 | 1.07E-03 |
| Monocyte percentage, % | 3.91E-3 | -0.0054,0.0132 | 0.411 | 0.891 | -9.97E-3 | -2.04E-2, 5.05E-4 | 0.062 | 1.01E-01 |
| Lymphocyte count, 10^9 cells/L | 3.84E-4 | -0.035,0.009 | 0.864 | 0.864 | -4.31E-3 | -9.31E-3,7.06E-4 | 0.092 | 1.33E-01 |
| Lymphocyte percentage, % | 4.23E-3 | -0.021, 0.029 | 0.74 | 0.875 | 0.0363 | 7.86E-3,6.47E-2 | 0.0124 | 4.03E-02 |
| C reactive protein, mg/L | 1.53E-3 | -0.011, 0.014 | 0.816 | 0.884 | -0.018 | -3.22E-2, -3.86E-3 | 0.013 | 2.82E-02 |
| Platelet count, 10^9 cells/L | -0.263 | -0.457, -0.0694 | 0.0076 | 0.099 | -0.273 | -0.489, --0.058 | 0.0128 | 3.33E-02 |
| SII, 10^9 cells/L | -0.254 | -1.419, 0.910 | 0.669 | 1.087 | -1.475 | -2.808, -0.142 | 0.030 | 5.57E-02 |
| NLR | 7.37E-4 | -0.0034,0.0049 | 0.73 | 1.054 | -3.35E-3 | -8.12E-3,1.42E-3 | 0.169 | 2.20E-01 |
| PLR | -0.1665 | -0.382,0.049 | 0.13 | 0.563 | -0.085 | -0.330,0.161 | 0.499 | 4.99E-01 |
| LMR | -2.06E-3 | -0.014, 0.009 | 0.731 | 0.950 | 6.52E-3 | -6.77E-3, 1.98E-2 | 0.336 | 3.64E-01 |

Model 1: unadjusted

Model 2 was adjusted for the variables in Model 3, as described in Table S6. CDAI, composite dietary antioxidant index; SII, systemic immune inflammation index; NLR, neutrophil-to-lymphocyte ratio; LMR, lymphocyte-to-monocyte ratio; PLR, platelet-to-lymphocyte ratio.

**Table S32 Associations between blood inflammatory profiles and dementia in the higher CDAI participants (CDAI>1.579, n=45240) ^1^**

|  | All cause dementia | |  | Alzheimer’s dementia | | Vascular dementia | |
| --- | --- | --- | --- | --- | --- | --- | --- |
|  | HR (95%CI) | P-value |  | HR (95%CI) | P-value | HR (95%CI) | P-value |
| Leukocyte count, 10^9 cells/L | 1.006(0.967-1.046) | 0.772 |  | 1.003(0.939-1.070) | 0.937 | 1.027(0.979-1.077) | 0.274 |
| Neutrophil count, 10^9 cells/L | 1.038(0.976-1.105) | 0.236 |  | 1.026(0.933-1.129) | 0.598 | 1.08(0.945-1.235) | 0.260 |
| Monocyte count, 10^9 cells/L | 0.814(0.513-1.294) | 0.385 |  | 0.658(0.230-1.447) | 0.298 | 1.308(0.730-2.344) | 0.367 |
| Lymphocyte percentage, % | 0.989(0.977-1.0004) | 0.059 |  | 0.998(0.977-1.00) | 0.059 | 0.974(0.958-1.011) | 0.247 |
| C reactive protein, mg/L | 0.998(0.976-1.021) | 0.865 |  | 0.997(0.963-1.0339) | 0.906 | 1.011(0.971-1.053) | 0.601 |
| Platelet count, 10^9 cells/L | 1.0001(0.997-1.002) | 0.868 |  | 0.999(0.997-1.002) | 0.581 | 1.002(0.999-1.005) | 0.187 |

^1^Model was adjusted for the variables in Model 3, as described in Table S6. CDAI, composite dietary antioxidant index.

**Table S33 Comparison of cortical gray matter volumes between dementia cases and non-dementia cases.**

| **Cortical gray matter volumes** | Overall | Non-dementia | Dementia | p value |
| --- | --- | --- | --- | --- |
| N | 22563 | 22487 | 76 |  |
| left caudalanteriorcingulate | 3110.76±575.13 | 3111.44±575.19 | 2909.47±521.33 | 0 |
| left caudalmiddlefrontal | 7116.51±1146.09 | 7117.73±1145.85 | 6754.74±1167.53 | 0.01 |
| left cuneus | 4367.42±718.93 | 4367.36±718.76 | 4384.39±772.91 | 0.84 |
| left entorhinal | 1796.67±366.93 | 1797.16±366.76 | 1651.53±390.55 | <0.001 |
| left fusiform | 9113.38±1234.98 | 9115.76±1234.69 | 8406.89±1117.67 | <0.001 |
| left inferiorparietal | 12597.30±1834.63 | 12601.42±1832.63 | 11378.32±2023.88 | <0.001 |
| left inferiortemporal | 12543.21±1681.67 | 12547.04±1680.76 | 11410.04±1572.68 | <0.001 |
| left isthmuscingulate | 2737.63±446.95 | 2738.07±446.87 | 2605.63±453.66 | 0.01 |
| left lateraloccipital | 12525.66±1758.00 | 12526.96±1756.99 | 12141.33±2010.70 | 0.06 |
| left lateralorbitofrontal | 9216.11±1018.46 | 9217.41±1018.63 | 8831.09±893.33 | <0.001 |
| left lingual | 6604.79±1140.18 | 6604.97±1139.71 | 6551.82±1278.31 | 0.69 |
| left medialorbitofrontal | 5024.21±613.32 | 5024.97±613.19 | 4799.01±616.53 | 0 |
| left middletemporal | 14084.65±1947.04 | 14088.91±1946.52 | 12823.33±1684.06 | <0.001 |
| left parahippocampal | 2147.64±328.25 | 2148.10±328.03 | 2013.53±366.39 | <0.001 |
| left paracentral | 4629.36±612.98 | 4629.74±613.00 | 4515.62±600.07 | 0.11 |
| left parsopercularis | 4545.74±724.59 | 4547.04±724.51 | 4159.96±642.16 | <0.001 |
| left parsorbitalis | 2265.79±316.00 | 2266.27±315.80 | 2124.88±344.83 | <0.001 |
| left parstriangularis | 4513.81±756.78 | 4515.08±756.69 | 4137.61±689.26 | <0.001 |
| left pericalcarine | 2128.80±440.44 | 2128.87±440.50 | 2107.64±424.64 | 0.68 |
| left postcentral | 11676.47±1520.90 | 11678.09±1521.70 | 11196.21±1174.56 | 0.01 |
| left posteriorcingulate | 3603.55±533.63 | 3604.25±533.63 | 3397.72±496.42 | <0.001 |
| left precentral | 14039.43±1674.23 | 14041.51±1674.43 | 13424.76±1500.55 | 0 |
| left precuneus | 10345.53±1345.97 | 10348.38±1345.65 | 9500.78±1169.64 | <0.001 |
| left rostralanteriorcingulate | 3750.50±637.16 | 3751.24±637.39 | 3532.45±523.80 | 0 |
| left rostralmiddlefrontal | 11637.42±1732.50 | 11640.18±1732.48 | 10821.59±1543.84 | <0.001 |
| left superiorfrontal | 25471.70±3015.17 | 25476.87±3015.04 | 23942.26±2658.57 | <0.001 |
| left superiorparietal | 11442.24±1603.91 | 11444.27±1603.43 | 10841.51±1642.09 | 0 |
| left superiortemporal | 17312.99±2032.75 | 17317.61±2031.61 | 15948.04±1919.72 | <0.001 |
| left supramarginal | 10946.58±1730.03 | 10949.06±1730.50 | 10213.12±1413.34 | <0.001 |
| left transversetemporal | 1244.15±229.36 | 1244.33±229.35 | 1193.33±228.14 | 0.05 |
| left insula | 6415.30±686.61 | 6415.84±686.75 | 6253.07±629.03 | 0.04 |
| right caudalanteriorcingulate | 2236.35±570.10 | 2236.80±570.12 | 2105.04±552.99 | 0.04 |
| right caudalmiddlefrontal | 6589.51±1111.25 | 6591.05±1111.30 | 6133.28±1000.94 | <0.001 |
| right cuneus | 3980.77±646.53 | 3980.63±646.07 | 4021.95±772.80 | 0.58 |
| right entorhinal | 1720.35±347.16 | 1720.79±347.08 | 1588.70±348.19 | <0.001 |
| right fusiform | 8904.42±1269.32 | 8906.86±1268.83 | 8182.72±1215.61 | <0.001 |
| right inferiorparietal | 15040.67±2155.10 | 15045.89±2152.42 | 13498.17±2396.67 | <0.001 |
| right inferiortemporal | 12721.25±1712.10 | 12724.89±1711.58 | 11645.72±1524.27 | <0.001 |
| right isthmuscingulate | 2583.92±416.28 | 2584.40±416.42 | 2443.57±346.22 | 0 |
| right lateraloccipital | 12856.05±1873.34 | 12857.14±1871.99 | 12535.13±2227.56 | 0.14 |
| right lateralorbitofrontal | 9204.16±1000.74 | 9205.42±1000.60 | 8831.01±979.22 | 0 |
| right lingual | 6655.49±1143.70 | 6654.99±1143.32 | 6806.05±1250.64 | 0.25 |
| right medialorbitofrontal | 4892.84±576.54 | 4893.79±576.29 | 4612.63±586.48 | <0.001 |
| right middletemporal | 14286.64±1818.70 | 14290.93±1817.20 | 13016.47±1826.89 | <0.001 |
| right parahippocampal | 2044.05±285.41 | 2044.22±285.35 | 1994.74±302.37 | 0.13 |
| right paracentral | 4480.88±627.32 | 4481.34±627.49 | 4346.29±562.43 | 0.06 |
| right parsopercularis | 4658.88±733.00 | 4659.80±733.19 | 4389.04±620.75 | 0 |
| right parsorbitalis | 2329.78±354.64 | 2330.28±354.55 | 2180.68±352.44 | <0.001 |
| right parstriangularis | 4171.97±716.55 | 4172.88±716.45 | 3901.59±699.72 | <0.001 |
| right pericalcarine | 2332.19±468.00 | 2332.20±467.76 | 2330.30±536.94 | 0.97 |
| right postcentral | 10871.60±1479.63 | 10872.85±1479.90 | 10500.01±1356.29 | 0.03 |
| right posteriorcingulate | 3614.80±557.68 | 3615.68±557.31 | 3352.70±606.66 | <0.001 |
| right precentral | 13505.09±1656.39 | 13506.60±1656.28 | 13057.76±1640.42 | 0.02 |
| right precuneus | 11094.15±1412.99 | 11096.74±1412.54 | 10330.17±1343.74 | <0.001 |
| right rostralanteriorcingulate | 2674.49±530.75 | 2675.08±530.64 | 2498.67±538.43 | 0 |
| right rostralmiddlefrontal | 11743.08±1823.18 | 11744.99±1823.11 | 11177.34±1765.09 | 0.01 |
| right superiorfrontal | 28320.63±3431.08 | 28326.75±3430.19 | 26508.72±3227.61 | <0.001 |
| right superiorparietal | 11567.79±1599.66 | 11570.57±1599.42 | 10746.99±1461.62 | <0.001 |
| right superiortemporal | 16415.46±1780.79 | 16419.48±1779.50 | 15224.87±1774.79 | <0.001 |
| right supramarginal | 10301.88±1488.73 | 10304.42±1488.44 | 9548.30±1390.45 | <0.001 |
| right transversetemporal | 985.75±173.35 | 985.90±173.28 | 942.20±190.34 | 0.03 |
| right insula | 6627.92±708.12 | 6628.53±708.29 | 6447.21±634.86 | 0.03 |

**Table S34 Comparison of subcortical gray matter volumes between dementia cases and non-dementia cases.**

| **Subcortical gray matter volumes** | Overall | Non-dementia | Dementia | p value |
| --- | --- | --- | --- | --- |
| N | 22563 | 22487 | 76 |  |
| left Cortex | 247340.34±24276.15 | 247390.64±24270.40 | 232456.89±21345.70 | <0.001 |
| left CerebralWhiteMatter | 238535.45±28527.09 | 238571.09±28516.70 | 227991.45±29822.43 | 0.001 |
| left Inf-Lat-Vent | 455.62±317.77 | 453.66±313.33 | 1033.22±777.19 | <0.001 |
| left Cerebellum-White-Matter | 15936.25±2142.04 | 15939.10±2141.96 | 15094.21±2009.77 | <0.001 |
| left Cerebellum-Cortex | 55200.83±5756.06 | 55203.42±5757.54 | 54435.85±5278.24 | 0.246 |
| left Thalamus-Proper | 6880.01±722.63 | 6881.15±722.35 | 6542.18±730.03 | <0.001 |
| left Caudate | 3284.65±431.22 | 3284.76±431.15 | 3252.73±452.87 | 0.518 |
| left Putamen | 4543.39±543.66 | 4544.37±543.42 | 4253.50±541.28 | <0.001 |
| left Pallidum | 2028.26±228.63 | 2028.47±228.52 | 1967.23±254.29 | 0.02 |
| left Hippocampus | 3945.87±409.67 | 3947.41±408.78 | 3490.55±421.48 | <0.001 |
| left Amygdala | 1548.63±225.51 | 1549.33±225.14 | 1341.43±240.41 | <0.001 |
| left Accumbens-area | 404.95±89.62 | 405.16±89.55 | 340.77±87.86 | <0.001 |
| left VentralDC | 3967.13±416.52 | 3967.74±416.35 | 3786.56±430.81 | <0.001 |
| left choroid-plexus | 697.79±248.88 | 697.24±248.73 | 862.80±237.87 | <0.001 |
| right Cortex | 248037.09±24312.30 | 248085.75±24305.33 | 233638.08±22132.32 | <0.001 |
| right CerebralWhiteMatter | 239697.78±28784.32 | 239734.77±28773.73 | 228753.00±30009.41 | <0.001 |
| right Inf-Lat-Vent | 444.92±295.21 | 442.92±289.37 | 1036.61±869.99 | <0.001 |
| right Cerebellum-White-Matter | 15244.69±2216.88 | 15247.42±2217.29 | 14436.59±1942.40 | 0.001 |
| right Cerebellum-Cortex | 57083.99±6178.77 | 57087.09±6179.05 | 56165.23±6065.24 | 0.194 |
| right Thalamus-Proper | 6767.56±686.36 | 6768.54±686.18 | 6478.05±684.14 | <0.001 |
| right Caudate | 3418.49±450.96 | 3418.74±450.99 | 3343.39±439.62 | 0.146 |
| right Putamen | 4608.44±543.84 | 4609.49±543.42 | 4298.19±583.68 | <0.001 |
| right Pallidum | 1988.11±228.56 | 1988.34±228.46 | 1921.72±250.38 | 0.011 |
| right Hippocampus | 4119.03±431.24 | 4120.61±430.27 | 3650.77±466.02 | <0.001 |
| right Amygdala | 1737.10±227.90 | 1737.71±227.69 | 1556.86±219.80 | <0.001 |
| right Accumbens-area | 481.70±83.41 | 481.91±83.32 | 421.03±86.94 | <0.001 |
| right VentralDC | 3890.48±402.35 | 3891.08±402.13 | 3711.77±428.36 | <0.001 |
| right choroid-plexus | 693.14±245.19 | 692.56±245.03 | 864.84±234.47 | <0.001 |
| BrainSeg (whole brain) | 1206623.48±116958.80 | 1206752.82±116959.02 | 1168356.25±111165.34 | 0.004 |
| BrainSegNotVent (whole brain) | 1173609.89±112920.95 | 1173801.67±112899.20 | 1116867.37±105452.21 | <0.001 |
| BrainSegNotVentSurf (whole brain) | 1173619.37±112798.70 | 1173812.78±112775.61 | 1116392.28±105475.54 | <0.001 |
| SubCortGray (whole brain) | 55203.52±4822.83 | 55213.88±4819.48 | 52136.93±4866.46 | <0.001 |
| TotalGray (whole brain) | 665522.68±59136.88 | 665637.36±59127.30 | 631589.84±52085.09 | <0.001 |
| SupraTentorial (whole brain) | 1061243.66±106801.91 | 1061366.31±106801.43 | 1024953.88±101244.62 | 0.003 |
| SupraTentorialNotVent (whole brain) | 1032586.61±103166.52 | 1032768.06±103144.24 | 978898.45±96087.58 | <0.001 |
| EstimatedTotalIntraCranial (whole brain) | 1551518.76±152477.04 | 1551549.41±152450.16 | 1542450.39±161036.78 | 0.604 |
| VentricleChoroid (whole brain) | 28657.09±15633.29 | 28598.29±15562.86 | 46055.63±24466.58 | <0.001 |
| Brain-Stem (whole brain) | 22057.92±2490.85 | 22058.54±2489.75 | 21876.15±2808.77 | 0.524 |
| WM-hypointensities (whole brain) | 2296.44±2974.31 | 2285.94±2949.04 | 5401.46±6635.59 | <0.001 |
| non-WM-hypointensities (whole brain) | 0.10±3.11 | 0.10±3.11 | 0.15±0.77 | 0.882 |
| Optic-Chiasm (whole brain) | 187.03±61.37 | 186.94±61.35 | 212.61±64.20 | <0.001 |
| CC-Posterior (whole brain) | 1050.23±158.75 | 1050.32±158.60 | 1021.74±198.42 | 0.117 |
| CC-Mid-Posterior (whole brain) | 540.10±110.04 | 540.20±109.90 | 507.87±142.22 | 0.011 |
| CC-Central (whole brain) | 521.53±117.00 | 521.66±116.93 | 484.76±132.53 | 0.006 |
| CC-Mid-Anterior (whole brain) | 524.00±123.45 | 524.20±123.40 | 464.78±124.40 | <0.001 |
| CC-Anterior (whole brain) | 943.82±163.67 | 943.90±163.81 | 918.43±112.16 | 0.176 |
| Volume-ratio of BrainSegVol-to-eTIV (whole brain) | 0.78±0.03 | 0.78±0.03 | 0.76±0.03 | <0.001 |
| Volume-ratio of MaskVol-to-eTIV (whole brain) | 1.04±0.03 | 1.04±0.03 | 1.03±0.03 | 0.058 |

**Table S35** **Baseline demographic and clinical of 22563 participants in brain structure analyses**

| **Characteristic** | **Overall** | **lower group**  **(CDAI≤1.579)** | **higher group**  **(CDAI＞1.579)** | **p value** |
| --- | --- | --- | --- | --- |
| **N** | 22563 | 15687 | 6876 |  |
| **Age, years, M(IQR)** | 56.00 (49.00-61.00) | 56.00 (49.00-61.00) | 56.00 (49.00-62.00) | <0.001 |
| **Sex, n (%)** |  |  |  | <0.001 |
| Female | 12008 (53.22%) | 8841 (56.36%) | 3167 (46.06%) |  |
| Male | 10555 (46.78%) | 6846 (43.64%) | 3709 (53.94%) |  |
| **Race, n(%)** |  |  |  | 0.099 |
| White | 20851 (92.41%) | 14514 (92.52%) | 6337 (92.16%) |  |
| Mixed | 642 (2.85%) | 461 (2.94%) | 181 (2.63%) |  |
| Asian | 806 (3.57%) | 534 (3.40%) | 272 (3.96%) |  |
| Other | 264 (1.17%) | 178 (1.13%) | 86 (1.25%) |  |
| **Education, M(IQR)** | 20.00 (10.00-20.00) | 19.00 (10.00-20.00) | 20.00 (13.00-20.00) | <0.001 |
| **TDI, M(IQR)** | -2.63 (-3.89--0.49) | -2.64 (-3.90--0.49) | -2.60 (-3.88--0.48) | 0.534 |
| **BMI, kgm^−2^,** **M(IQR)** | 25.73 (23.43-28.50) | 25.77 (23.43-28.53) | 25.63 (23.39-28.43) | 0.157 |
| **APOE-ε4 carrier, n (%)** |  |  |  | 0.344 |
| Non-carrier | 16384 (72.61%) | 11432 (72.88%) | 4952 (72.02%) |  |
| Carrier | 6179 (27.39%) | 4255 (27.13%) | 1924 (27.98%) |  |
| **Smoking, n(%)** |  |  |  | 0.74 |
| Never | 13816 (61.23%) | 9629 (61.38%) | 4187 (60.89%) |  |
| Previous | 7556 (33.49%) | 5228 (33.33%) | 2328 (33.86%) |  |
| Current | 1191 (5.28%) | 830 (5.29%) | 361 (5.25%) |  |
| **Alcohol, n(%)** |  |  |  | 0.219 |
| Never | 520 (2.30%) | 379 (2.42%) | 141 (2.05%) |  |
| Previous | 470 (2.08%) | 322 (2.05%) | 148 (2.15%) |  |
| Current | 21573 (95.61%) | 14986 (95.53%) | 6587 (95.80%) |  |
| **Diabetes, n(%)** |  |  |  | 0.434 |
| No | 22230 (98.52%) | 15462 (98.57%) | 6768 (98.43%) |  |
| Yes | 333 (1.48%) | 225 (1.43%) | 108 (1.57%) |  |
| **Hypertension, n(%)** |  |  |  | 0.465 |
| No | 19434 (86.13%) | 13529 (86.24%) | 5905 (85.88%) |  |
| Yes | 3129 (13.87%) | 2158 (13.76%) | 971 (14.12%) |  |
| **Hyperlipemia, n(%)** |  |  |  | 0.777 |
| No | 22174 (98.28%) | 15414 (98.26%) | 6760 (98.31%) |  |
| Yes | 389 (1.72%) | 273 (1.74%) | 116 (1.69%) |  |
| **CVD, n(%)** |  |  |  | 0.37 |
| No | 22055 (97.75%) | 15343 (97.81%) | 6712 (97.61%) |  |
| Yes | 508 (2.25%) | 344 (2.19%) | 164 (2.39%) |  |
| **Stroke, n(%)** |  |  |  | 0.462 |
| No | 22530 (99.85%) | 15666 (99.87%) | 6864 (99.83%) |  |
| Yes | 33 (0.15%) | 21 (0.13%) | 12 (0.17%) |  |
| **Cancer, n(%)** |  |  |  | 0.271 |
| No | 21298 (94.39%) | 14790 (94.28%) | 6508 (94.65%) |  |
| Yes | 1265 (5.61%) | 897 (5.72%) | 368 (5.35%) |  |
| **supplements, n(%)** |  |  |  | 0.004 |
| No | 15568 (69.00%) | 10917 (69.59%) | 4651 (67.64%) |  |
| Yes | 6995 (31.00%) | 4770 (30.41%) | 2225 (32.36%) |  |
| **Energy(kcal**), **M(IQR)** | 2003.46 (1685.07-2361.68) | 1842.83 (1577.21-2117.96) | 2445.19 (2148.02-2815.64) | <0.001 |

Abbreviations: CDAI, composite dietary antioxidant index; M(IQR), median (interquartile range); CVD, cardiovascular disease; TDI, Townsend Deprivation Index; BMI, Body mass index.

**Table S36 Correlation between CDAI and volume of cortical regions in the overall participants (n=22563)**

| **Volume** | **Estimate** | **Std. Error** | **t value** | **Pr(>\|t\|)** |
| --- | --- | --- | --- | --- |
| left caudalanteriorcingulate | 0.196 | 1.460 | 0.134 | 0.893 |
| left caudalmiddlefrontal | -5.176 | 2.920 | -1.773 | 0.076 |
| left cuneus | 0.572 | 1.815 | 0.315 | 0.752 |
| left entorhinal | 0.740 | 0.950 | 0.779 | 0.436 |
| left fusiform | 2.810 | 3.010 | 0.933 | 0.351 |
| left inferiorparietal | 5.232 | 4.609 | 1.135 | 0.256 |
| left inferiortemporal | 1.949 | 4.048 | 0.482 | 0.630 |
| left isthmuscingulate | -0.070 | 1.088 | -0.064 | 0.949 |
| left lateraloccipital | 1.943 | 4.202 | 0.462 | 0.644 |
| left lateralorbitofrontal | 3.837 | 2.402 | 1.597 | 0.110 |
| left lingual | 5.430 | 2.914 | 1.863 | 0.062 |
| left medialorbitofrontal | 2.894 | 1.501 | 1.928 | 0.054 |
| left middletemporal | 5.236 | 4.595 | 1.139 | 0.255 |
| left parahippocampal | 0.470 | 0.861 | 0.546 | 0.585 |
| left paracentral | 3.857 | 1.558 | 2.476 | 0.013 |
| left parsopercularis | 1.321 | 1.845 | 0.716 | 0.474 |
| left parsorbitalis | 1.222 | 0.786 | 1.554 | 0.120 |
| left parstriangularis | 0.214 | 1.905 | 0.112 | 0.911 |
| left pericalcarine | 0.954 | 1.147 | 0.832 | 0.405 |
| left postcentral | 5.615 | 3.784 | 1.484 | 0.138 |
| left posteriorcingulate | -0.755 | 1.344 | -0.562 | 0.574 |
| left precentral | 6.187 | 4.062 | 1.523 | 0.128 |
| left precuneus | 5.022 | 3.266 | 1.538 | 0.124 |
| left rostralanteriorcingulate | 0.114 | 1.568 | 0.073 | 0.942 |
| left rostralmiddlefrontal | -1.080 | 4.134 | -0.261 | 0.794 |
| left superiorfrontal | 8.907 | 7.012 | 1.270 | 0.204 |
| left superiorparietal | 3.961 | 4.080 | 0.971 | 0.332 |
| left superiortemporal | -0.177 | 4.819 | -0.037 | 0.971 |
| left supramarginal | 2.057 | 4.188 | 0.491 | 0.623 |
| left transversetemporal | 0.253 | 0.598 | 0.423 | 0.672 |
| left insula | 0.000 | 1.632 | 0.000 | 1.000 |
| right caudalanteriorcingulate | 1.952 | 1.509 | 1.294 | 0.196 |
| right caudalmiddlefrontal | -1.859 | 2.851 | -0.652 | 0.514 |
| right cuneus | 0.281 | 1.617 | 0.173 | 0.862 |
| right entorhinal | 1.847 | 0.905 | 2.042 | 0.041 |
| right fusiform | 1.167 | 3.012 | 0.387 | 0.698 |
| right inferiorparietal | 9.455 | 5.198 | 1.819 | 0.069 |
| right inferiortemporal | 3.189 | 4.097 | 0.778 | 0.436 |
| right isthmuscingulate | 0.076 | 1.044 | 0.073 | 0.942 |
| right lateraloccipital | -0.973 | 4.427 | -0.220 | 0.826 |
| right lateralorbitofrontal | 2.157 | 2.358 | 0.915 | 0.360 |
| right lingual | 7.120 | 2.948 | 2.415 | 0.016 |
| right medialorbitofrontal | 2.817 | 1.417 | 1.988 | 0.047 |
| right middletemporal | 6.158 | 4.288 | 1.436 | 0.151 |
| right parahippocampal | 0.884 | 0.748 | 1.181 | 0.237 |
| right paracentral | -0.009 | 1.588 | -0.006 | 0.996 |
| right parsopercularis | 1.486 | 1.858 | 0.800 | 0.424 |
| right parsorbitalis | 2.027 | 0.886 | 2.288 | 0.022 |
| right parstriangularis | 0.459 | 1.802 | 0.255 | 0.799 |
| right pericalcarine | 0.102 | 1.216 | 0.084 | 0.933 |
| right postcentral | -0.176 | 3.729 | -0.047 | 0.962 |
| right posteriorcingulate | 1.917 | 1.415 | 1.355 | 0.175 |
| right precentral | 1.271 | 4.058 | 0.313 | 0.754 |
| right precuneus | 2.455 | 3.384 | 0.726 | 0.468 |
| right rostralanteriorcingulate | -0.181 | 1.345 | -0.134 | 0.893 |
| right rostralmiddlefrontal | -0.900 | 4.370 | -0.206 | 0.837 |
| right superiorfrontal | 5.382 | 7.922 | 0.679 | 0.497 |
| right superiorparietal | 4.925 | 4.035 | 1.221 | 0.222 |
| right superiortemporal | -1.753 | 4.308 | -0.407 | 0.684 |
| right supramarginal | 5.439 | 3.693 | 1.473 | 0.141 |
| right transversetemporal | 0.067 | 0.452 | 0.147 | 0.883 |
| right insula | 0.459 | 1.668 | 0.275 | 0.783 |

Model was adjusted for the variables in Model 3, as described in Table S6. Bonferroni adjusted, p<0.05/62= 8.06E-4

**Table S37 Correlation between CDAI and volume of subcortical regions in the overall participants(n=22563)**

| **Subcortical volume** | Estimate | Std. Error | t value | P value |
| --- | --- | --- | --- | --- |
| left Cortex | 67.35 | 53.67 | 1.25 | 0.210 |
| left CerebralWhiteMatter | 90.84 | 64.10 | 1.42 | 0.156 |
| left Inf-Lat-Vent | -0.76 | 0.75 | -1.02 | 0.306 |
| left Cerebellum-White-Matter | 2.06 | 5.34 | 0.38 | 0.700 |
| left Cerebellum-Cortex | 40.08 | 13.35 | 3.00 | 0.003 |
| left Thalamus-Proper | 3.84 | 1.68 | 2.29 | 0.022 |
| left Caudate | -1.25 | 1.10 | -1.13 | 0.259 |
| left Putamen | -0.84 | 1.29 | -0.65 | 0.515 |
| left Pallidum | 0.47 | 0.55 | 0.85 | 0.398 |
| left Hippocampus | 0.81 | 0.97 | 0.84 | 0.401 |
| left Amygdala | 0.33 | 0.52 | 0.63 | 0.527 |
| left Accumbens-area | 0.31 | 0.21 | 1.44 | 0.149 |
| left VentralDC | 0.62 | 0.94 | 0.66 | 0.508 |
| left choroid-plexus | -0.90 | 0.56 | -1.62 | 0.105 |
| right Cortex | 56.64 | 53.70 | 1.05 | 0.292 |
| right CerebralWhiteMatter | 102.04 | 64.41 | 1.58 | 0.113 |
| right Inf-Lat-Vent | -1.63 | 0.71 | -2.28 | 0.022 |
| right Cerebellum-White-Matter | -0.95 | 5.64 | -0.17 | 0.867 |
| right Cerebellum-Cortex | 32.88 | 14.12 | 2.33 | 0.020 |
| right Thalamus-Proper | 2.79 | 1.57 | 1.77 | 0.076 |
| right Caudate | -1.31 | 1.15 | -1.14 | 0.253 |
| right Putamen | -0.05 | 1.30 | -0.04 | 0.968 |
| right Pallidum | 0.83 | 0.56 | 1.49 | 0.137 |
| right Hippocampus | 1.55 | 1.03 | 1.51 | 0.131 |
| right Amygdala | -0.58 | 0.53 | -1.09 | 0.275 |
| right Accumbens-area | 0.25 | 0.20 | 1.23 | 0.220 |
| right VentralDC | 1.07 | 0.91 | 1.18 | 0.239 |
| right choroid-plexus | -0.49 | 0.54 | -0.92 | 0.357 |
| BrainSeg | 385.86 | 248.17 | 1.55 | 0.120 |
| BrainSegNotVent | 448.97 | 239.69 | 1.87 | 0.061 |
| BrainSegNotVentSurf | 398.54 | 239.13 | 1.67 | 0.096 |
| SubCortGray | 7.69 | 10.63 | 0.72 | 0.469 |
| TotalGray | 204.68 | 125.09 | 1.64 | 0.102 |
| SupraTentorial | 265.49 | 229.43 | 1.16 | 0.247 |
| SupraTentorialNotVent | 325.00 | 221.65 | 1.47 | 0.143 |
| EstimatedTotalIntraCranial | 631.50 | 331.92 | 1.90 | 0.057 |
| VentricleChoroid | -59.50 | 36.52 | -1.63 | 0.103 |
| Brain-Stem | 6.36 | 5.77 | 1.10 | 0.270 |
| WM-hypointensities | -27.61 | 7.47 | -3.70 | 0.000 |
| non-WM-hypointensities | 0.00 | 0.01 | 0.09 | 0.925 |
| Optic-Chiasm | 0.12 | 0.15 | 0.77 | 0.443 |
| CC-Posterior | 0.31 | 0.42 | 0.74 | 0.461 |
| CC-Mid-Posterior | 0.37 | 0.29 | 1.30 | 0.192 |
| CC-Central | 0.40 | 0.30 | 1.34 | 0.180 |
| CC-Mid-Anterior | 0.24 | 0.31 | 0.77 | 0.442 |
| CC-Anterior | 0.18 | 0.43 | 0.43 | 0.668 |
| Volume-ratio of BrainSegVol-to-eTIV | 0.00 | 0.00 | -0.74 | 0.459 |
| Volume-ratio of MaskVol-to-eTIV | 0.00 | 0.00 | -1.35 | 0.176 |

Model was adjusted for the variables in Model 3, as described in Table S6. Bonferroni adjusted, p<0.05/48= 1.04E-3.

**Table S38 Correlation between CDAI and volume of cortical regions in the lower CDAI group (CDAI≤1.579, n=15687)**

| **Volume** | **Estimate** | **Std. Error** | **t value** | **P value** |
| --- | --- | --- | --- | --- |
| left caudalanteriorcingulate | 2.94 | 2.62 | 1.13 | 2.61E-1 |
| left caudalmiddlefrontal | 11.89 | 5.26 | 2.26 | 2.39E-2 |
| left cuneus | 5.71 | 3.25 | 1.76 | 7.88E-2 |
| left entorhinal | 3.98 | 1.70 | 2.34 | 1.92E-2 |
| **left fusiform** | 18.83 | 5.38 | **3.50** | **4.66E-4** |
| left inferiorparietal | 25.77 | 8.24 | 3.13 | 1.78E-3 |
| left inferiortemporal | 22.93 | 7.25 | 3.16 | 1.57E-3 |
| left isthmuscingulate | 2.43 | 1.95 | 1.25 | 2.13E-1 |
| left lateraloccipital | 23.06 | 7.51 | 3.07 | 2.15E-3 |
| **left lateralorbitofrontal** | 15.00 | 4.30 | **3.49** | **4.89E-4** |
| left lingual | 11.26 | 5.19 | 2.17 | 2.99E-2 |
| left medialorbitofrontal | 7.94 | 2.69 | 2.96 | 3.12E-3 |
| left middletemporal | 25.88 | 8.26 | 3.13 | 1.72E-3 |
| left parahippocampal | 4.90 | 1.55 | 3.17 | 1.51E-3 |
| **left paracentral** | 12.57 | 2.79 | **4.51** | **6.59E-6** |
| left parsopercularis | 8.37 | 3.29 | 2.54 | 1.09E-2 |
| left parsorbitalis | 3.05 | 1.41 | 2.16 | 3.09E-2 |
| left parstriangularis | 2.46 | 3.41 | 0.72 | 4.70E-1 |
| left pericalcarine | 2.55 | 2.04 | 1.25 | 2.12E-1 |
| **left postcentral** | 25.33 | 6.77 | **3.74** | **1.85E-4** |
| left posteriorcingulate | 2.90 | 2.42 | 1.20 | 2.31E-1 |
| **left precentral** | 31.30 | 7.28 | **4.30** | **1.70E-5** |
| left precuneus | 16.69 | 5.85 | 2.85 | 4.34E-3 |
| left rostralanteriorcingulate | 5.29 | 2.79 | 1.89 | 5.84E-2 |
| left rostralmiddlefrontal | 14.02 | 7.39 | 1.90 | 5.78E-2 |
| **left superiorfrontal** | 52.20 | 12.50 | **4.18** | **2.98E-5** |
| left superiorparietal | 18.40 | 7.30 | 2.52 | 1.17E-2 |
| left superiortemporal | 17.61 | 8.59 | 2.05 | 4.05E-2 |
| left supramarginal | 20.93 | 7.51 | 2.79 | 5.33E-3 |
| left transversetemporal | 1.36 | 1.07 | 1.27 | 2.04E-1 |
| left insula | 7.42 | 2.92 | 2.54 | 1.11E-2 |
| right caudalanteriorcingulate | 5.87 | 2.67 | 2.20 | 2.82E-2 |
| right caudalmiddlefrontal | 4.29 | 5.12 | 0.84 | 4.02E-1 |
| right cuneus | 6.03 | 2.90 | 2.08 | 3.77E-2 |
| right entorhinal | 4.37 | 1.61 | 2.71 | 6.75E-3 |
| right fusiform | 11.81 | 5.40 | 2.19 | 2.88E-2 |
| right inferiorparietal | 31.11 | 9.30 | 3.34 | 8.29E-4 |
| right inferiortemporal | 19.77 | 7.36 | 2.69 | 7.23E-3 |
| right isthmuscingulate | 2.36 | 1.87 | 1.27 | 2.06E-1 |
| right lateraloccipital | 15.72 | 7.90 | 1.99 | 4.67E-2 |
| **right lateralorbitofrontal** | 14.69 | 4.22 | **3.48** | **5.01E-4** |
| right lingual | 15.23 | 5.22 | 2.92 | 3.55E-3 |
| **right medialorbitofrontal** | 9.42 | 2.52 | **3.74** | **1.88E-4** |
| right middletemporal | 24.60 | 7.69 | 3.20 | 1.39E-3 |
| **right parahippocampal** | 5.28 | 1.35 | **3.92** | **8.81E-5** |
| right paracentral | 5.88 | 2.85 | 2.06 | 3.91E-2 |
| right parsopercularis | 9.72 | 3.30 | 2.94 | 3.27E-3 |
| right parsorbitalis | 4.75 | 1.59 | 2.99 | 2.80E-3 |
| right parstriangularis | 4.30 | 3.22 | 1.34 | 1.82E-1 |
| right pericalcarine | 2.10 | 2.16 | 0.97 | 3.33E-1 |
| right postcentral | 16.74 | 6.64 | 2.52 | 1.17E-2 |
| right posteriorcingulate | 7.27 | 2.54 | 2.87 | 4.17E-3 |
| right precentral | 24.00 | 7.22 | 3.33 | 8.85E-4 |
| right precuneus | 14.61 | 6.02 | 2.43 | 1.53E-2 |
| right rostralanteriorcingulate | 4.36 | 2.40 | 1.82 | 6.92E-2 |
| right rostralmiddlefrontal | 18.03 | 7.77 | 2.32 | 2.04E-2 |
| right superiorfrontal | 42.05 | 14.22 | 2.96 | 3.10E-3 |
| right superiorparietal | 17.87 | 7.21 | 2.48 | 1.32E-2 |
| right superiortemporal | 22.11 | 7.69 | 2.88 | 4.03E-3 |
| right supramarginal | 18.68 | 6.59 | 2.84 | 4.56E-3 |
| right transversetemporal | 0.94 | 0.80 | 1.18 | 2.40E-1 |
| right insula | 7.16 | 2.98 | 2.41 | 1.61E-2 |

Model was adjusted for the variables in Model3, as described in Table S6. Bonferroni adjusted, p<0.05/62= 8.06E-4

**Table S39 Correlation between CDAI and volume of subcortical regions in the lower group (CDAI≤1.579, n=15687)**

| **subcortical volume(N=15687)** | **Estimate** | **Std. Error** | **t value** | **P value** |
| --- | --- | --- | --- | --- |
| left Cortex | 428.61 | 96.19 | 4.46 | 8.41E-6 |
| left CerebralWhiteMatter | 395.32 | 115.36 | 3.43 | 6.12E-4 |
| left Inf-Lat-Vent | -2.18 | 1.33 | -1.64 | 1.02E-1 |
| left Cerebellum-White-Matter | 5.78 | 9.64 | 0.60 | 5.49E-1 |
| left Cerebellum-Cortex | 74.64 | 23.87 | 3.13 | 1.77E-3 |
| **left Thalamus-Proper** | 12.94 | 3.01 | 4.29 | **1.77E-5** |
| left Caudate | 1.95 | 1.98 | 0.98 | 3.26E-1 |
| left Putamen | 1.55 | 2.33 | 0.66 | 5.06E-1 |
| left Pallidum | 2.79 | 1.00 | 2.80 | 5.05E-3 |
| **left Hippocampus** | 6.10 | 1.73 | 3.53 | 4.14E-4 |
| left Amygdala | 2.43 | 0.93 | 2.61 | 9.19E-3 |
| left Accumbens-area | 0.94 | 0.38 | 2.47 | 1.35E-2 |
| left VentralDC | 4.02 | 1.70 | 2.37 | 1.80E-2 |
| left choroid-plexus | -0.98 | 0.99 | -0.98 | 3.25E-1 |
| right Cortex | 388.70 | 95.94 | 4.05 | 5.11E-5 |
| right CerebralWhiteMatter | 404.79 | 115.74 | 3.50 | 4.71E-4 |
| right Inf-Lat-Vent | -1.97 | 1.25 | -1.57 | 1.17E-1 |
| right Cerebellum-White-Matter | 14.46 | 10.21 | 1.42 | 1.57E-1 |
| right Cerebellum-Cortex | 62.07 | 25.21 | 2.46 | 1.38E-2 |
| **right Thalamus-Proper** | 13.44 | 2.82 | 4.77 | **1.89E-6** |
| right Caudate | 3.01 | 2.06 | 1.46 | 1.44E-1 |
| right Putamen | 2.66 | 2.34 | 1.14 | 2.55E-1 |
| right Pallidum | 3.12 | 1.00 | 3.11 | 1.85E-3 |
| **right Hippocampus** | 6.09 | 1.83 | 3.32 | **9.05E-4** |
| right Amygdala | 1.45 | 0.95 | 1.53 | 1.26E-1 |
| **right Accumbens-area** | 1.40 | 0.36 | 3.87 | **1.08E-4** |
| right VentralDC | 4.11 | 1.63 | 2.52 | 1.17E-2 |
| right choroid-plexus | -1.38 | 0.96 | -1.44 | 1.50E-1 |
| BrainSeg | 1861.89 | 445.92 | 4.18 | 2.99E-5 |
| BrainSegNotVent | 1918.95 | 430.76 | 4.45 | 8.45E-6 |
| BrainSegNotVentSurf | 1845.72 | 429.24 | 4.30 | 1.72E-5 |
| SubCortGray | 66.23 | 19.06 | 3.47 | 5.13E-4 |
| TotalGray | 1025.61 | 223.96 | 4.58 | 4.70E-6 |
| SupraTentorial | 1632.68 | 411.85 | 3.96 | 7.40E-5 |
| SupraTentorialNotVent | 1688.39 | 397.94 | 4.24 | 2.22E-5 |
| EstimatedTotalIntraCranial | 2913.02 | 593.75 | 4.91 | 9.38E-7 |
| VentricleChoroid | -55.69 | 65.00 | -0.86 | 3.92E-1 |
| Brain-Stem | 22.48 | 10.34 | 2.17 | 2.98E-2 |
| WM-hypointensities | -41.32 | 13.65 | -3.03 | 2.47E-3 |
| non-WM-hypointensities | 0.02 | 0.02 | 0.94 | 3.46E-1 |
| Optic-Chiasm | -0.27 | 0.27 | -1.01 | 3.13E-1 |
| CC-Posterior | 2.55 | 0.75 | 3.39 | 6.99E-4 |
| CC-Mid-Posterior | 1.30 | 0.51 | 2.53 | 1.13E-2 |
| CC-Central | 1.04 | 0.54 | 1.93 | 5.31E-2 |
| CC-Mid-Anterior | 0.91 | 0.57 | 1.61 | 1.08E-1 |
| CC-Anterior | 2.77 | 0.77 | 3.58 | 3.49E-4 |
| Volume-ratio of BrainSegVol-to-eTIV | -0.0002 | 0.0002 | -1.5493 | 1.21E-1 |
| Volume-ratio of MaskVol-to-eTIV | -0.0002 | 0.0002 | -1.3061 | 1.92E-1 |

Model was adjusted for the variables in Model 3, as described in Table S6. Bonferroni adjusted, p<0.05/48= 1.04E

**Table S40 Correlation between CDAI and volume of cortical regions in the higher group (CDAI＞1.579, n=6876)**

| **Volume** | **Estimate** | **Std. Error** | **t value** | **P value** |
| --- | --- | --- | --- | --- |
| left caudalanteriorcingulate | -1.26 | 2.85 | -0.44 | 6.59E-1 |
| left caudalmiddlefrontal | -15.82 | 5.62 | -2.81 | 4.93E-3 |
| left cuneus | 1.36 | 3.56 | 0.38 | 7.03E-1 |
| left entorhinal | 0.41 | 1.86 | 0.22 | 8.24E-1 |
| left fusiform | -8.88 | 5.91 | -1.50 | 1.33E-1 |
| left inferiorparietal | -9.06 | 9.03 | -1.00 | 3.16E-1 |
| left inferiortemporal | -10.75 | 7.90 | -1.36 | 1.74E-1 |
| left isthmuscingulate | -0.16 | 2.13 | -0.07 | 9.41E-1 |
| left lateraloccipital | -8.19 | 8.25 | -0.99 | 3.21E-1 |
| left lateralorbitofrontal | -3.77 | 4.69 | -0.80 | 4.22E-1 |
| left lingual | 1.56 | 5.78 | 0.27 | 7.87E-1 |
| left medialorbitofrontal | 1.13 | 2.93 | 0.39 | 6.99E-1 |
| left middletemporal | -14.46 | 8.91 | -1.62 | 1.05E-1 |
| left parahippocampal | -0.89 | 1.67 | -0.53 | 5.97E-1 |
| left paracentral | -0.59 | 3.05 | -0.19 | 8.47E-1 |
| left parsopercularis | -5.55 | 3.65 | -1.52 | 1.28E-1 |
| left parsorbitalis | -0.29 | 1.52 | -0.19 | 8.47E-1 |
| left parstriangularis | -2.72 | 3.74 | -0.73 | 4.67E-1 |
| left pericalcarine | -0.79 | 2.27 | -0.35 | 7.27E-1 |
| left postcentral | -4.07 | 7.40 | -0.55 | 5.82E-1 |
| left posteriorcingulate | -3.12 | 2.59 | -1.21 | 2.28E-1 |
| left precentral | -6.28 | 7.93 | -0.79 | 4.29E-1 |
| left precuneus | -3.76 | 6.38 | -0.59 | 5.55E-1 |
| left rostralanteriorcingulate | -6.20 | 3.10 | -2.00 | 4.54E-2 |
| left rostralmiddlefrontal | -10.43 | 8.10 | -1.29 | 1.98E-1 |
| left superiorfrontal | -11.26 | 13.84 | -0.81 | 4.16E-1 |
| left superiorparietal | -10.68 | 8.00 | -1.33 | 1.82E-1 |
| left superiortemporal | -13.06 | 9.50 | -1.37 | 1.69E-1 |
| left supramarginal | -6.15 | 8.17 | -0.75 | 4.51E-1 |
| left transversetemporal | 0.04 | 1.17 | 0.04 | 9.70E-1 |
| left insula | -7.76 | 3.20 | -2.42 | 1.54E-2 |
| right caudalanteriorcingulate | -0.50 | 3.02 | -0.17 | 8.69E-1 |
| right caudalmiddlefrontal | -5.71 | 5.54 | -1.03 | 3.03E-1 |
| right cuneus | -1.36 | 3.15 | -0.43 | 6.65E-1 |
| right entorhinal | -0.13 | 1.78 | -0.08 | 9.40E-1 |
| right fusiform | -11.60 | 5.87 | -1.98 | 4.83E-2 |
| right inferiorparietal | -8.93 | 10.16 | -0.88 | 3.80E-1 |
| right inferiortemporal | -11.65 | 7.96 | -1.46 | 1.43E-1 |
| right isthmuscingulate | -0.76 | 2.05 | -0.37 | 7.12E-1 |
| right lateraloccipital | -25.10 | 8.71 | -2.88 | 3.97E-3 |
| right lateralorbitofrontal | -8.95 | 4.62 | -1.94 | 5.27E-2 |
| right lingual | 1.51 | 5.90 | 0.26 | 7.98E-1 |
| right medialorbitofrontal | -2.40 | 2.80 | -0.86 | 3.92E-1 |
| right middletemporal | -3.69 | 8.34 | -0.44 | 6.58E-1 |
| right parahippocampal | -1.11 | 1.45 | -0.77 | 4.43E-1 |
| right paracentral | -4.77 | 3.09 | -1.54 | 1.23E-1 |
| right parsopercularis | -2.93 | 3.69 | -0.79 | 4.27E-1 |
| right parsorbitalis | 0.09 | 1.73 | 0.05 | 9.58E-1 |
| right parstriangularis | -2.04 | 3.55 | -0.58 | 5.65E-1 |
| right pericalcarine | -2.02 | 2.41 | -0.84 | 4.01E-1 |
| right postcentral | -13.54 | 7.38 | -1.83 | 6.66E-2 |
| right posteriorcingulate | -3.77 | 2.76 | -1.37 | 1.71E-1 |
| right precentral | -14.54 | 8.05 | -1.81 | 7.08E-2 |
| right precuneus | -4.10 | 6.70 | -0.61 | 5.41E-1 |
| right rostralanteriorcingulate | -2.22 | 2.65 | -0.84 | 4.02E-1 |
| right rostralmiddlefrontal | -14.03 | 8.67 | -1.62 | 1.06E-1 |
| right superiorfrontal | -26.26 | 15.42 | -1.70 | 8.87E-2 |
| right superiorparietal | -8.31 | 7.92 | -1.05 | 2.94E-1 |
| right superiortemporal | -17.94 | 8.49 | -2.11 | 3.46E-2 |
| right supramarginal | -2.78 | 7.28 | -0.38 | 7.03E-1 |
| right transversetemporal | -1.29 | 0.90 | -1.43 | 1.53E-1 |
| right insula | -8.98 | 3.29 | -2.73 | 6.32E-3 |

Model was adjusted for the variables in Model 3, as described in Table S6. Bonferroni adjusted, p<0.05/62= 8.06E-4

**Table S41 Correlation between CDAI and volume of subcortical regions in the higher group (CDAI＞1.579, n=6876)**

| **subcortical volume** | Estimate | Std. Error | t value | P value |
| --- | --- | --- | --- | --- |
| left Cortex | -154.63 | 104.52 | -1.48 | 1.39E-1 |
| left CerebralWhiteMatter | -228.33 | 123.82 | -1.84 | 6.52E-2 |
| left Inf-Lat-Vent | 1.47 | 1.48 | 0.99 | 3.21E-1 |
| left Cerebellum-White-Matter | -9.45 | 10.28 | -0.92 | 3.58E-1 |
| left Cerebellum-Cortex | -9.09 | 26.21 | -0.35 | 7.29E-1 |
| left Thalamus-Proper | -3.92 | 3.25 | -1.21 | 2.28E-1 |
| left Caudate | -4.06 | 2.15 | -1.89 | 5.89E-2 |
| left Putamen | -4.70 | 2.49 | -1.89 | 5.90E-2 |
| left Pallidum | -2.20 | 1.08 | -2.03 | 4.21E-2 |
| left Hippocampus | -4.70 | 1.91 | -2.46 | 1.41E-2 |
| left Amygdala | -1.88 | 1.02 | -1.84 | 6.56E-2 |
| left Accumbens-area | -0.45 | 0.41 | -1.09 | 2.75E-1 |
| left VentralDC | -2.18 | 1.82 | -1.20 | 2.31E-1 |
| left choroid-plexus | -0.59 | 1.09 | -0.54 | 5.87E-1 |
| right Cortex | -205.06 | 105.37 | -1.95 | 5.17E-2 |
| right CerebralWhiteMatter | -214.57 | 124.81 | -1.72 | 8.56E-2 |
| right Inf-Lat-Vent | -0.28 | 1.44 | -0.19 | 8.47E-1 |
| right Cerebellum-White-Matter | -14.67 | 10.76 | -1.36 | 1.73E-1 |
| right Cerebellum-Cortex | -24.35 | 27.77 | -0.88 | 3.81E-1 |
| right Thalamus-Proper | -4.56 | 3.06 | -1.49 | 1.35E-1 |
| right Caudate | -3.93 | 2.23 | -1.76 | 7.77E-2 |
| right Putamen | -4.27 | 2.50 | -1.71 | 8.76E-2 |
| right Pallidum | -1.82 | 1.08 | -1.68 | 9.22E-2 |
| right Hippocampus | -3.89 | 2.03 | -1.91 | 5.61E-2 |
| right Amygdala | -2.67 | 1.04 | -2.58 | 1.00E-2 |
| right Accumbens-area | -0.75 | 0.39 | -1.93 | 5.41E-2 |
| right VentralDC | -1.72 | 1.76 | -0.98 | 3.28E-1 |
| right choroid-plexus | -0.003 | 1.058 | -0.003 | 9.98E-1 |
| BrainSeg | -960.83 | 480.63 | -2.00 | 4.56E-2 |
| BrainSegNotVent | -937.99 | 463.99 | -2.02 | 4.33E-2 |
| BrainSegNotVentSurf | -915.90 | 464.18 | -1.97 | 4.85E-2 |
| SubCortGray | -49.15 | 20.70 | -2.37 | 1.76E-2 |
| TotalGray | -451.38 | 244.16 | -1.85 | 6.45E-2 |
| SupraTentorial | -880.28 | 445.39 | -1.98 | 4.81E-2 |
| SupraTentorialNotVent | -857.27 | 430.05 | -1.99 | 4.63E-2 |
| EstimatedTotalIntraCranial | -1257.06 | 649.37 | -1.94 | 5.29E-2 |
| VentricleChoroid | -22.98 | 72.40 | -0.32 | 7.51E-1 |
| Brain-Stem | -18.82 | 11.26 | -1.67 | 9.47E-2 |
| WM-hypointensities | -25.65 | 13.90 | -1.84 | 6.51E-2 |
| non-WM-hypointensities | 0.00 | 0.01 | -0.61 | 5.40E-1 |
| Optic-Chiasm | -0.04 | 0.29 | -0.14 | 8.89E-1 |
| CC-Posterior | -1.96 | 0.84 | -2.35 | 1.90E-2 |
| CC-Mid-Posterior | -0.38 | 0.56 | -0.68 | 4.94E-1 |
| CC-Central | -0.25 | 0.59 | -0.43 | 6.65E-1 |
| CC-Mid-Anterior | -0.75 | 0.60 | -1.24 | 2.13E-1 |
| CC-Anterior | -1.68 | 0.83 | -2.03 | 4.21E-2 |
| Volume-ratio of BrainSegVol-to-eTIV | -0.00002 | 0.00016 | -0.10 | 9.18E-1 |
| Volume-ratio of MaskVol-to-eTIV | -0.00019 | 0.00017 | -1.12 | 2.62E-1 |

Model was adjusted for the variables in Model 3, as described in Table S6. Bonferroni adjusted, p<0.05/48= 1.04E-3

**Table S42 Longitudinal associations between gray matter volume and the risk of dementia in the lower group (CDAI≤1.579, n=15687)**

|  | All cause dementia | |  | Alzheimer’s dementia | |  | Vascular dementia | |
| --- | --- | --- | --- | --- | --- | --- | --- | --- |
|  | HR (95%CI) | P-value |  | HR (95%CI) | P-value |  | HR (95%CI) | P-value |
| left fusiform | 0.998(0.999-1.00) | **0.021** |  | 0.999(0.999-1.00) | 0.071 |  | 1.00(0.999-1.001) | 0.994 |
| left lateralorbitofrontal | 0.999(0.999-1.00) | 0.080 |  | 0.999(0.999-1.00) | **0.034** |  | 1.001(0.999-1.002) | 0.114 |
| left paracentral | 1.00(0.999-1.001) | 0.930 |  | 0.999(0.999-1.01) | 0.666 |  | 0.999(0.999-1.001) | 0.666 |
| left postcentral | 0.999(0.999-1.00) | 0.130 |  | 0.999(0.999-1.00) | **0.041** |  | 1.00(0.999-1.001) | 0.911 |
| left precentral | 0.999(0.999-1.00) | 0.213 |  | 0.999(0.999-1.00) | 0.260 |  | 1.00(0.999-1.001) | 0.993 |
| left superiorfrontal | 0.999(0.999-1.00) | 0.059 |  | 0.999(0.999-1.00) | **0.037** |  | 1.00(0.999-1.00) | 0.270 |
| right lateralorbitofrontal | 0.997(0.999-1.00) | **0.049** |  | 0.999(0.999-1.00) | 0.330 |  | 0.999(0.999-1.001) | 0.846 |
| right medialorbitofrontal | 0.999(0.998-1.00) | **0.019** |  | 0.999(0.999-1.00) | 0.224 |  | 0.999(0.999-1.001) | 0.141 |
| right parahippocampal | 1.00(0.999-1.001) | 0.504 |  | 1.00(0.999-1.002) | 0.953 |  | 1.001(0.998-1.003) | 0.657 |
| left Thalamus-Proper | 0.999(0.999-1.00) | **0.047** |  | 0.999(0.999-1.00) | 0.253 |  | 1.001(1.00-1.003) | 0.062 |
| left Hippocampus | 0.998(0.997-0.998) | **6.23E-12** |  | 0.997(0.996-0.998) | **3.85E-08** |  | 0.992(0.985-0.999) | 0.392 |
| right Thalamus-Proper | 0.999(0.999-1.00) | 0.088 |  | 0.999(0.999-1.00) | 0.310 |  | 1.001(0.999-1.003) | 0.161 |
| right Hippocampus | 0.997(0.997-0.998) | **6.44E-11** |  | 0.997(0.996-0.998) | **9.57E-07** |  | 1.001(0.999-1.003) | 0.805 |
| right Accumbens-area | 0.993(0.990-0.997) | **0.001** |  | 0.992(0.985-0.999) | **0.007** |  | 1.009(0.999-1.003) | 0.082 |

Model was adjusted for the variables in Model 3, as described in Table S6.

**Table S43The mediation effects of gray matter volume in the association between CDAI and incident dementia risk in the lower group (CDAI<=1.579, n=15687)**

|  | All cause dementia | | | | | | | |
| --- | --- | --- | --- | --- | --- | --- | --- | --- |
|  | **Direct effect** | | **Indirect effect** | | **Total effect** | | **Proportion of mediator** | ***P* value** |
|  | **Beta(95%CI)** | ***P* value** | **Beta(95%CI)** | ***P* value** | **Beta(95%CI)** | ***P* value** |  |  |
| left fusiform | 6.34E-4(-2.53E-4,2.41E-3) | 0.244 | -3.01E-5(-1.04E-4, -3.51E-6) | 0.012 | 6.05E-4(-2.63E-4,2.35E-3) | 0.266 | -4.05% | 0.266 |
| right lateralorbitofrontal | 6.20E-4(-3.90E-4,3.04E-3) | 0.30 | -2.82E-5(-1.01E-4, -1.39E-6) | 0.03 | 5.92E-4(-4.30E-4,2.98E-3) | 0.332 | -3.51% | 0.358 |
| right medialorbitofrontal | 5.54E-4(-2.19E-4,2.02E-3) | 0.244 | -3.01E-5(-6.88E-5, -5.39E-6) | 0.01 | 5.24E-4(-2.38E-4,2.00E-3) | 0.276 | -4.6% | 0.286 |
| left Thalamus-Proper | 7.31E-4(-2.85E-4,4.12E-3) | 0.254 | -3.79E-5(-1.57E-4, -1.28E-6) | 0.03 | 6.93E-4(-3.01E-4,4.01E-3) | 0.292 | -4.57% | 0.314 |
| left Hippocampus | 5.17E-4(-2.19E-4,1.71E-3) | 0.250 | -6.50E-5(-1.23E-4, -2.57E-5) | <2E-16 | 4.52E-4(-2.72E-4,1.64E-3) | 0.330 | -11.46% | 0.330 |
| right Hippocampus | 6.13E-4(-1.69E-4,1.95E-3) | 0.250 | -6.57E-5(-1.27E-4, -2.47E-5) | <2E-16 | 5.47E-4(-2.22E-4,1.82E-3) | 0.250 | -10.25% | 0.196 |
| right Accumbens-area | 5.81E-4(-2.13E-4,1.85E-3) | 0.238 | -4.57E-5(-9.38E-4, -1.37E-5) | <2E-16 | 5.35E-4(-2.52E-4,1.77E-3) | 0.296 | -7.10% | 0.296 |
|  | Alzheimer’s dementia | | | | | | | |
|  | **Direct effect** | | **Indirect effect** | | **Total effect** | | **Proportion of mediator** | ***P* value** |
|  | **Beta(95%CI)** | ***P* value** | **Beta(95%CI)** | ***P* value** | **Beta(95%CI)** | ***P* value** |  |  |
| left lateralorbitofrontal | 5.54E-4(-3.78E-4,2.23E-3) | 0.234 | -2.13E-5(-8.65E-4, 5.28E-7) | 0.058 | 5.71E-4(-2.77E-4,2.35E-3) | 0.43 | -2.41% | 0.428 |
| left postcentral | 5.31E-4(-2.43E-4,1.97E-3) | 0.286 | -2.14E-5(-6.38E-5, 8.16E-7) | 0.068 | 5.09E-4(-2.55E-4,1.93E-3) | 0.304 | -3.2% | 0.356 |
| left superiorfrontal | 8.49E-4(-2.48E-4,4.33E-3) | 0.228 | -2.77E-5(-9.75E-5, 8.39E-6) | 0.09 | 8.22E-4(-2.81E-4,4.24E-3) | 0.24 | -3.9% | 0.310 |
| left Hippocampus | 3.00E-3(-2.90E-5,1.95E-2) | 0.108 | -1.19E-5(-7.01E-4, -3.81E-6) | <2E-16 | 2.89E-3(-1.88E-5,1.83E-2) | 0.112 | -4.78% | 0.34 |
| right Hippocampus | 2.78E-3(-2.21E-5,1.68E-2) | 0.102 | -1.08E-4(-6.27E-4, -3.39E-6) | <2E-16 | 2.67E-3(-1.68E-5,1.62E-2) | 0.122 | -4.45% | 0.118 |
| right Accumbens-area | 1.43E-3(-1.35E-4,3.63E-3) | 0.062 | -4.63E-5(-1.17E-4, -1.04E-5) | 0.006 | 1.39E-3(-1.07E-4,3.54E-3) | 0.052 | -3.42% | 0.128 |

Model was adjusted for the variables in Model 3, as described in Table S6. CDAI, composite dietary antioxidant index.
